# Parallel Palladium-Catalyzed Synthesis of Carboxylic Acids from Aryl Iodides, Bromides, and Vinyl Triflates Using Acetic Anhydride and Formate Anion as an External Condensed Source of Carbon Monoxide

**DOI:** 10.3390/molecules30153298

**Published:** 2025-08-06

**Authors:** Antonia Iazzetti, Giancarlo Fabrizi, Yuri Gazzilli, Antonella Goggiamani, Federico Marrone, Chen Shen, Roberta Zoppoli

**Affiliations:** 1 Dipartimento di Scienze Biotecnologiche di Base, Cliniche Intensivologiche e Perioperatorie, Università Cattolica del Sacro Cuore, L. go Francesco Vito 1, 00168 Rome, Italy; antonia.iazzetti@unicatt.it; 2 Policlinico Universitario ‘A. Gemelli’ Foundation-IRCCS, 00168 Rome, Italy; 3 Dipartimento di Chimica e Tecnologie del Farmaco, Sapienza, Università di Roma, P. le A. Moro 5, 00185 Rome, Italy; giancarlo.fabrizi@uniroma1.it (G.F.); yuri.gazzilli@uniroma1.it (Y.G.); federico.marrone@uniroma1.it (F.M.); chen.shen@uniroma1.it (C.S.); roberta.zoppoli@uniroma1.it (R.Z.)

**Keywords:** palladium catalysis, carbon monoxide, carboxylic acid, combinatorial chemistry

## Abstract

Aryl iodides, bromides and vinyl-triflates are usually converted in high to excellent yields into the corresponding carboxylic acids through a parallel palladium-catalyzed hydroxycarbonylation using lithium formate and acetic anhydride as external condensed source of carbon monoxide.

## 1. Introduction

Carboxylic acids are large volume products and their palladium-catalyzed preparation from aryl and vinyl halides or triflates in the presence of carbon monoxide as the carbonyl source is a key step in many laboratory-scale and industrial processes, including the synthesis of biologically active compounds [1,2,3,4,5,6]. The procedure shows a broad functional group tolerance, is quite simple from an operative point of view, and is usually efficient. In addition, carbon monoxide is a cheap, readily available, and reactive reagent. However, the method possesses some major disadvantages: carbon monoxide is a highly toxic gas, which presents real problems in storage and transportation and, when used, carefully assembled gas delivery systems are required to avoid losses and ensure safety. These disadvantages may limit the broader applicability of this hydroxycarbonylation protocol. Furthermore, the widespread utilization of combinatorial chemistry [7,8] and high-throughput screening [9,10] in pharmaceutical and agrochemical research laboratories has created a demand for new technologies compatible with the automated handling of liquids and solids rather than those requiring advanced gas delivery systems. As a consequence, the utilization of common palladium-catalyzed reactions of organic halides or triflates in the presence of carbon monoxide using gas delivery systems is usually impractical in the synthesis of compound libraries.

This limitation is particularly evident in modern research environments, where high-throughput screening and combinatorial chemistry require methods that are compatible with parallel synthesis, miniaturization, and automated liquid and solid handling systems.

While palladium-catalyzed carbonylation using CO remains a powerful transformation in traditional synthetic chemistry, its reliance on toxic gas and specialized equipment renders it ill-suited to the demands of small-scale, library-oriented synthesis. To address this, considerable efforts have been made, particularly in the past few years, to develop alternative strategies where carbonylation reactions can be conducted without the direct use of carbon monoxide [11,12], most notably the use of carbon monoxide surrogates such as formic acid, oxalyl chloride, molybdenum hexacarbonyl, and other in situ CO-releasing reagents. One notable advancement is a two-chamber system introduced by Skrydstrup and colleagues [13], enabling the generation and immediate consumption of CO in a sealed setup. This technique led to the development of 9-methylfluorene-9-carbonyl chloride (COgen), which releases carbon monoxide in the presence of a palladium catalyst. These alternatives improve safety and ease of handling and enhance scalability and compatibility with automation. As a result, they represent a more practical and efficient solution for compound library synthesis in medicinal and agrochemical research.

**Scheme 1:**
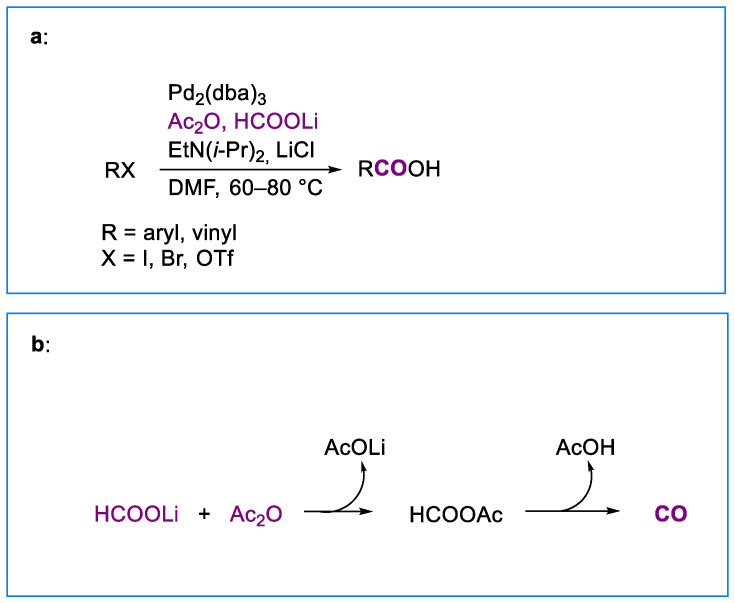
Previous study. (**a**) Our previous study; (**b**) formation of acetic formic anhydride and its thermal decomposition.

In this context, we showed that the palladium-catalyzed hydroxycarbonylation of aryl and vinyl halides or triflates can be conveniently carried out in the presence of formate salts and acetic anhydride under the conditions displayed in Scheme 1a, omitting phosphine ligands [14,15,16]. Furthermore, the procedure for synthesizing benzoic acids from aryl bromides using our source of carbon monoxide has been improved [17].

The reaction involves the formation of acetic formic anhydride (generated in situ by the reaction of formate anion with acetic anhydride), which acts as a condensed source of carbon monoxide, followed by its thermal decomposition [18] to give carbon monoxide (Scheme 1b). Carboxylic acids are obtained under mild conditions in good to high isolated yields with various vinyl triflates and aryl iodides, including, in the latter case, those containing ethers, ketones, esters, and nitro groups. The presence of *ortho* substituents does not hamper the reaction. The procedure is particularly useful in cases where the direct utilization of pressured carbon monoxide is not viable.

**Scheme 2:**
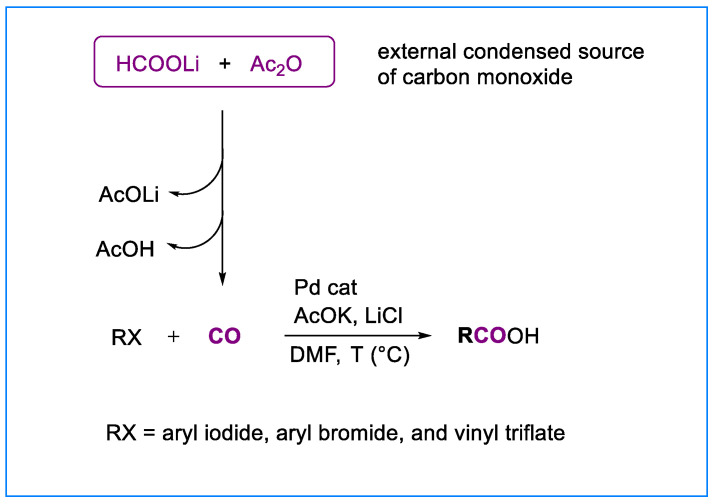
This study.

However, with aryl iodides containing nucleophilic centers, the reaction failed to yield the desired products. For example, when *m*-iodobenzyl alcohol was subjected to our standard hydroxycarbonylation conditions, a complex reaction mixture, which we have not further investigated, was obtained. Therefore, we thought that the development of a methodology involving the utilization of formate salts and acetic anhydride as a condensed source of carbon monoxide external to the reaction vessel containing organic halides or triflates and the catalyst system might provide, in these cases, cleaner reaction mixtures and possibly higher yields. Herein we report just such a protocol, which is applied to the palladium-catalyzed hydroxycarbonylation of aryl iodides, bromides, and vinyl triflates (Scheme 2).

## 2. Results and Discussion

Ethyl *p*-iodobenzoate **1a** was selected as the model system when this research began. Lithium formate (12 equiv) and acetic anhydride (8 equiv) were added to anhydrous DMF (2.5 mL) in a reaction vessel, which was connected by a silicon tube to the reactor containing 1 equiv of ethyl *p*-iodobenzoate, 0.025 equiv of Pd_2_(dba)_3_, 3 equiv of lithium chloride [19], and 2 equiv of potassium acetate in 1.5 mL of DMF. Both reaction vessels were warmed at 80 °C for 24 h. After this time, *p*-ethoxycarbonylbenzoic acid **2a** was isolated in 88% yield. Potassium acetate was added to the reaction mixture because of the key role of acetate anions in our hydroxycarbonylation reaction [14]. It appears that acetate anions react with σ-acylpalladium intermediates formed in situ to afford mixed anhydrides [20,21,22], which are converted into the corresponding carboxylic acids via hydrolysis. Placing lithium formate and acetic anhydride in a separate reaction vessel removes acetate anions (generated in the sequence producing carbon monoxide; see Scheme 1b) from the reaction mixture. Thus, neither mixed anhydrides nor carboxylic acids are formed. When ethyl *p*-iodobenzoate was subjected to the same hydroxycarbonylation conditions, omitting potassium acetate, the corresponding carboxylic acid **2a** was observed in trace amounts.

Interestingly, the hydroxycarbonylation of ethyl *p*-iodobenzoate using 0.025 equiv of Pd_2_(dba)_3_, 3 equiv of lithium chloride in DMF, and 10 equiv of water produced *p*-ethoxycarbonylbenzoic acid in almost the same yield (86%) as obtained with potassium acetate [omitting lithium chloride led to a remarkable decrease in the yield (53%)]. However, this procedure did not produce yields as high with several aryl iodides as it did with our model system and *m*-iodobenzyl alcohol (*vide infra*). For instance, the hydroxycarbonylation of *p*-iodotoluene **1b** in the presence of water yielded the desired carboxylic acid in 69% yield, whereas a significantly higher yield of 98% was obtained when potassium acetate was used instead. A combination of water (10 equiv.) and potassium acetate (2 equiv.) was also tested, affording the desired carboxylic acid **2b** in 71% yield. Upon reducing the catalyst loading to 1 mol% Pd, **2b** was obtained in 67% yield—lower than that achieved with 5 mol% Pd—and the reaction time doubled. Therefore, the potassium acetate-based protocol was adopted as the standard procedure for the parallel synthesis of carboxylic acids from various aryl iodides, although it may be advisable to evaluate the effectiveness of each protocol on a case-by-case basis (*equipment details are provided in the General Experimental Procedures*).

Benzoic acids were usually isolated in high to excellent yield. Only with *p*-iodoanisole **1d**, lithium chloride was omitted. Indeed, according to our previous observations [14], *p*-iodoanisole gave the highest yield in the absence of lithium chloride. Furthermore, *m*-iodobenzyl alcohol **1e** was best converted into the corresponding carboxylic acid using the procedure based on the utilization of DMF and water. Acid-base extraction was also evaluated as a purification method; however, the resulting products exhibited ~90–95% purity (as determined by ^1^H NMR) due to residual by-products. Therefore, chromatographic purification was employed to ensure higher purity. Our preparative results are summarized in Table 1.

This hydroxycarbonylation methodology was next extended to vinyl triflates **3** and, using the same standard conditions employed with aryl iodides but at lower reaction temperature (60 °C); seven α,β-unsaturated carboxylic acids **4** were prepared in high yields (Table 2). Even in this case, the procedure based on the utilization of potassium acetate was proved to be usually superior to the one using water. For example, when **3c** was subject to hydroxycarbonylation in the presence of 0.025 equiv of Pd_2_(dba)_3_ and 3 equiv of lithium chloride in DMF and 10 equiv of water, the corresponding carboxylic acid **4c** was formed only in 52% yield.

Once the external, condensed carbon monoxide source protocol had been successfully established for aryl iodides and vinyl triflates, the methodology was extended to aryl bromides **5** under slightly modified reaction conditions based on those previously reported by Bessmernykh [16]. Specifically, the hydroxycarbonylation reactions were conducted using 1 equivalent of aryl bromide **5**, 2 equivalents of KOAc, 0.05 equivalents of Pd(OAc)_2_, and 0.05 equivalents of dppf in DMF at 120 °C for 24 h. Carbon monoxide was generated in situ at the same temperature from the HCOOLi/Ac_2_O system.

Also in this case, the methodology shows broad substrate generality and affords the expected acids with good yields starting from neutral, electron-poor, and electron-rich aryl bromides (Table 3).

Under the current conditions at different temperature, alkynyl halides and alkyl halides/triflates are unreactive. Further studies will be required to overcome side reactions such as dimerization and β-hydride elimination, and to expand the methodology to these substrate classes.

Building on the success of our protocol that employs formate salts and acetic anhydride as a convenient, external condensed source of carbon monoxide, we aimed to extend its applicability to carbon-13 isotopic labeling. Isotopically labeled compounds, particularly ^13^C-labeled carboxylic acids, are of growing importance in pharmaceutical and agrochemical research. They serve as indispensable tools in drug metabolism and pharmacokinetics (DMPK) studies, where the incorporation of a carbon isotope “fingerprint” enables precise tracking of bioactive compounds and their metabolites through analytical methods such as NMR spectroscopy and mass spectrometry.

**Scheme 3:**
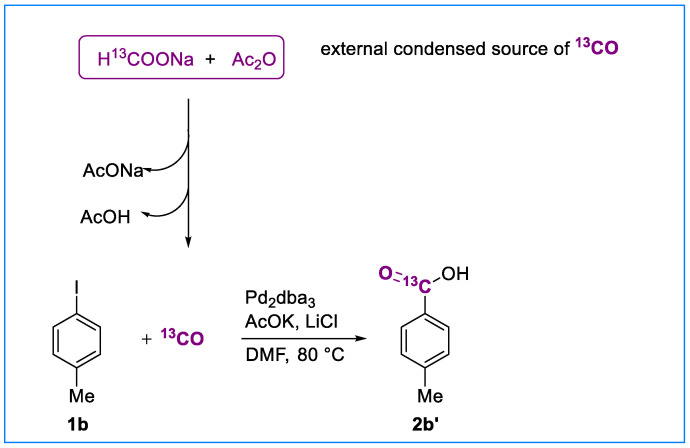
Synthesis of ^13^C-labeled *p*-toluic acid.

Despite their significant value, the synthesis of ^13^C-labeled carboxylic acids remains challenging due to the limited availability of labeled reagents, the high cost of isotopes, and the operational complexity associated with their incorporation, particularly when ^13^CO is required. To overcome these limitations, we employed commercially available H^13^COONa in combination with acetic anhydride. Under our optimized hydroxycarbonylation conditions, *p*-iodotoluene was converted to ^13^C-labeled *p*-toluic acid **2b’** in 60% yield via in situ generation of ^13^CO through the formation and thermal decomposition of ^13^C-acetic formic anhydride (Scheme 3).

This strategy avoids the direct handling of expensive and hazardous ^13^CO gas and represents a practical and cost-effective alternative to surrogates such as ^13^COgen [13,23]. Importantly, the reaction proceeds under mild conditions and is amenable to scale-up, offering a versatile platform for the preparation of ^13^C-labeled carboxylic acids.

To verify the scalability of our protocol, we performed a gram-scale reaction.

Lithium formate (60.0 mmol) and acetic anhydride (60.0 mmol) were added to anhydrous DMF (25 mL) in a reaction vessel, which was connected by a silicon tube to the reactor containing *p*-iodotoluene (5.0 mmol), Pd_2_(dba)_3_ (0.125 mmol), lithium chloride (15.0 mmol), and potassium acetate (10 mmol) in 15 mL of DMF. Both reaction vessels were warmed at 80 °C for 24 h, and *p*-toluic acid was isolated in 89% of yield.

## 3. Materials and Methods

### 3.1. General Information

All of the commercially available reagents, catalysts, bases, and solvents were used as purchased, without further purification. Salts were dried for 24 h at 70 °C under vacuum before utilization. Synthesized starting materials and reaction products were purified by flash chromatography using SiO_2_ as stationary phase, eluting with *n*-hexane/ethyl acetate mixtures. ^1^H NMR (400.13 MHz), ^13^C NMR (100.6 MHz), and ^19^F spectra (376.5 MHz) were recorded with a Bruker Avance 400 spectrometer equipped with a Nanobay console and Cryoprobe Prodigy probe (version software: TopSpin 3.6.5) (Bruker, Billerica, MA, USA). Splitting patterns are designated as s (singlet), d (doublet), t (triplet), q (quartet), m (multiplet), or bs (broad singlet). Melting points were determined with a Büchi B-545 apparatus and are uncorrected.

### 3.2. General Experimental Procedures

#### 3.2.1. Typical Procedure for the Parallel Synthesis of Carboxylic Acids

**Figure 1:**
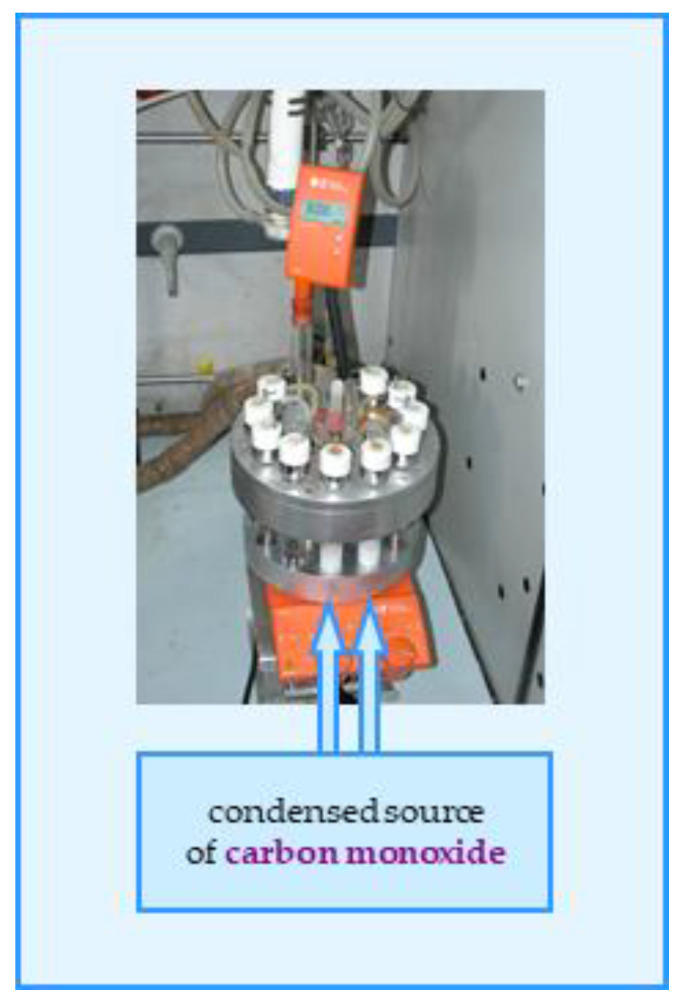
Setup for in situ CO generation from a condensed source via connected reactors.

Seven α,β-unsaturated carboxylic acids were prepared by using a Carousel (Radley Discovery Technologies, London, UK) as follows: two Carousel Tube Reactors were each charged with a solution of HCOOLi·H_2_O (1.469 g, 21.0 mmol) and acetic anhydride (1.322 mL, 14.0 mmol) in anhydrous DMF (9 mL). Then, seven Carousel Tube Reactors were each charged with a vinyl triflate (0.5 mmol), Pd_2_(dba)_3_ (11.4 mg, 0.0125 mmol), KOAc (98.1 mg, 1.0 mmol), LiCl (63.6 mg, 1.5 mmol) in DMF (1.5 mL). The two reactors containing lithium formate and acetic anhydride were then connected to the reactors containing aryl iodides through silicon tubes (Figure 1). All the reaction mixtures were stirred at 60 °C for 24 h. After cooling, each reaction mixture was diluted with ethyl acetate, washed with 2 N HCl, dried over Na_2_SO_4_, and concentrated under reduced pressure. The residues were purified by flash chromatography to give the corresponding carboxylic acids in 71–87% yields.

#### 3.2.2. Gram-Scale Synthesis of *p*-Toluic Acid

A high-pressure reactor was charged with HCOOLi H_2_O (3.851 g, 58.284 mmol), acetic anhydride (3.5 mL, 36.39 mmol), anhydrous DMF (25.0 mL), and connected via a stainless steel tube to a second reactor containing *p*-iodotoluene (1.0 g, 4.587 mmol), Pd_2_(dba)_3_ (104.9 mg, 0.115 mmol), lithium chloride (583.4 mg, 13.751 mmol), and potassium acetate (900.4 mg, 9.174 mmol) in 20.0 mL of DMF. Both reactions were heated at 80 °C for 24 h. After cooling to room temperature and degassing, the reaction mixture was diluted with ethyl acetate, washed with 2 N HCl, dried over Na_2_SO_4_, and concentrated under reduced pressure. The crude product was purified by flash chromatography (silica gel, 70 g; *n*-hexane/ethylacetate 80/20 *v*/*v*) to obtain a pale-yellow solid of **2b** (555.2 mg, 89% yield).

***Caution*:** Due to the potential generation of high pressure during the reaction, appropriate high-pressure equipment is required for gram-scale syntheses.

#### 3.2.3. Characterization Data of Starting Materials **3a**–**c**, **3e**–**f**

Vinyl triflates **3a**–**c**, **3e**–**f** were prepared according to ref. [24,25,26], and NMR data are in agreement with those reported previously in the literature [27,28,29,30,31,32,33,34,35]. Vinyl triflates **3d** and **3g** are commercially available.

(10*R*,13*S*,17*S*)-10,13-dimethyl-3-(((trifluoromethyl)sulfonyl)oxy)-2,7,8,9,10,11,12,13,14,15,16,17-dodecahydro-1*H*-cyclopenta[*a*]phenanthren-17-yl acetate **3a**: 81% yield; lit. [27] mp: 110–112 °C; mp: 112–114 °C; ^1^H NMR (400.13 MHz) (CDCl_3_): δ 5.98 (d, *J* = 1.5 Hz, 1H), 5.56 (d, *J* = 3.1 Hz, 1H), 4.60 (t, *J* = 8.5 Hz, 1H), 2.61–2.47 (m, 1H), 2.35 (dd, *J*_1_ = 18.0 Hz, *J*_2_ = 5.0 Hz, 1H), 2.28–2.12 (m, 2H), 2.04 (s, 3H), 1.91 (dd, *J*_1_ = 12.9 Hz, *J*_2_ = 5.5 Hz, 1H), 1.78 (dt, *J*_1_ = 12.5 Hz, *J*_2_ = 3.1 Hz, 1H), 1.73–1.28 (m, 9H), 1.21 (td, *J*_1_ = 25.8 Hz, *J*_2_ = 12.9 Hz, *J*_3_ = 4.0 Hz, 1H), 1.13–1.00 (m, 2H), 0.96 (s, 3H), 0.82 (s, 3H); ^13^C NMR (100.6 MHz) (CDCl_3_): δ 171.5 (C), 147.3 (C), 138.8 (C), 128.1 (CH), 120.7 (CH), 118.9 (q, *J*_C-F_ = 318.9 Hz, C), 82.8 (CH), 51.3 (CH), 48.0 (CH), 42.8 (C), 36.9 (CH_2_), 35.1 (CH_2_), 31.8 (CH), 27.8 (CH_2_), 25.8 (CH_2_), 23.7 (CH_2_), 21.4 (CH_3_), 21.0 (CH_2_), 18.9 (CH_3_), 12.3 (CH_3_); ^19^F NMR (376.5 MHz, CDCl_3_) δ −73.8 (s).
(3*S*,10*S*,13*S*)-10,13-dimethyl-17-(((trifluoromethyl)sulfonyl)oxy)-2,3,4,5,6,7,8,9,10,11,12,13,14,15-tetradecahydro-1*H*-cyclopenta[*a*]phenanthren-3-yl acetate **3b**: 70% yield; brown solid; lit. [28] mp: 82–83 °C; mp: 82–84 °C; ^1^H NMR (400.13 MHz) (CDCl_3_): δ 5.59−6.61 (m, 1H), 4.83−4.71 (m, 1H), 2.35−2.24 (m, 1H), 2.11 (s, 3H) 2.09−1.88 (m, 2H), 1.85−1.24 (m, 15H), 1.18−1.08 (m, 1H), 1.05 (s, 3H), 0.94 (s, 3H), 0.91−0.81 (m, 1H); ^13^C NMR (100.6 MHz) (CDCl_3_): δ 171.0 (C), 159.6 (C), 118.8 (q, *J*_C-F_ = 324.5 Hz, C), 114.1 (CH), 73.8 (CH), 54.9 (CH), 54.4 (CH), 45.1 (CH), 36.7 (CH_2_), 36.0 (C), 34.2 (CH_2_), 33.7 (C), 32.9 (CH_2_), 31.0 (CH_2_), 28.8 (CH_2_), 28.5 (CH_2_), 27.7 (CH_2_), 21.7 (CH_3_), 20.8 (CH_2_), 15.6 (CH_3_), 12.4 (CH_3_); ^19^F NMR (376.5 MHz, CDCl_3_) δ −73.6 (s).
(*E*)-cyclooct-1-en-1-yl trifluoromethanesulfonate **3c**: 68% yield; oil [29]; ^1^H NMR (400.13 MHz) (CDCl_3_): δ 5.68 (t, *J* = 8.6 Hz, 1H), 2.46 (t, *J* = 6.4 Hz, 2H), 2.23−2.09 (m, 2H), 1.75−1.67 (m, 2H), 1.65−1.52 (m, 6H); ^13^C NMR (100.6 MHz) (CDCl_3_): δ 151.2 (C), 120.9 (CH), 118.7 (q, *J*_C-F_ = 320.2 Hz, C), 29.8 (CH_2_), 29.4 (CH_2_), 27.4 (CH_2_), 26.1 (CH_2_), 25.8 (CH_2_), 25.2 (CH_2_); ^19^F NMR (376.5 MHz, CDCl_3_) δ −74.2 (s).
1,2,3,6-tetrahydro-[1,1′-biphenyl]-4-yl trifluoromethanesulfonate **3e**: 71% yield; oil [30]; ^1^H NMR (400.13 MHz) (CDCl_3_): δ 7.24–7.20 (m, 2H), 7.19–7.09 (m, 3H), 5.84–5.70 (m, 1H), 2.85–2.70 (m, 1H), 2.53–2.20 (m, 4H), 2.05–1.95 (m, 1H), 1.94–1.82 (1H); ^13^C NMR (100.6 MHz) (CDCl_3_): δ 149.3 (C), 144.8 (C), 128.9 (CH), 127.0 (CH), 126.9 (CH), 126.7 (CH), 118.7 (q, J_C-F_ = 320.0 Hz, C), 118.4 (C), 39.0 (CH_2_), 31.8 (CH_2_), 29.6 (CH_2_), 28.1 (CH_2_); ^19^F NMR (376.5 MHz, CDCl_3_) δ –73.9 (s).
4-(*tert*-butyl)cyclohex-1-en-1-yl trifluoromethanesulfonate **3f**: 78% yield; oil [31]; ^1^H NMR (400.13 MHz) (CDCl_3_): δ 5.80–5.66 (m, 1H), 2.45–2.27 (m, 2H), 2.25–2.15 (m, 1H), 2.01–1.87 (m, 2H), 1.45–1.26 (m, 2H), 0.89 (s, 9H); ^13^C NMR (100.6 MHz) (CDCl_3_): δ 149.5 (C), 118.9 (q, J_C-F_ = 319.1 Hz, C), 43.23 (CH), 32.4 (C), 28.8 (CH_2_), 27.5 (CH_3_), 25.6 (CH_2_), 24.4 (CH_2_); ^19^F NMR (376.5 MHz, CDCl_3_) δ –73.7 (s).

#### 3.2.4. Characterization Data of Final Compounds **2a**–**k**, **4a**–**g**

4-(ethoxycarbonyl)benzoic acid **2a**: 88% [ArI], 71% [ArBr] yield; lit. [14] mp: 168–170 °C; mp: 168–170 °C; ^1^H NMR (400.13 MHz) (DMSO-*d*_6_): δ 13.36 (bs, 1H), 8.12–8.00 (m, 4H), 4.35 (q, *J* = 7.0 Hz, 2H), 1.34 (t, *J* = 7.0 Hz, 3H); ^13^C NMR (100.6 MHz) (DMSO-*d*_6_): δ 167.5 (C), 166.0 (C),135.7 (C), 134.3 (C), 130.5 (CH), 130.2 (CH), 62.1 (CH_2_), 15.0 (CH_3_).
4-methylbenzoic acid **2b**: 98% [ArI], 78% [ArBr] yield; lit. [14] mp: 179–180 °C; mp: 179–180 °C; ^1^H NMR (400.13 MHz) (DMSO-*d*_6_): δ 12.79 (bs, 1H), 7.84 (d, *J* = 7.9 Hz, 2H), 7.30 (d, *J* = 7.9 Hz, 2H), 2.37 (s, 3H); ^13^C NMR (100.6 MHz) (DMSO-*d*_6_): δ 168.2 (C), 143.9 (C), 130.2 (CH),130.0 (CH), 128.9 (C), 22.0 (CH_3_).
3-methoxybenzoic acid **2c**: 88% [ArI], 72% [ArBr] yield; lit. [14] mp: 105–107 °C; mp: 105–107 °C; ^1^H NMR (400.13 MHz) (DMSO-*d*_6_): δ 13.00 (bs, 1H), 7.54 (d, *J* = 7.2 Hz, 1H), 7.48–7.37 (m, 2H), 7.19 (dd, *J*_1_ = 8.1 Hz, *J*_2_ = 2.4 Hz, 1H), 3.81 (s, 3H); ^13^C NMR (100.6 MHz) (DMSO-*d*_6_): δ 168.0 (C), 160.1 (C), 133.1 (C),130.6 (CH), 122.4 (CH), 119.8 (CH), 114.8 (CH), 56.1 (CH_3_).
4-methoxybenzoic acid **2d**: 96% [ArI], 69% [ArBr] yield; lit. [14] mp: 178–180 °C; mp: 178–180 °C; ^1^H NMR (400.13 MHz) (DMSO-*d*_6_): δ 12.62 (bs, 1H), 7.90 (d, *J* = 8.7 Hz, 2H), 7.02 (d, *J* = 8.7 Hz, 2H), 3.82 (s, 3H); ^13^C NMR (100.6 MHz) (DMSO-*d*_6_): δ 167.9 (C), 163.7 (C), 132.2 (CH),128.2 (C), 114.7 (CH), 56.3 (CH_3_).
3-(hydroxymethyl)benzoic acid **2e**: 60% yield; lit. [32] mp: 114–115 °C; mp: 112–115 °C; ^1^H NMR (400.13 MHz) (DMSO-*d*_6_): δ 12.90 (bs, 1H), 7.93 (s, 1H), 7.81 (d, *J* = 7.6 Hz, 1H), 7.55 (d, *J* = 7.6 Hz, 1H), 7.45 (t, *J* = 7.6 Hz, 1H), 5.31 (bs, 1H), 4.56 (s, 2H); ^13^C NMR (100.6 MHz) (DMSO-*d*_6_): δ 168.3 (C), 144.0 (C),131.7 (CH), 131.5 (C), 129.2 (CH), 128.5 (CH), 128.1 (CH), 63.3 (CH_2_).
4-methyl-2-nitrobenzoic acid **2f**: 82% yield; lit. [14] mp: 187–188 °C; mp: 187–188 °C; ^1^H NMR (400.13 MHz) (DMSO-*d*_6_): δ 13.53 (bs, 1H), 8.41 (d, *J* = 1.3 Hz, 1H), 8.12 (dd, *J*_1_ = 8.0 Hz, *J*_2_ = 1.3 Hz, 1H), 7.64 (d, *J* = 8.0 Hz, 1H), 2.59 (s, 3H); ^13^C NMR (100.6 MHz) (DMSO-d_6_): δ 166.4 (C), 149.7 (C),138.6 (C), 134.36 (CH), 134.35 (CH), 131.0 (C), 125.9 (CH), 20.6 (CH_3_).
2-methoxybenzoic acid **2g**: 70% yield; lit. [14] mp: 101–102 °C; mp: 101–102 °C; mp: 101–102 °C; ^1^H NMR (400.13 MHz) (DMSO-*d*_6_): δ 12.58 (bs, 1H), 7.63 (dd, *J*_1_ = 7.6 Hz, *J*_2_ = 1.6 Hz, 1H), 7.53–7.47 (m, 1H), 7.11 (d, *J* = 8.3 Hz, 1H), 6.99 (t, *J* = 7.3 Hz, 1H), 3.81 (s, 3H); ^13^C NMR (100.6 MHz) (DMSO-*d*_6_): δ 168.2 (C), 158.9 (C),133.9 (CH), 131.5 (CH), 122.2 (C), 120.9 (CH), 113.3 (CH), 56.6 (CH_3_).
4-acetylbenzoic acid **2h**: 82% [ArI], 70% [ArBr] yield; lit. [14] mp: 208–210 °C; mp: 208–210 °C; ^1^H NMR (400.13 MHz) (DMSO-*d*_6_): δ 13.31 (bs, 1H), 8.08–8.03 (m, 4H), 2.63 (s, 3H); ^13^C NMR (100.6 MHz) (DMSO-*d*_6_): δ 198.6 (C), 167.5 (C),140.7 (C), 135.4 (C), 130.4 (CH), 129.2 (CH), 27.9 (CH_3_).
benzoic acid **2i**: 71% yield; lit. [14] mp: 120–121 °C; mp: 120–121 °C, ^1^H NMR (400.13 MHz) (DMSO-*d*_6_): δ 12.96 (bs, 1H), 7.96 (d, *J* = 7.0 Hz, 2H), 7.63 (t, *J* = 7.2 Hz, 1H), 7.51 (t, *J* = 7.6 Hz, 2H); ^13^C NMR (100.6 MHz) (DMSO-*d*_6_): δ 168.2 (C), 133.8 (CH), 131.7 (CH), 130.2 (CH), 129.5 (CH).
[1,1′-biphenyl]-4-carboxylic acid **2j**: 70% yield; lit. [14] mp: 223–224 °C; mp: 223–224 °C; ^1^H NMR (400.13 MHz) (DMSO-*d*_6_): δ 12.99 (bs, 1H), 8.03 (d, *J* = 8.4 Hz, 2H), 7.80 (d, *J* = 7.2 Hz, 2H), 7.73 (d, *J* = 7.2 Hz, 2H), 7.50 (t, *J* = 7.7 Hz, 2H), 7.45–7.39 (m, 1H); ^13^C NMR (100.6 MHz) (DMSO-*d*_6_): δ 168.0 (C), 145.2 (C), 139.9 (C),130.8 (CH), 130.5 (C), 130.0 (CH), 129.2 (C), 127.9 (CH), 127.7 (CH).
4-nitrobenzoic acid **2k**: 75% yield; lit. [14] mp: 237–239 °C; mp: 237–239 °C; ^1^H NMR (400.13 MHz) (DMSO-*d*_6_): δ 13.65 (bs, 1H), 8.33 (d, *J* = 8.9 Hz, 2H), 8.17 (d, *J* = 8.9 Hz, 2H); ^13^C NMR (100.6 MHz) (DMSO-*d*_6_): δ 166.7 (C), 150.9 (C), 137.3 (C), 131.6 (CH), 124.6 (CH).
4-methylbenzoic acid **2b’**: 60% yield; lit. [14] mp: 179–180 °C; mp: 179–180 °C; ^1^H NMR (400.13 MHz) (DMSO-*d*_6_): δ 12.85 (bs, 1H), 7.89 (dd, *J*_1_ = 3.6 Hz, *J*_2_ = 8.2 Hz, 2H), 7.36 (d, *J* = 7.9 Hz, 2H), 2.43 (s, 3H); ^13^C NMR (100.6 MHz) (DMSO-*d*_6_): δ 168.2 (enriched C), 143.9 (C), 130.2 (d, *J* = 2.3 Hz, CH), 130.0 (d, *J* = 4.4 Hz, CH), 128.9 (d, *J* = 72.0 Hz, CH), 22.0 (CH_3_).
(10*R*,13*S*,17*S*)-17-acetoxy-10,13-dimethyl-2,7,8,9,10,11,12,13,14,15,16,17-dodecahydro-1*H*-cyclopenta[*a*]phenanthrene-3-carboxylic acid **4a**: 71% yield; lit. [14] mp: 177–178 °C; mp: 177–178 °C; ^1^H NMR (400.13 MHz) (CDCl_3_): δ 7.12–7.09 (m, 1H), 5.89–5.80 (m, 1H), 4.60 (t, *J* = 8.4 Hz, 1H), 2.50 (dd, *J*_1_ = 18.4 Hz, *J*_2_ = 5.7 Hz, 1H), 2.20–2.12 (m, 3H), 2.04 (s, 3H), 1.89 (dd, *J*_1_ = 12.9 Hz, *J*_2_ = 4.5 Hz, 1H), 1.80–0.98 (m, 13H), 0.91 (s, 3H), 0.82 (s, 3H); ^13^C NMR (100.6 MHz) (CDCl_3_): δ 173.4 (C), 171.6 (C), 141.6 (C), 140.9 (CH), 133.0 (CH), 125.2 (C), 82.9 (CH), 51.4 (CH), 48.2 (CH), 42.8 (C), 36.9 (CH_2_), 35.0 (C), 33.6 (CH_2_), 32.2 (CH_2_), 31.8 (CH), 27.8 (CH_2_), 23.7 (CH_2_), 21.7 (CH_2_), 21.5 (CH_3_), 20.8 (CH_2_), 19.3 (CH_3_), 12.1 (CH_3_).
(3*S*,10*S*,13*S*)-3-acetoxy-10,13-dimethyl-2,3,4,5,6,7,8,9,10,11,12,13,14,15-tetradecahydro-1*H*-cyclopenta[*a*]phenanthrene-17-carboxylic acid **4b**: 81% yield; lit. [33] mp: 261–263 °C; mp: 259–261 °C; ^1^H NMR (400.13 MHz) (CDCl_3_): δ 7.32 (bs, 1H), 6.91–6.83 (m, 1H), 4.75–4.61 (m, 1H), 2.33–2.12 (m, 2H), 2.01 (s, 3H) 1.87–1.64 (m, 3H), 1.63–1.31 (m, 12H), 1.07–0.94 (m, 2H), 0.90 (s, 3H), 0.85 (s, 3H), 0.82–0.69 (m, 2H); ^13^C NMR (100.6 MHz) (CDCl_3_): δ 171.1 (C), 170.4 (C), 146.9 (CH), 146.5 (CH), 74.0 (CH), 56.7 (CH), 54.9 (CH), 46.1 (C), 45.1 (CH), 36.8 (CH_2_), 36.0 (C), 34.9 (CH_2_), 34.3 (CH_2_), 34.1 (CH), 32.2 (CH_2_), 32.0 (CH_2_), 28.7 (CH_2_), 27.7 (CH_2_), 21.7 (CH_3_), 21.2 (CH_2_), 16.3 (CH_3_), 12.4 (CH_3_).
(*E*)-cyclooct-1-ene-1-carboxylic acid **4c**: 71% yield; lit. [34] mp: 95 °C; mp: 94–96 °C; ^1^H NMR (400.13 MHz) (CDCl_3_): δ 7.14 (t, *J* = 8.5 Hz, 1H), 2.50–2.40 (m, 2H), 2.35–2.25 (m, 2H), 1.65–1.55 (m, 4H), 1.52–1.42 (m, 4H); ^13^C NMR (100.6 MHz) (CDCl_3_): δ 173.5 (C), 145.6 (CH), 133.0 (C), 29.3 (CH_2_), 29.2 (CH_2_), 27.7 (CH_2_), 26.8 (CH_2_), 26.2 (CH_2_), 24.7 (CH_2_).
3,3,5,5-tetramethylcyclohex-1-ene-1-carboxylic acid **4d**: 86% yield; lit. [35] mp: 154–156 °C; mp: 153–155 °C; ^1^H NMR (400.13 MHz) (CDCl_3_): δ 9.97 (bs), 6.86 (s, 1H), 2.02 (s, 2H), 1.35 (s, 2H), 1.08 (s, 6H), 0.96 (s, 6H); ^13^C NMR (100.6 MHz) (CDCl_3_): δ 173.9 (C), 150.6 (CH), 126.3 (C), 49.4 (CH_2_), 37.4 (CH_2_), 33.92 (C), 30.8 (CH_3_), 30.7 (C), 29.9 (CH_3_).
1,2,3,6-tetrahydro-[1,1′-biphenyl]-4-carboxylic acid **4e**: 82% yield; lit. [36] mp: 197–199 °C; mp: 195–197 °C; ^1^H NMR (400.13 MHz) (CDCl_3_): δ 7.23 (d, *J* = 7.2 Hz, 1H), 7.18–7.09 (m, 4H), 2.80–2.61 (m, 1H), 2.55–2.41 (m, 2H), 2.37–2.18 (m, 2H); ^13^C NMR (100.6 MHz) (CDCl_3_): δ 173.0 (C), 146.1 (C), 142.1 (CH),130.0 (C), 128.8 (CH), 127.1 (CH), 126.7 (CH), 39.3 (CH), 34.2 (CH_2_), 29.6 (CH_2_), 24.7 (CH_2_).
4-(tert-butyl)cyclohex-1-ene-1-carboxylic acid **4f**: 87% yield; lit. [36] mp: 182–183 °C; mp: 180–182 °C; ^1^H NMR (400.13 MHz) (CDCl_3_): δ 7.16–7.10 (m, 1H), 2.55–2.44 (m, 1H), 2.33–2.21 (1H), 2.19–2.05 (m, 1H), 2.01–1.86 (m, 2H), 1.33–1.23 (m, 1H), 1.13 (ddd, *J*_1_ = 12.2 Hz, *J*_2_ = 4.8 Hz, *J*_3_ = 2.4 Hz, 1H), 0.88 (s, 9H); ^13^C NMR (100.6 MHz) (CDCl_3_): δ 173.2.1 (C), 143.3 (CH), 129.9 (C), 43.5 (CH), 32.4 (C), 28.0 (CH_2_), 27.4 (CH_3_), 25.5 (CH_2_), 23.8 (CH_2_).
6-methoxy-3,4-dihydronaphthalene-1-carboxylic acid **4g**: 82% yield; lit. [14] mp: 108–109 °C; mp: 108–109 °C; ^1^H NMR (400.13 MHz) (CDCl_3_): δ 10.00 (bs, 1H), 7.78 (d, *J* = 8.6 Hz, 1H), 7.20 (t, *J* = 4.9 Hz, 1H), 6.69 (dd, *J*_1_ = 8.6 Hz, *J*_2_ = 2.6 Hz, 1H), 6.65 (d, *J* = 2.6 Hz, 1H), 3.73 (s, 3H), 2.67 (t, *J* = 7.6 Hz, 2H), 2.38–2.30 (m, 2H); ^13^C NMR (100.6 MHz) (CDCl_3_): δ 172.4 (C), 159.2 (C), 140.8 (CH),138.4 (C), 129.8 (C), 127.8 (CH), 123.8 (C), 113.9 (CH), 111.4 (CH), 55.5 (CH_3_), 28.2 (CH_2_), 23.9 (CH_2_).

## 4. Conclusions

In conclusion, we have demonstrated that lithium formate and acetic anhydride can serve as an efficient condensed external source of carbon monoxide for the palladium-catalyzed hydroxycarboxylation of aryl iodides, aryl bromides, and vinyl triflates. The reaction proceeds under mild conditions and generally affords carboxylic acids in high to excellent yields. This methodology is particularly valuable in settings where the use of pressurized CO is impractical, such as in parallel synthesis for the generation of compound libraries. Moreover, this approach provides a convenient and scalable route for the synthesis of ^13^C-labeled carboxylic acids.

## Figures and Tables

**Table 1:** Preparation of benzoic acids via parallel palladium-catalyzed hydroxycarbonylation of aryl iodides using the HCOOLi/Ac_2_O combination as external condensed source of carbon monoxide ^a^.

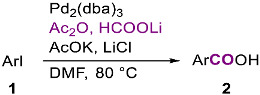					
Entry	Aryl Iodide 1		Benzoic Acid 2		Yield % ^b^
1	*p*-EtOOC-C_6_H_4_-I	**1a**	*p*-EtOOC-C_6_H_4_-COOH	**2a**	88
2	*p*-Me-C_6_H_4_-I	**1b**	*p*-Me-C_6_H_4_-COOH	**2b**	98
3	*m*-MeO-C_6_H_4_-I	**1c**	*m*-MeO-C_6_H_4_-COOH	**2c**	88
4	*p*-MeO-C_6_H_4_-I	**1d**	*p*-MeO-C_6_H_4_-COOH	**2d**	96 ^c^
5	*m*-HOCH_2_-C_6_H_4_-I	**1e**	*m*-HOCH_2_-C_6_H_4_-COOH	**2e**	60 ^d^
6	*o*-NO_2_-*p*-Me-C_6_H_3_-I	**1f**	*o*-NO_2_-*p*-Me-C_6_H_3_-COOH	**2f**	82
7	*o*-OMe-C_6_H_4_-I	**1g**	*o*-OMe-C_6_H_4_-COOH	**2g**	70
8	*p*-MeCO-C_6_H_4_-I	**1h**	*p*-MeCO-C_6_H_4_-COOH	**2h**	82

^a^ Unless otherwise stated, reactions were carried out on a 0.5 mmol scale in DMF at 80 °C for 24 h using 1 equiv of aryl iodide **1**, 2 equiv of KOAc, 0.025 equiv of Pd_2_(dba)_3_, and 3 equiv of LiCl using a HCOOLi/Ac_2_O combination [HCOOLi·H_2_O (3.358 g, 48.0 mmol), acetic anhydride (3.024 mL, 32.0 mmol) in anhydrous DMF (20 mL)] as a condensed external source of carbon monoxide. ^b^ Yields are given for isolated products. ^c^ In the absence of LiCl. ^d^ In the presence of 0.025 equiv of Pd_2_(dba)_3_, 3 equiv of lithium chloride in DMF, and 10 equiv of water. The carboxylic acid was isolated in 50% yield as the acetyl derivative using the potassium acetate protocol.

**Table 2:** Preparation of α,β-unsaturated carboxylic acids via parallel palladium-catalyzed hydroxycarbonylation of vinyl triflates using the HCOOLi/Ac_2_O combination as external condensed source of carbon monoxide ^a^.

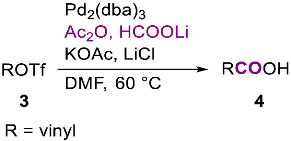					
Entry	Vinyl Triflate 3		α,β-Unsaturated Acid 3		Yield % ^b^
1	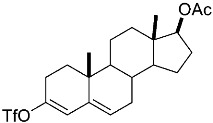	**3a**	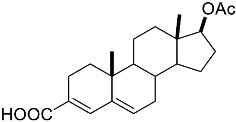	**4a**	71
2	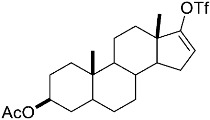	**3b**	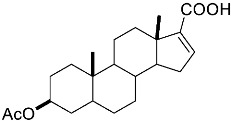	**4b**	81
3	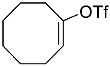	**3c**	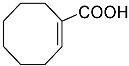	**4c**	71
4	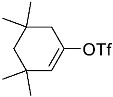	**3d**	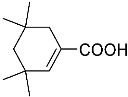	**4d**	86
5	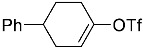	**3e**	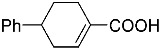	**4e**	82
6	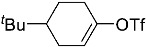	**3f**	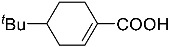	**4f**	87
7	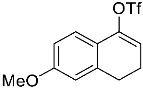	**3g**	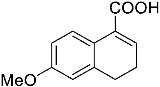	**4g**	82

^a^ Reactions were carried out on a 0.5 mmol scale in DMF at 60 °C for 24 h using 1 equiv of vinyl triflate **3**, 2 equiv of KOAc, 0.025 equiv of Pd_2_(dba)_3_, and 3 equiv of LiCl using a HCOOLi/Ac_2_O combination [HCOOLi·H_2_O (2.938 g, 42.0 mmol), acetic anhydride (2.644 mL, 28.0 mmol) in anhydrous DMF (18 mL)] as a condensed external source of carbon monoxide. ^b^ Yields are given for isolated products.

**Table 3:** Preparation of benzoic acids via parallel palladium-catalyzed hydroxycarbonylation of aryl bromides using the HCOOLi/Ac_2_O combination as external condensed source of carbon monoxide ^a^.

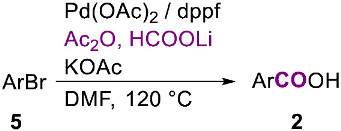					
Entry	Aryl Bromide 5		Benzoic Acid 2		Yield % ^b^
1	C_6_H_5_-Br	**5a**	C_6_H_5_-COOH	**2i**	71
2	*p*-Me-C_6_H_4_-Br	**5b**	*p*-Me-C_6_H_4_-COOH	**2b**	78
3	*m*-MeO-C_6_H_4_-Br	**5c**	*m*-MeO-C_6_H_4_-COOH	**2c**	72
4	*p*-MeO-C_6_H_4_-Br	**5d**	*p*-MeO-C_6_H_4_-COOH	**2d**	69 ^c^
5	*p*-Ph-C_6_H_4_-Br	**5e**	*p*-Ph-C_6_H_4_-COOH	**2j**	70
6	*p*-NO_2_-C_6_H_4_-Br	**5f**	*p*-NO_2_-C_6_H_4_-COOH	**2k**	75
7	*p*-EtOOC-C_6_H_4_-Br	**5g**	*p*-EtOOC-C_6_H_4_-COOH	**2a**	71
8	*p*-MeCO-C_6_H_4_-Br	**5h**	*p*-MeCO-C_6_H_4_-COOH	**2h**	70

^a^ Unless otherwise stated, reactions were carried out on a 0.5 mmol scale in DMF at 120 °C for 24 h using 1 equiv of aryl bromide **5**, 0.05 equiv of Pd(OAc)_2_, 0.05 equiv of dppf, 2 equiv of KOAc, 3 equiv of LiCl using a HCOOLi/Ac_2_O combination [HCOOLi·H_2_O (3.358 g, 48.0 mmol), acetic anhydride (3.024 mL, 32.0 mmol) in anhydrous DMF (20 mL)] as a condensed external source of carbon monoxide. ^b^ Yields are given for isolated products. ^c^ In the absence of LiCl.

## Data Availability

Data contained within the article or Supplementary Materials.

## Supplementary Materials

The following supporting information can be downloaded at https://www.mdpi.com/article/10.3390/molecules30153298/s1: ^1^H, ^13^C, DEPT 135, ^19^F NMR Spectra of final compounds and prepared vinyl triflates.
