# Supplementary material for: Parallel Palladium-Catalyzed Synthesis of Carboxylic Acids from Aryl Iodides, Bromides, and Vinyl Triflates Using Acetic Anhydride and Formate Anion as an External Condensed Source of Carbon Monoxide
Source: Molecules. 2025 Aug 6;30(15):3298. doi: 10.3390/molecules30153298 (PMC12348999; doi:10.3390/molecules30153298)

^{1}H, ^{13}C, DEPT 135, ^{19}F NMR Spectra

^{1}H NMR-spectrum (400 MHz, DMSO-d_6)

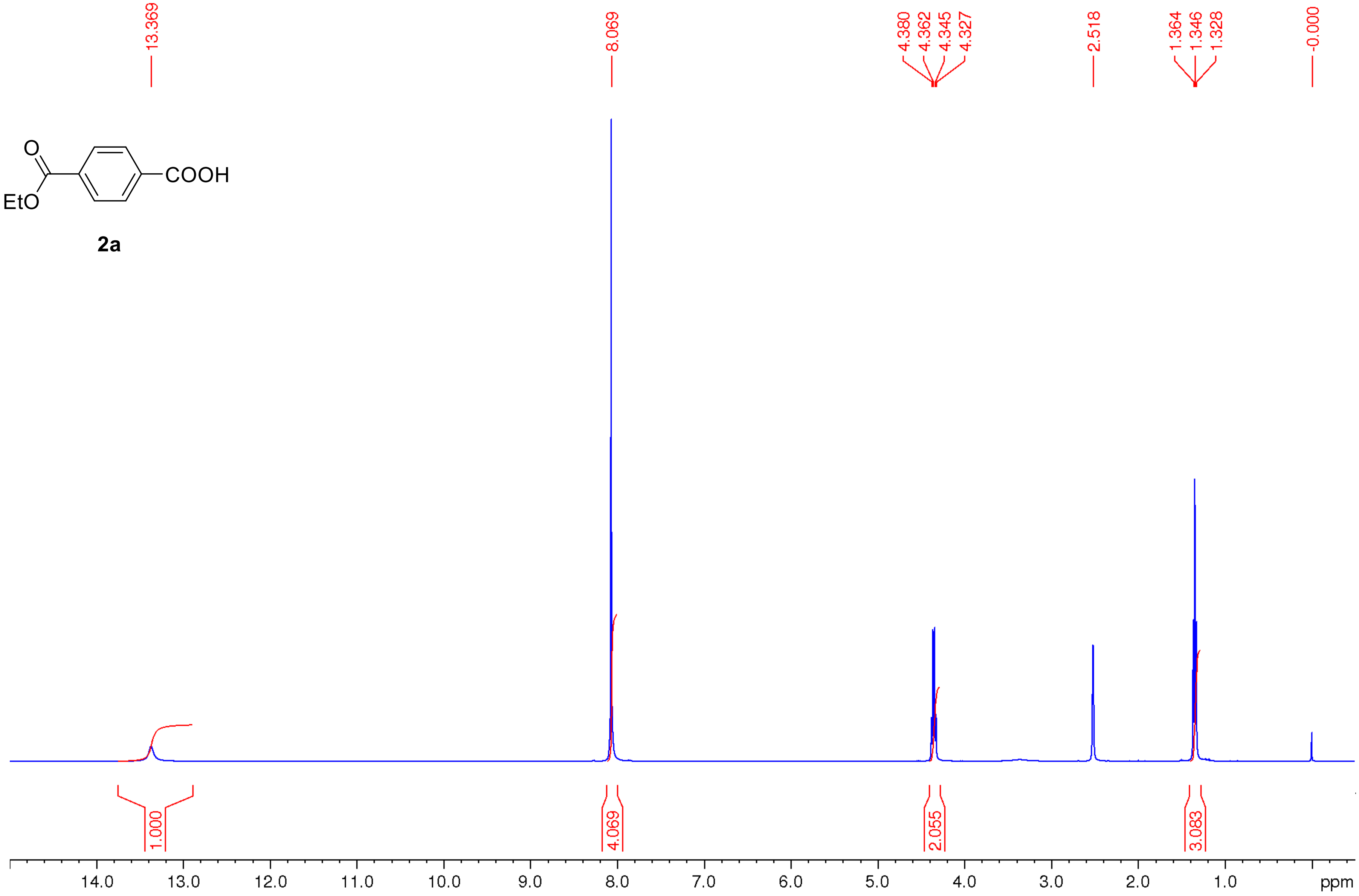

2a

^{13}C NMR-spectrum (100 MHz, DMSO-d_6)

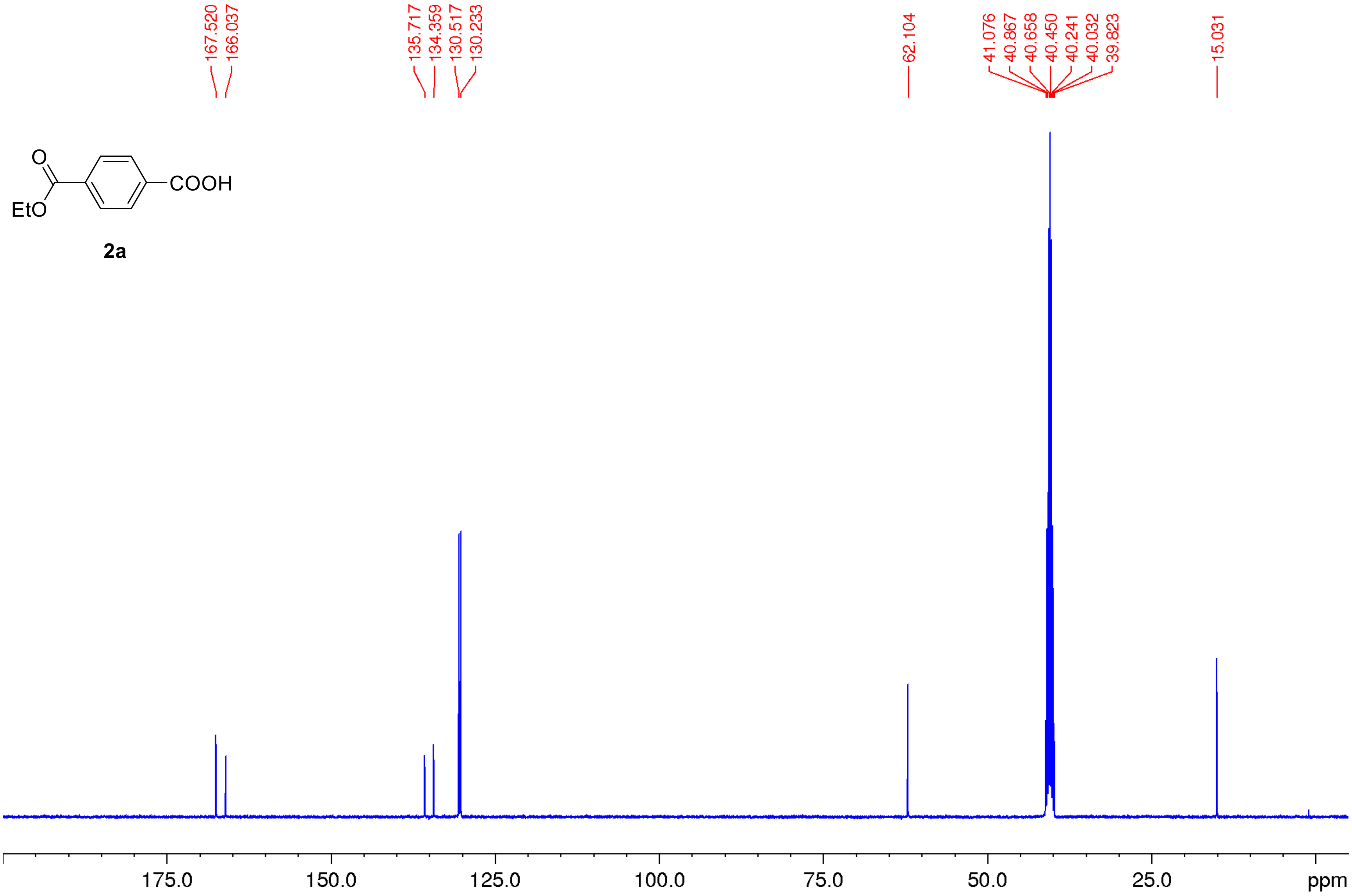

DEPT 135 NMR-spectrum (DMSO-d_6)

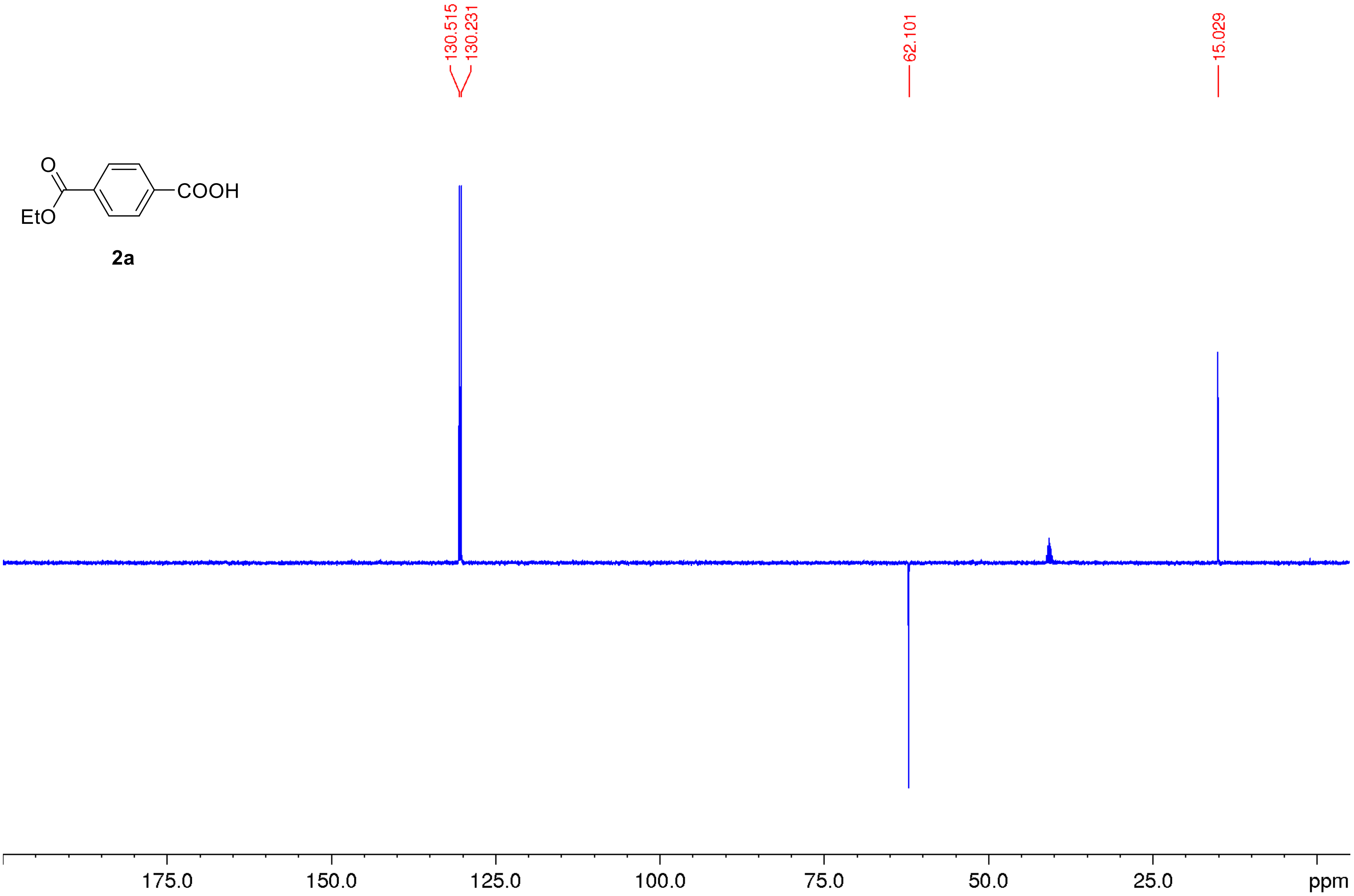

^{1}H NMR-spectrum (400 MHz, DMSO-d_6)

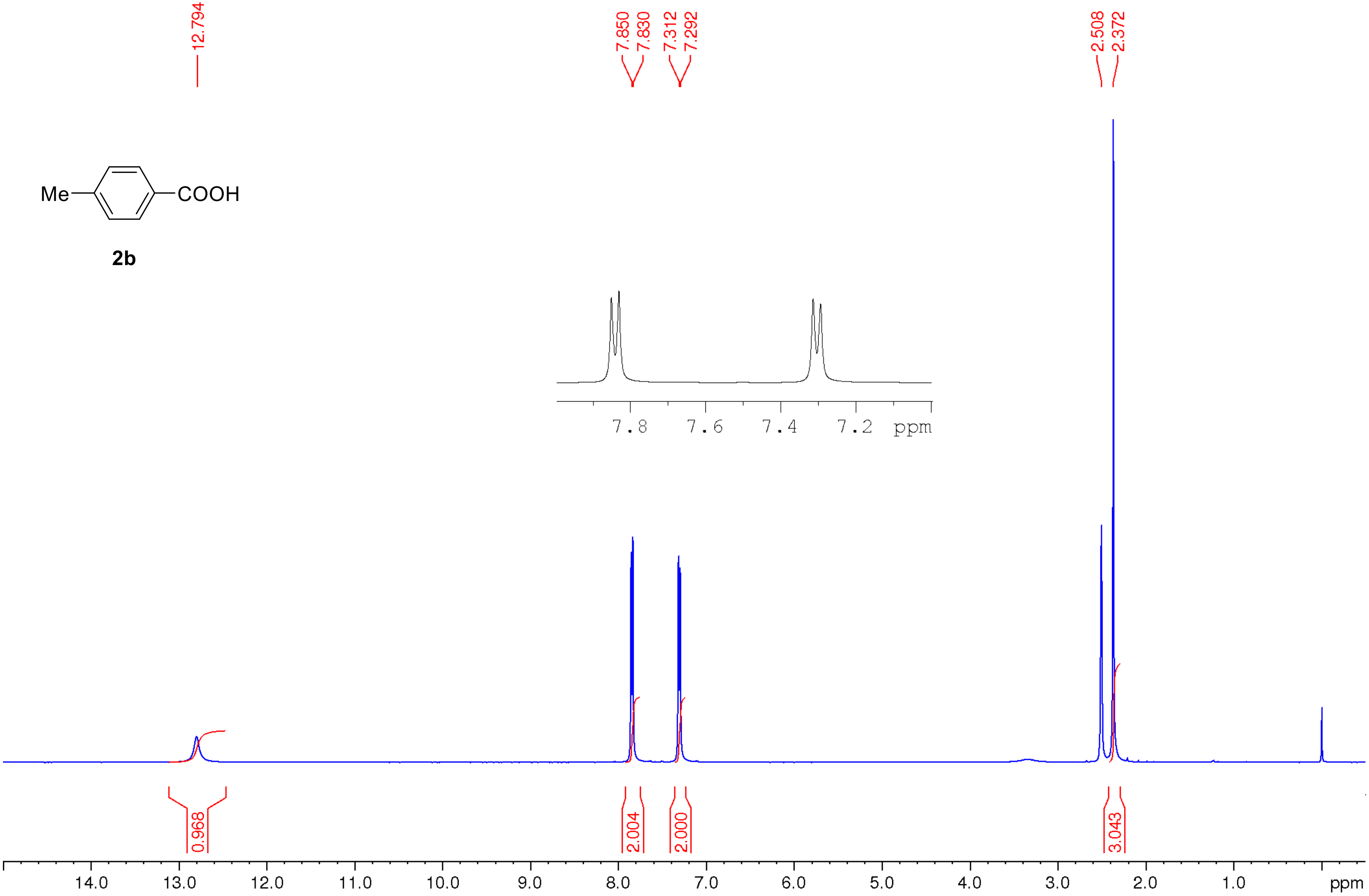

2b

^{13}C NMR-spectrum (100 MHz, DMSO-d_6)

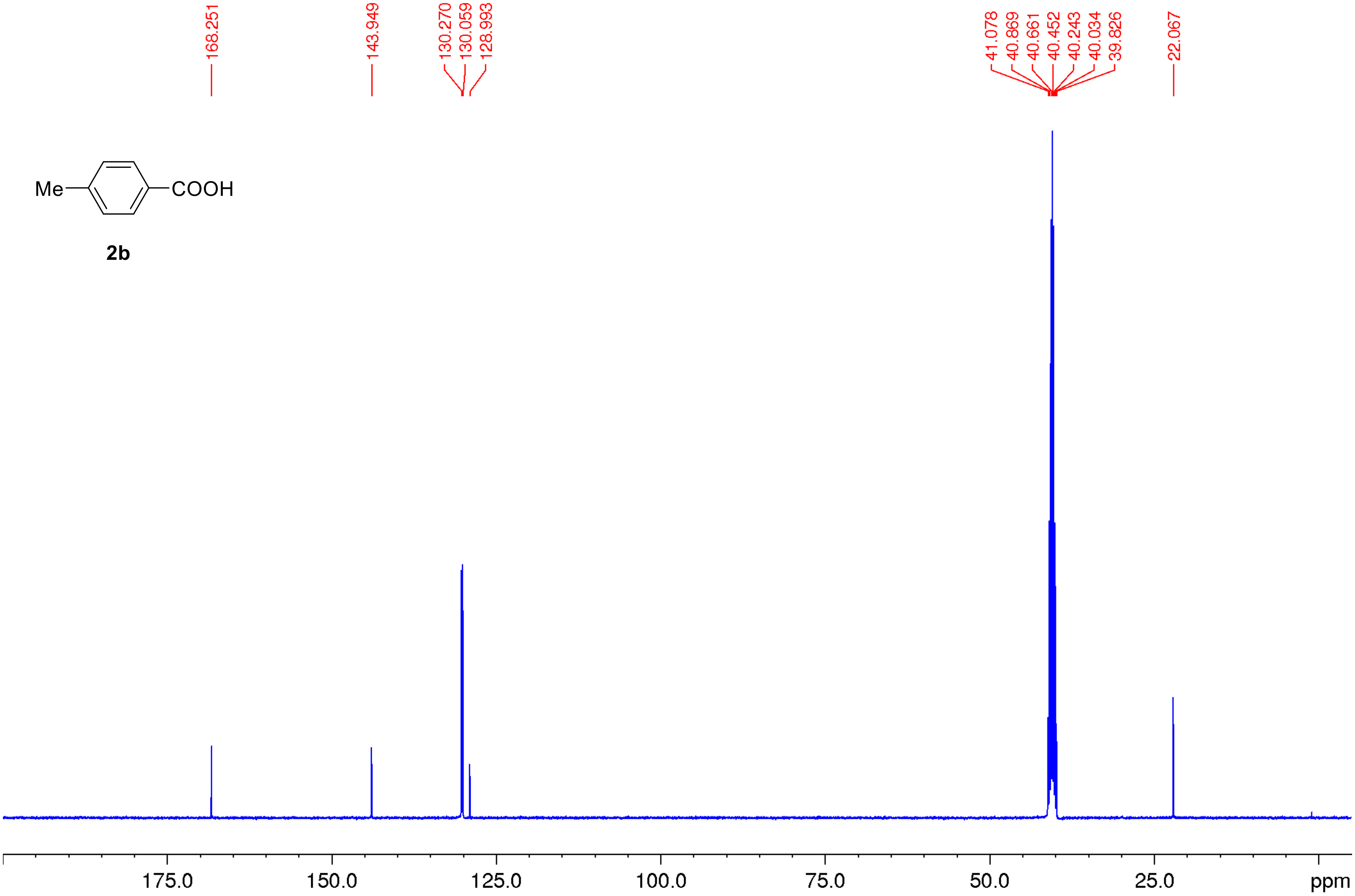

DEPT 135 NMR-spectrum (DMSO-d_6)

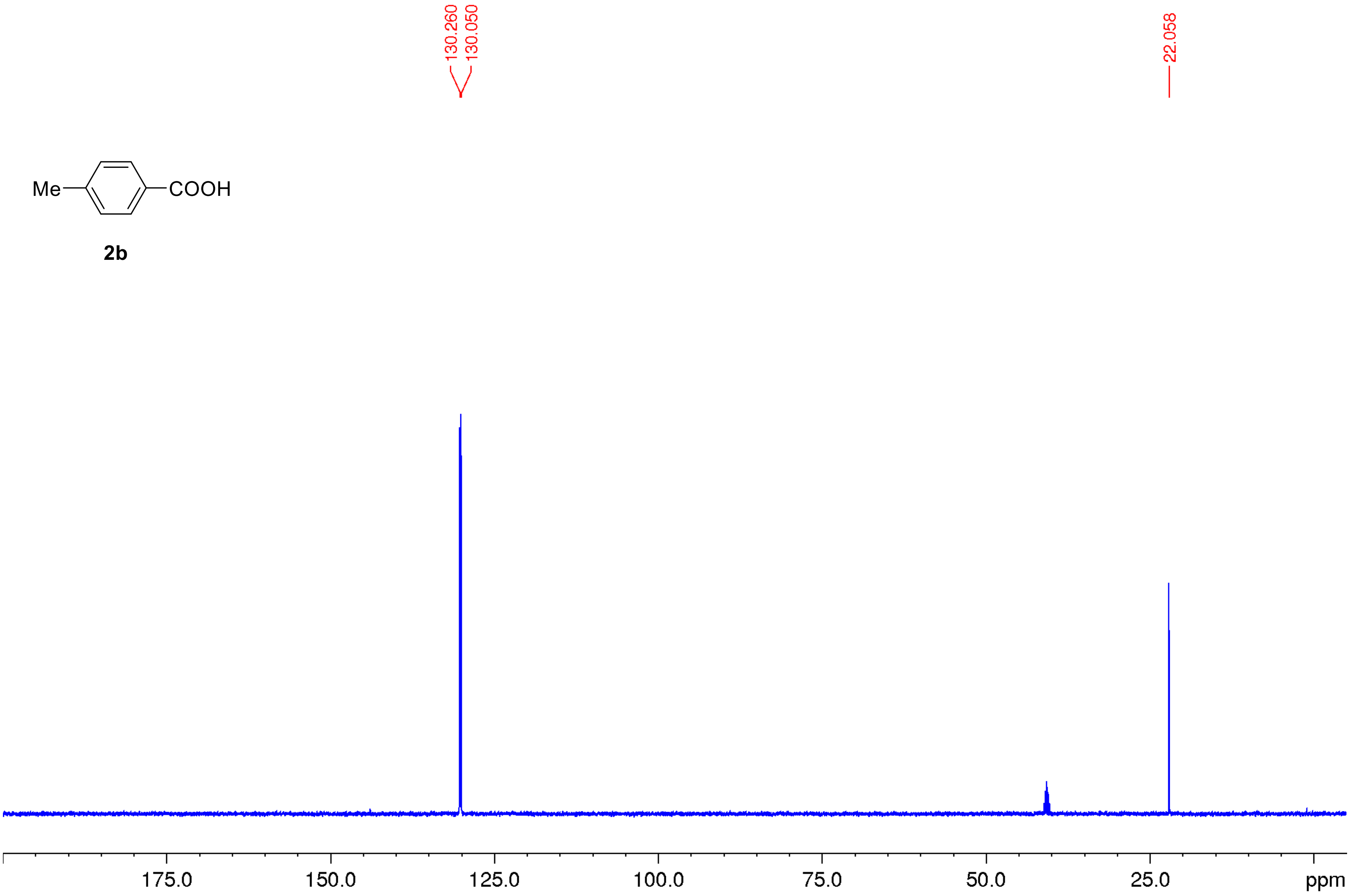

^{1}H NMR-spectrum (400 MHz, DMSO-d_6)

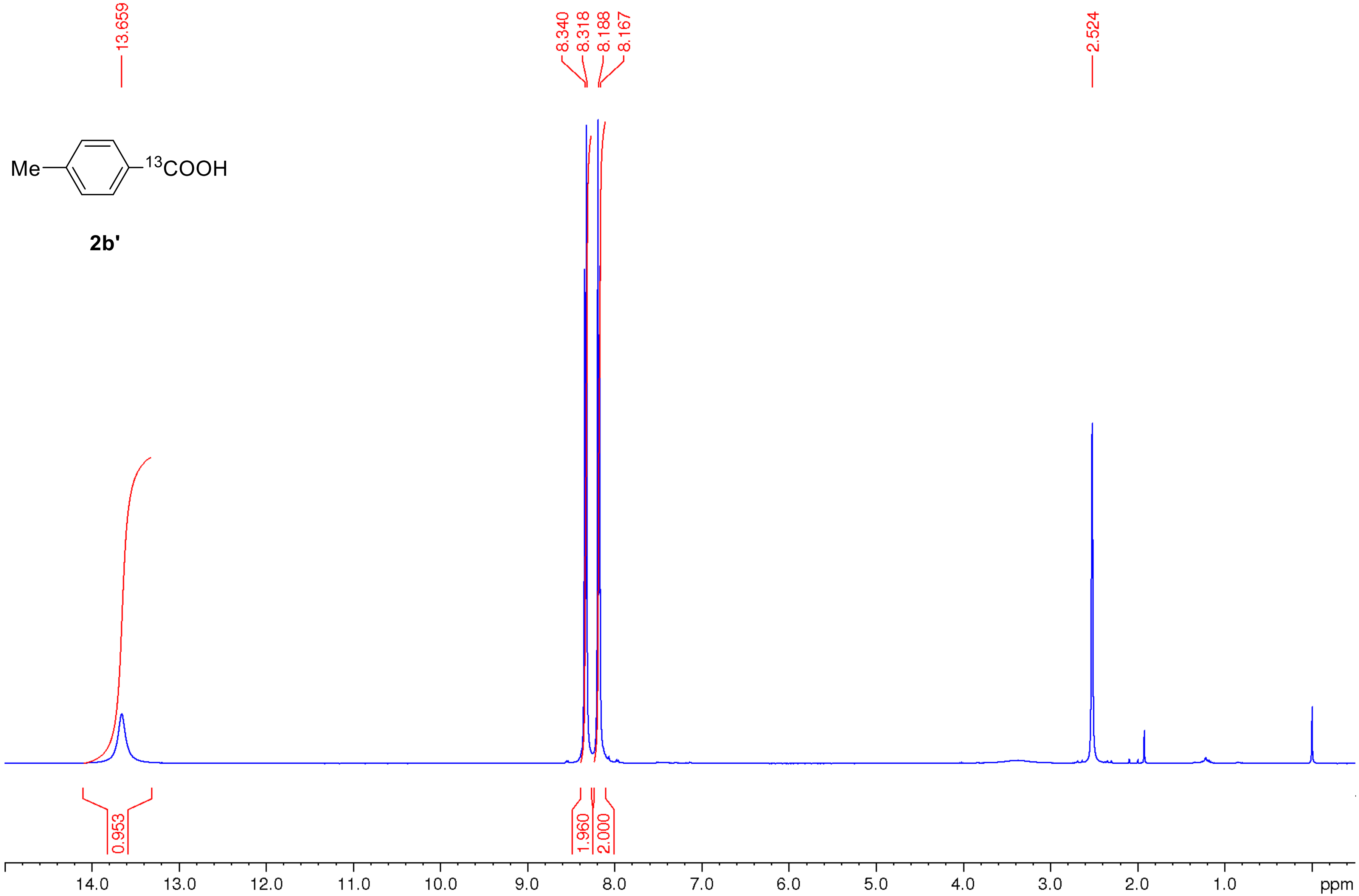

^{13}C NMR-spectrum (100 MHz, DMSO-d_6)

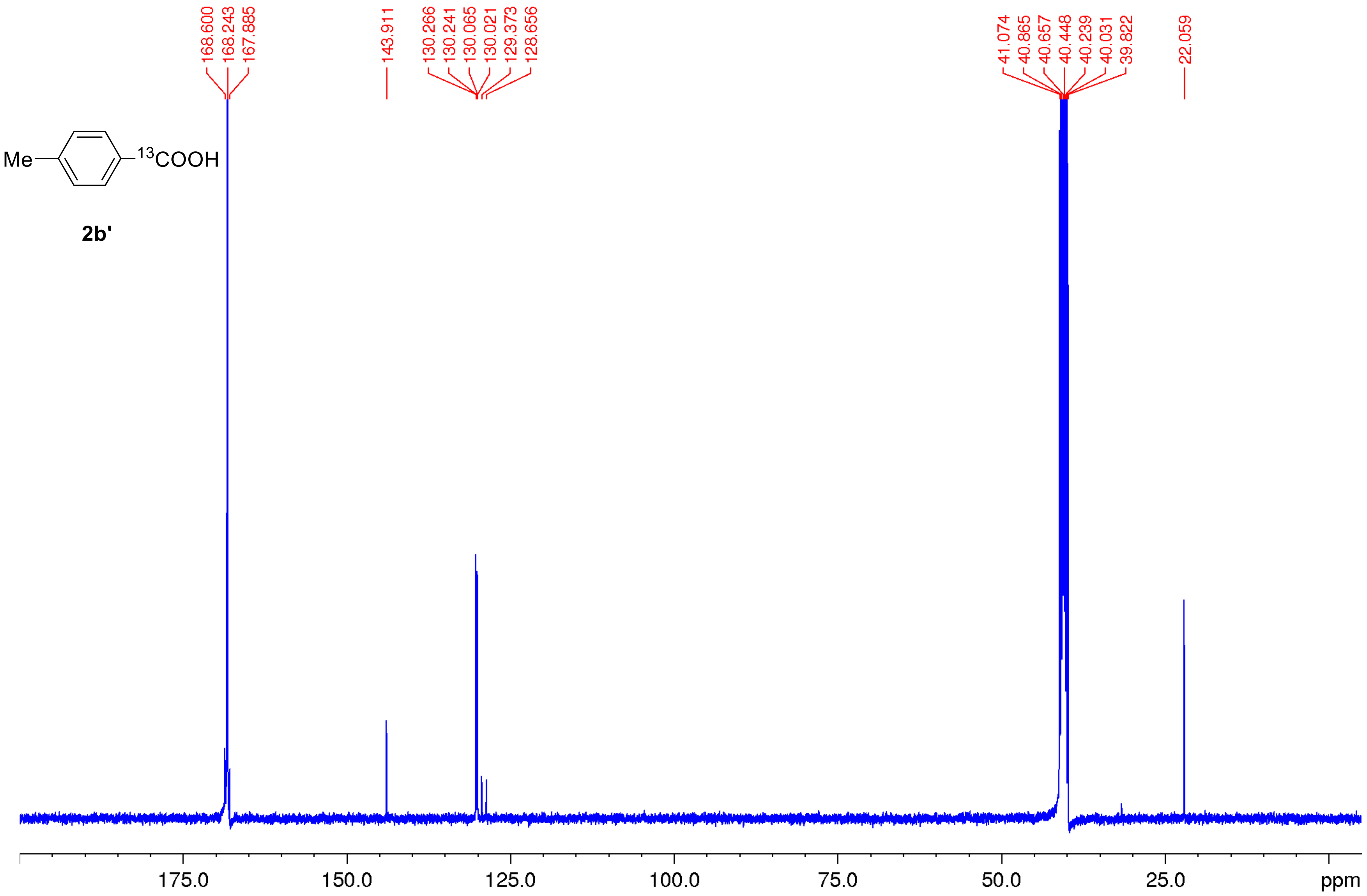

2b'

DEPT 135 NMR-spectrum (DMSO-d_6)

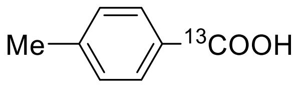

2b'

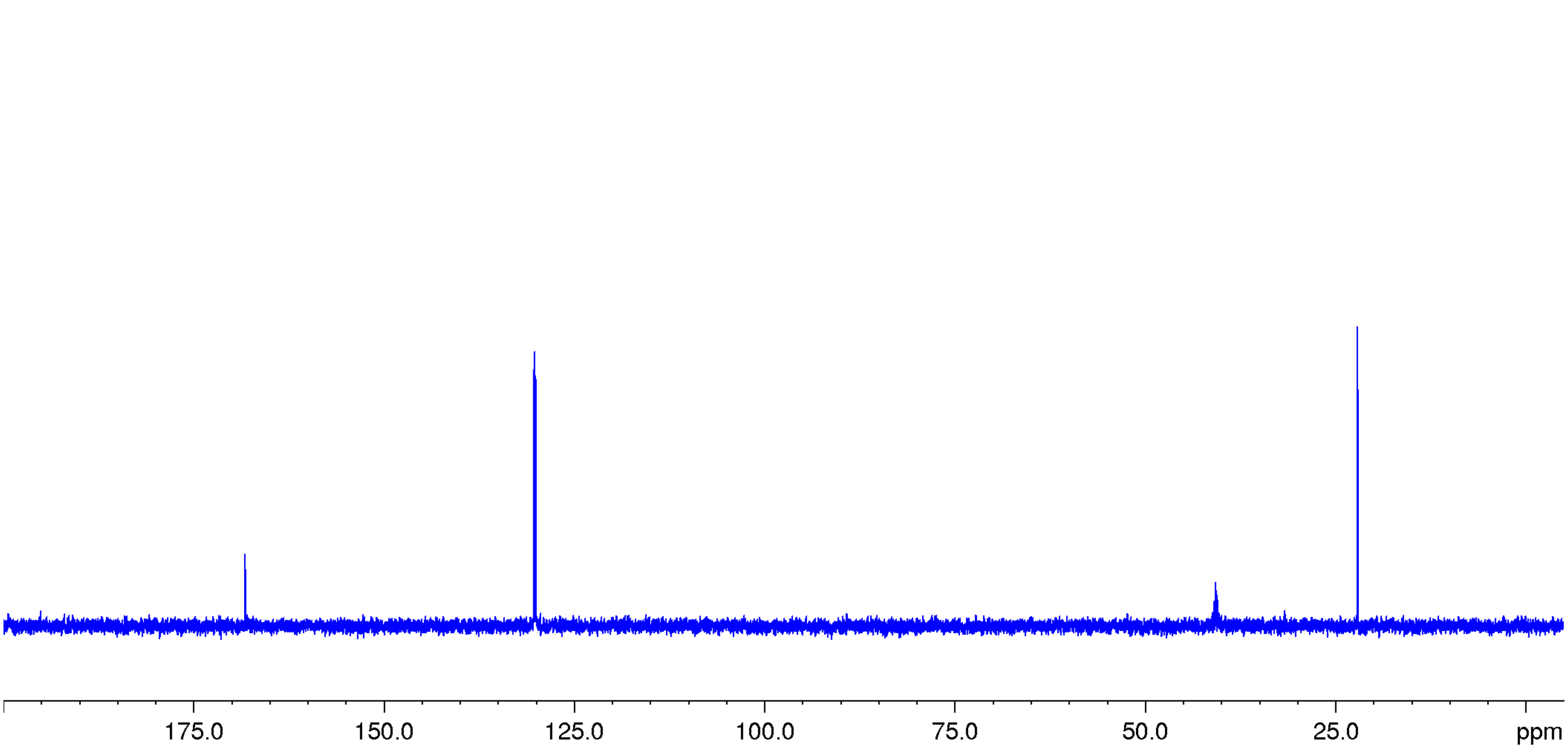

^{1}H NMR-spectrum (400 MHz, DMSO-d_6)

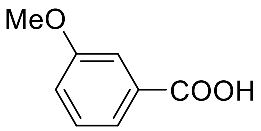

2c

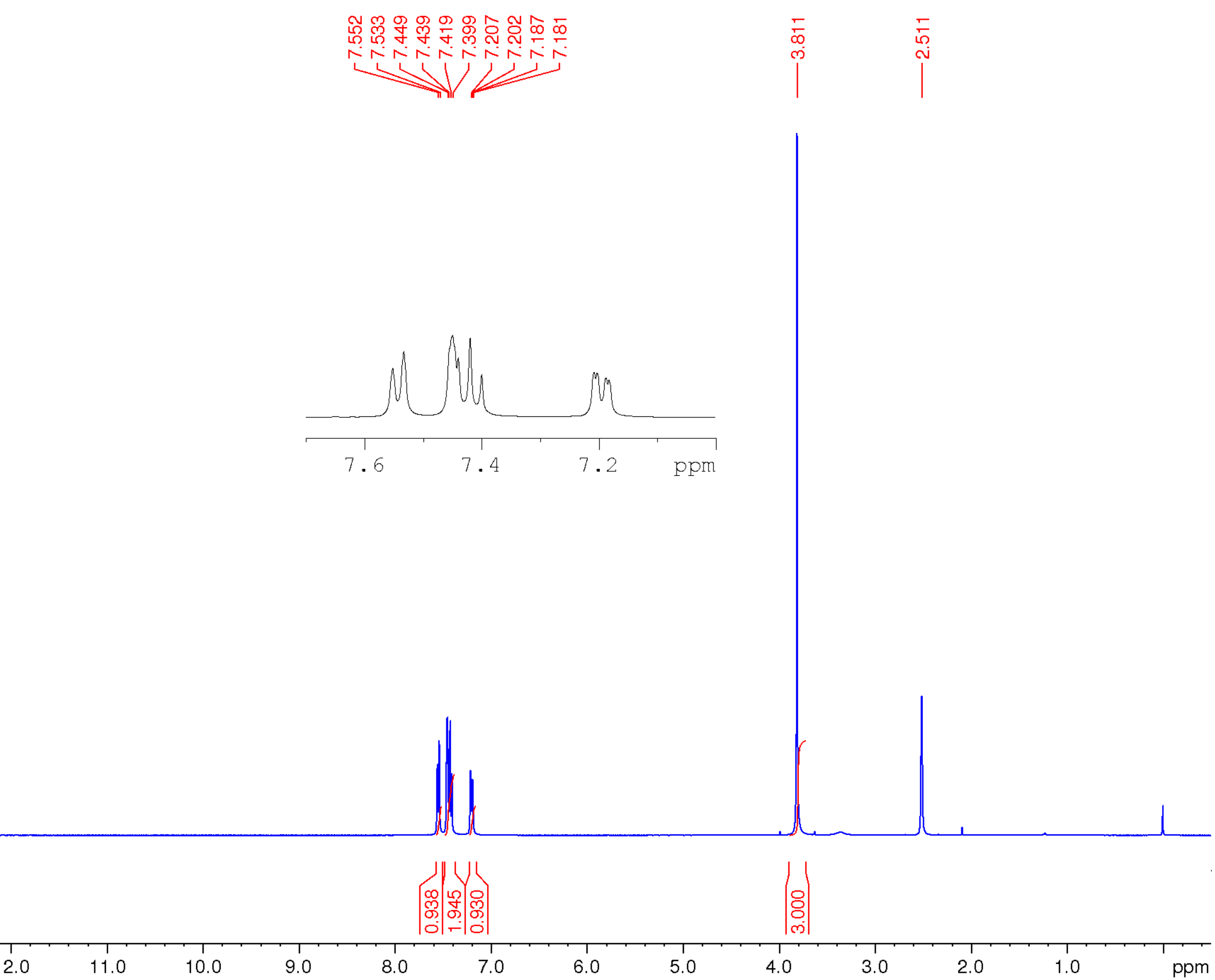

^{13}C NMR-spectrum (100 MHz, DMSO-d_6)

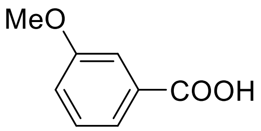

2c

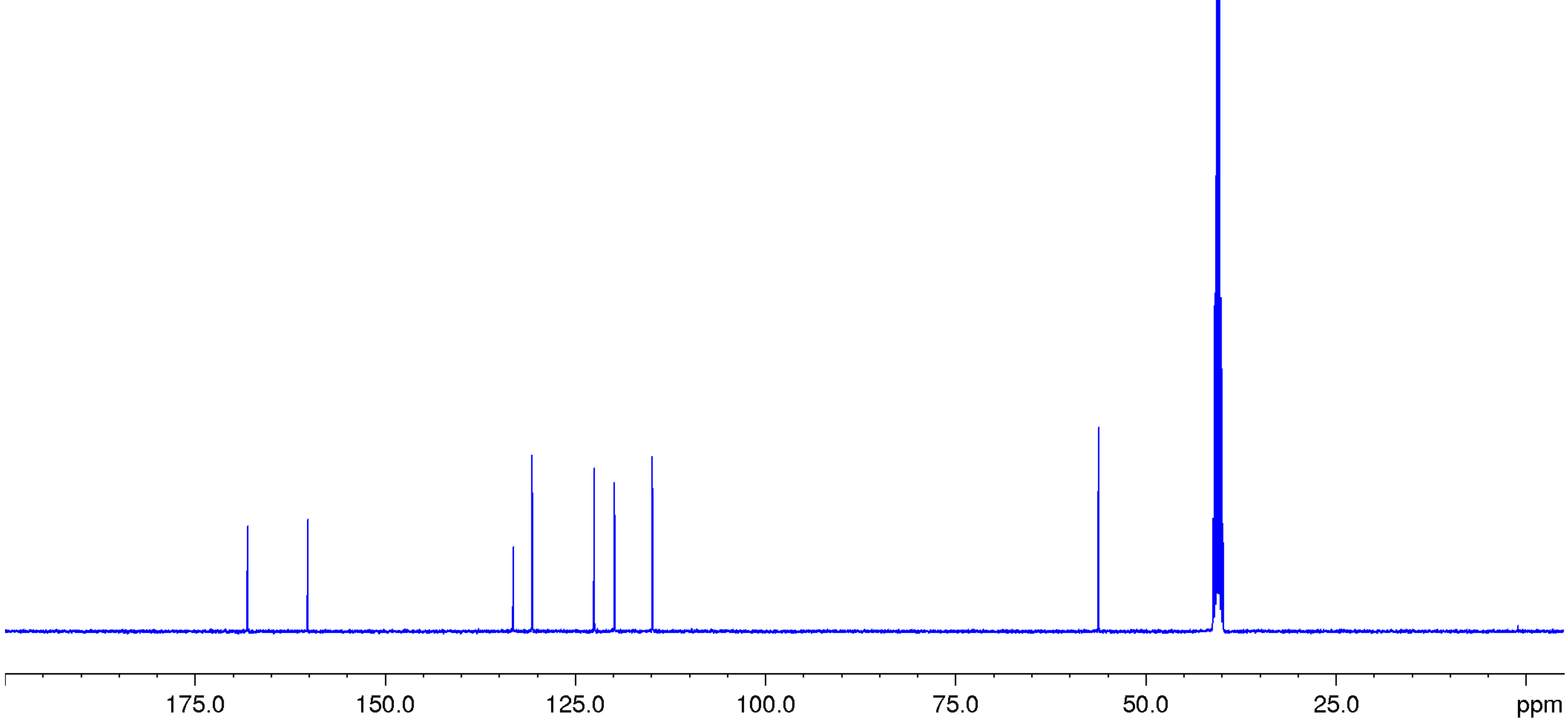

DEPT 135 NMR-spectrum (DMSO-d_6)

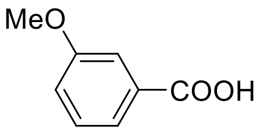

2c

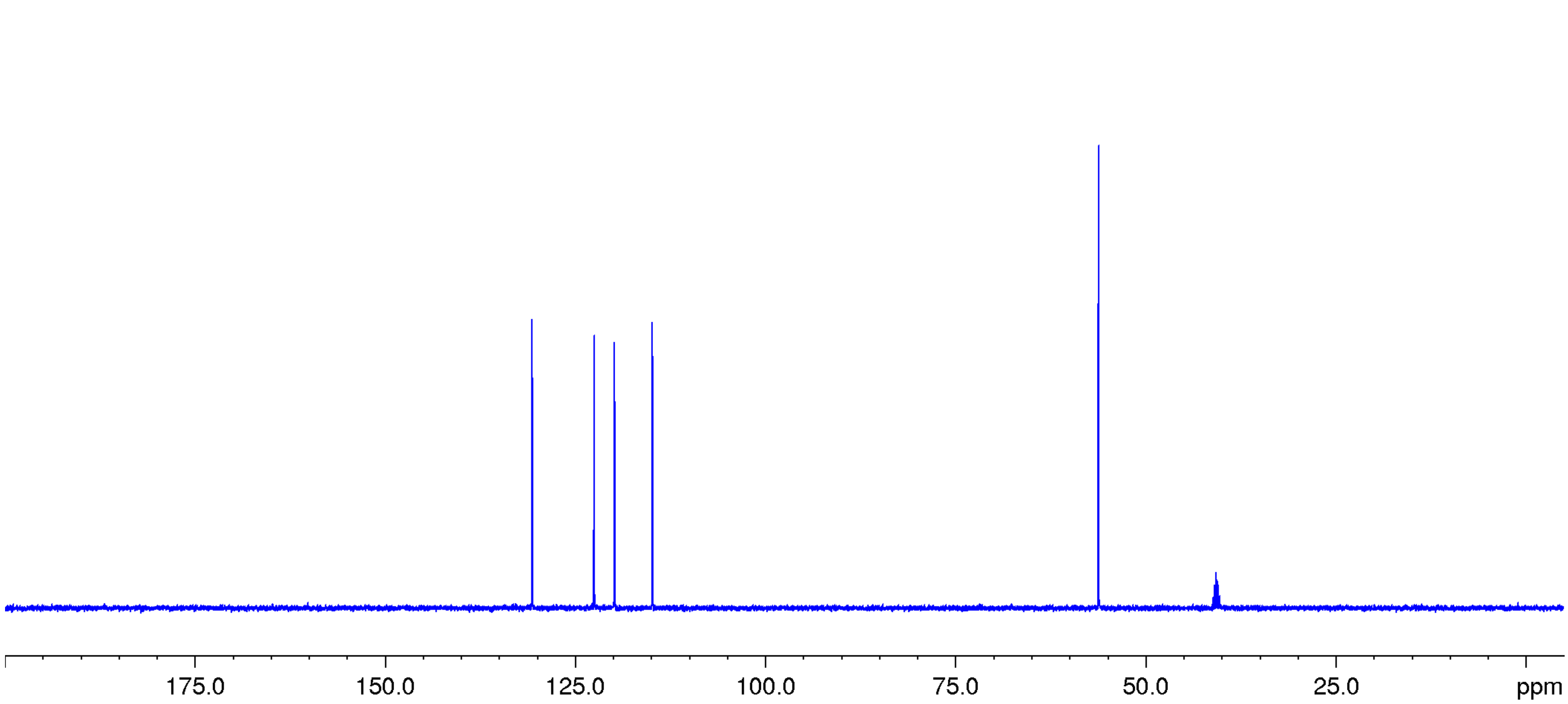

^{1}H NMR-spectrum (400 MHz, DMSO-d_6)

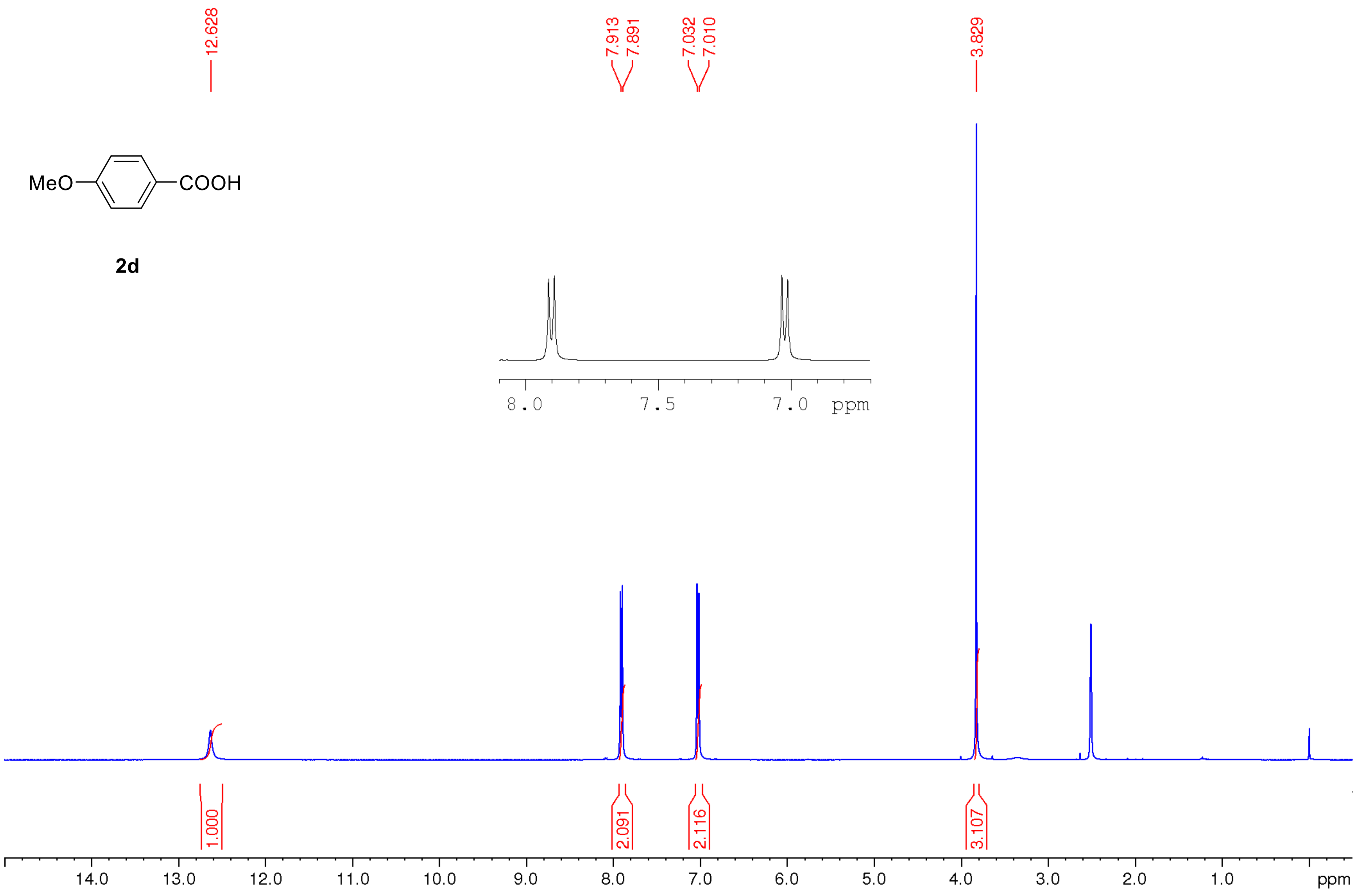

2d

^{13}C NMR-spectrum (100 MHz, DMSO-d_6)

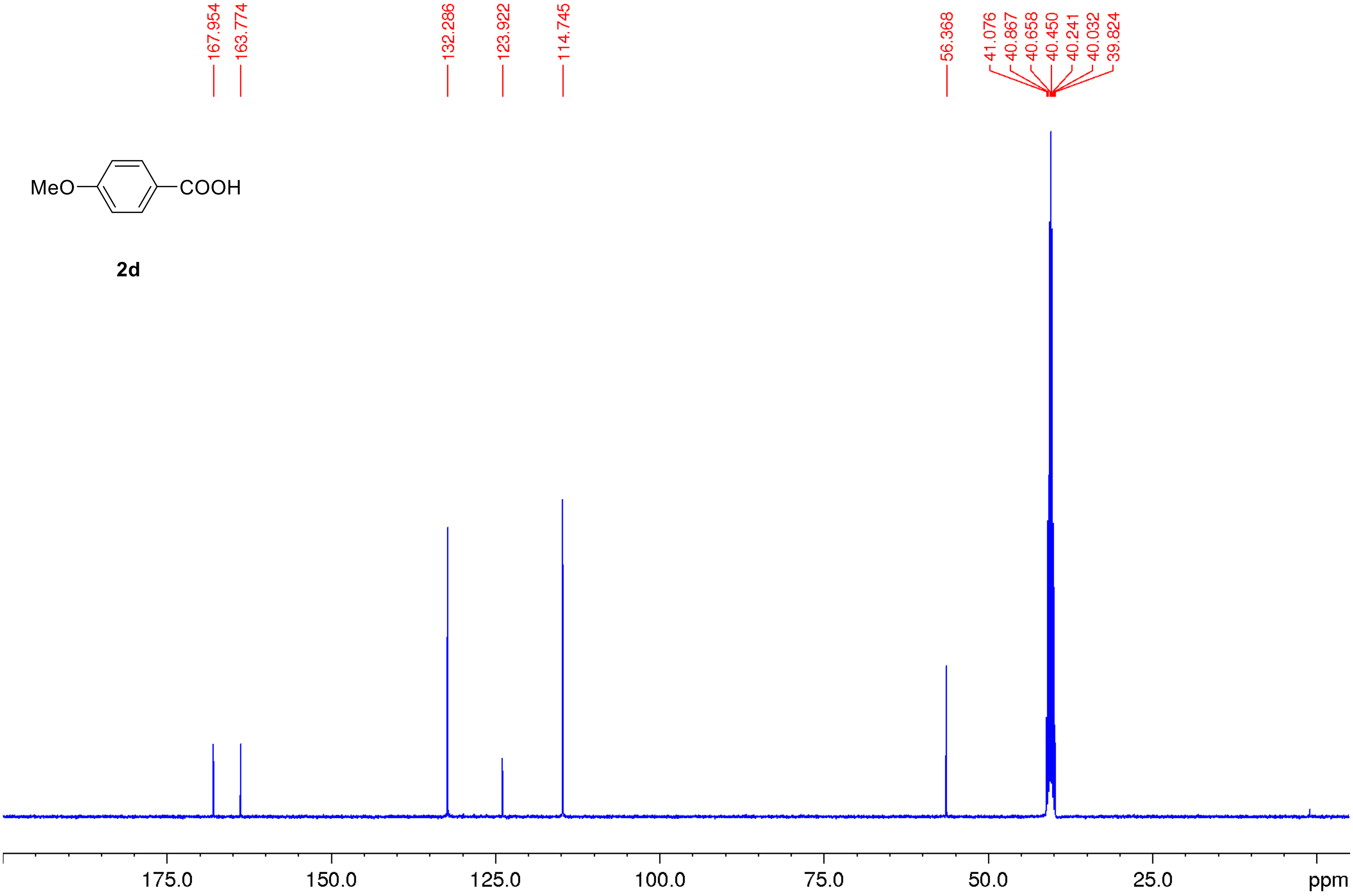

2d

DEPT 135 NMR-spectrum (DMSO-d_6)

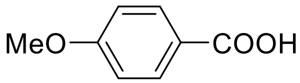

2d

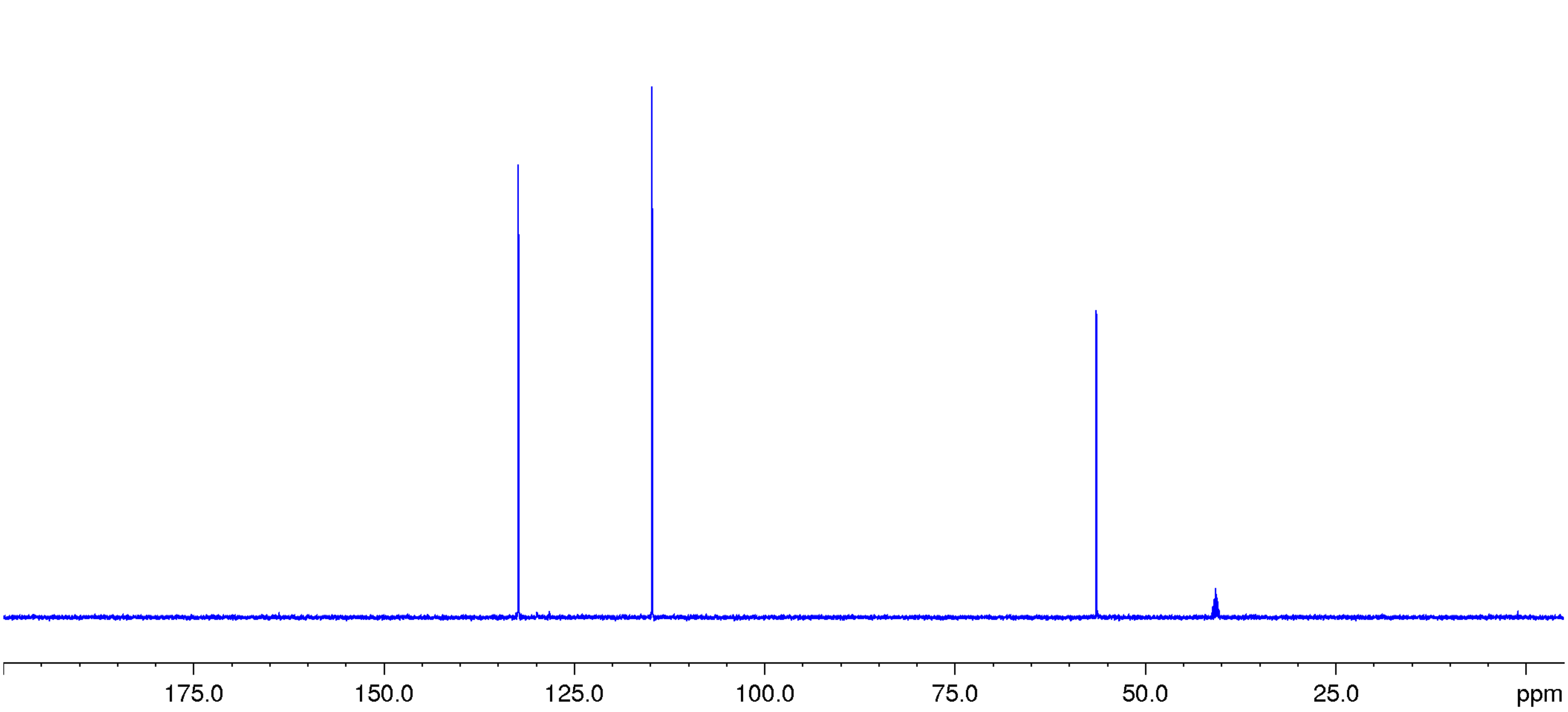

^{1}H NMR-spectrum (400 MHz, DMSO-d_6)

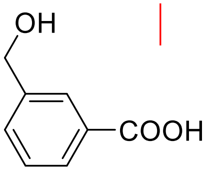

2e

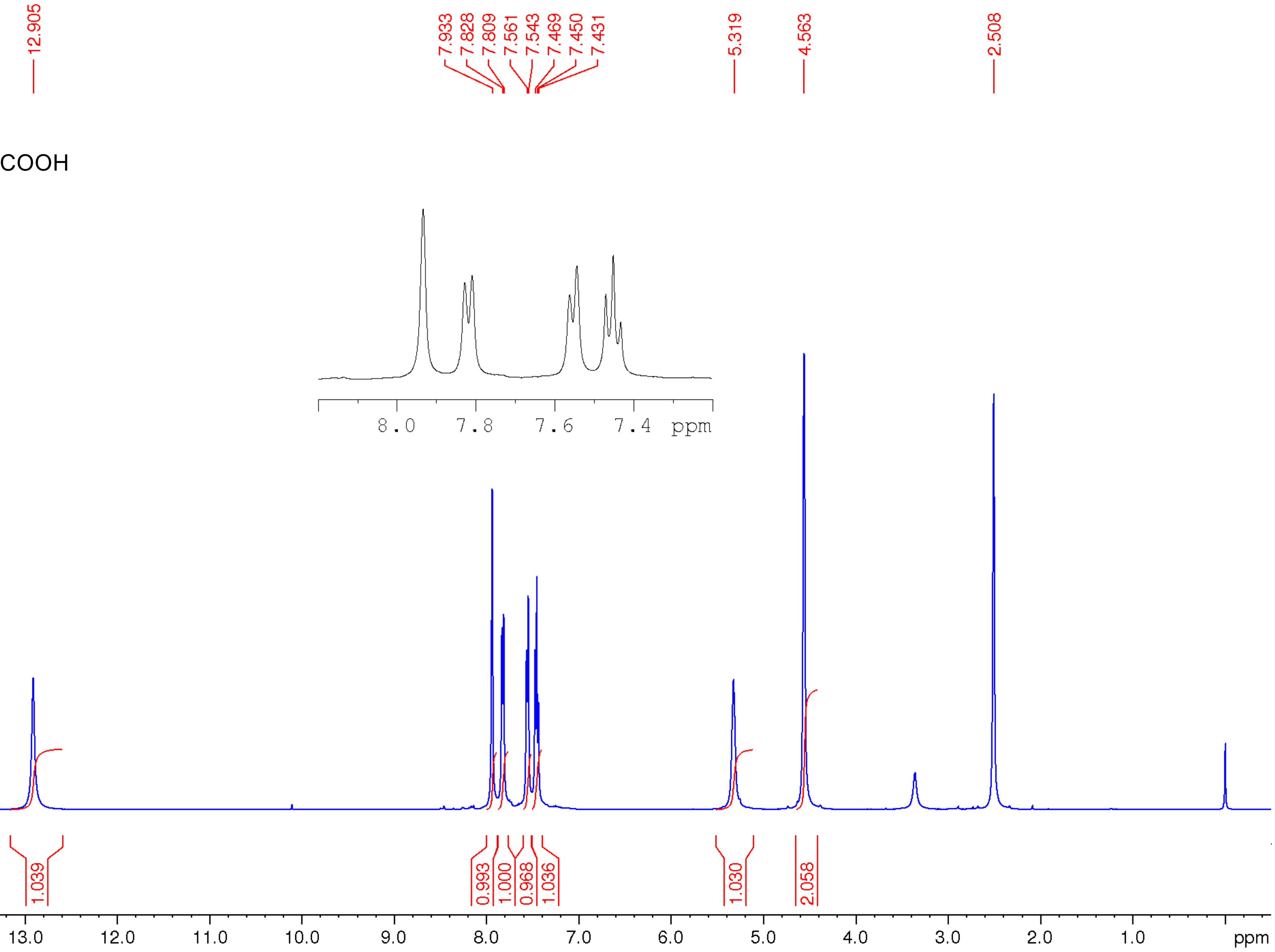

^{13}C NMR-spectrum (100 MHz, DMSO-d_6)

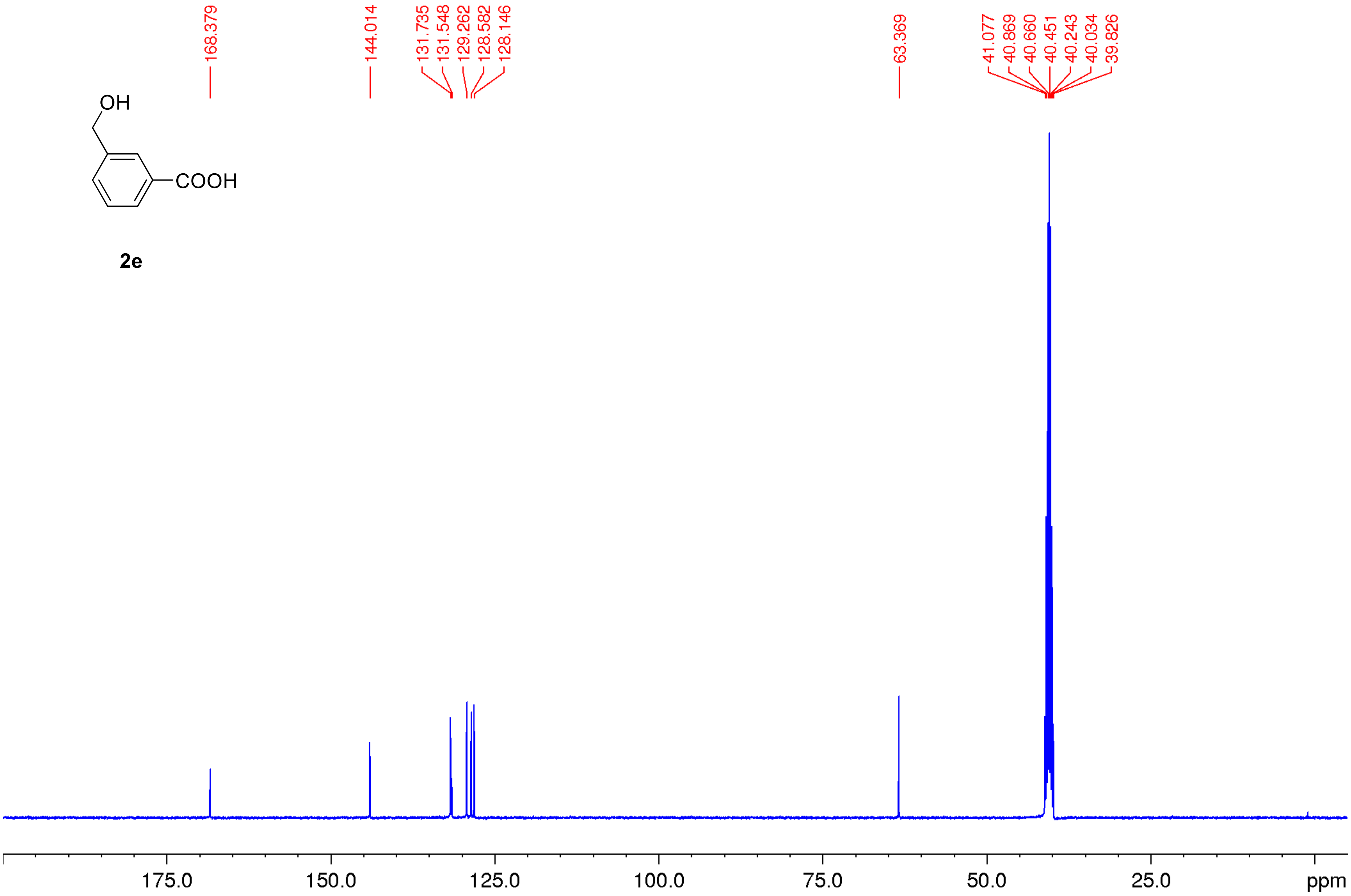

DEPT 135 NMR-spectrum (DMSO-d_6)

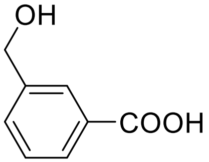

2e

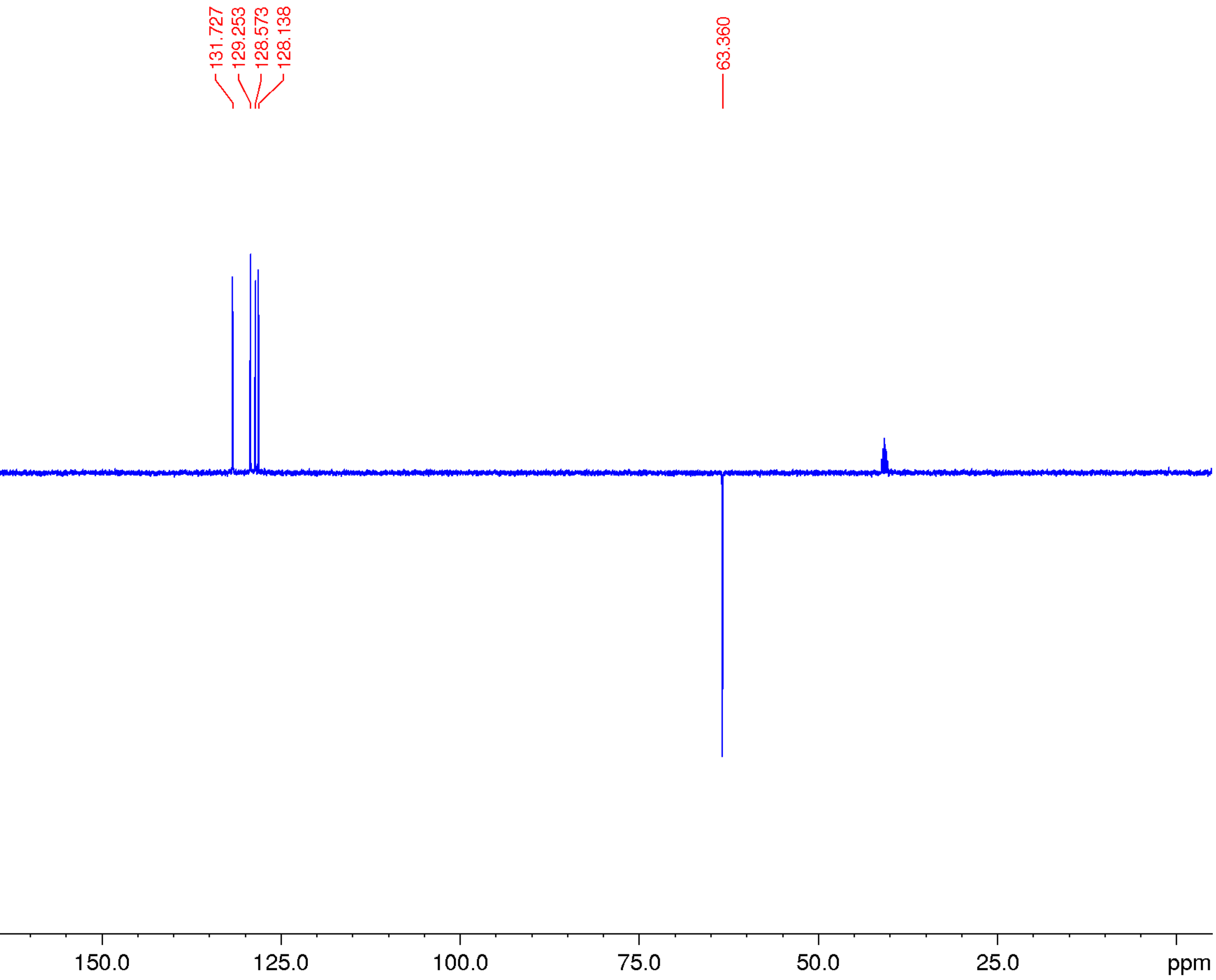

^{1}H NMR-spectrum (400 MHz, DMSO-d_6)

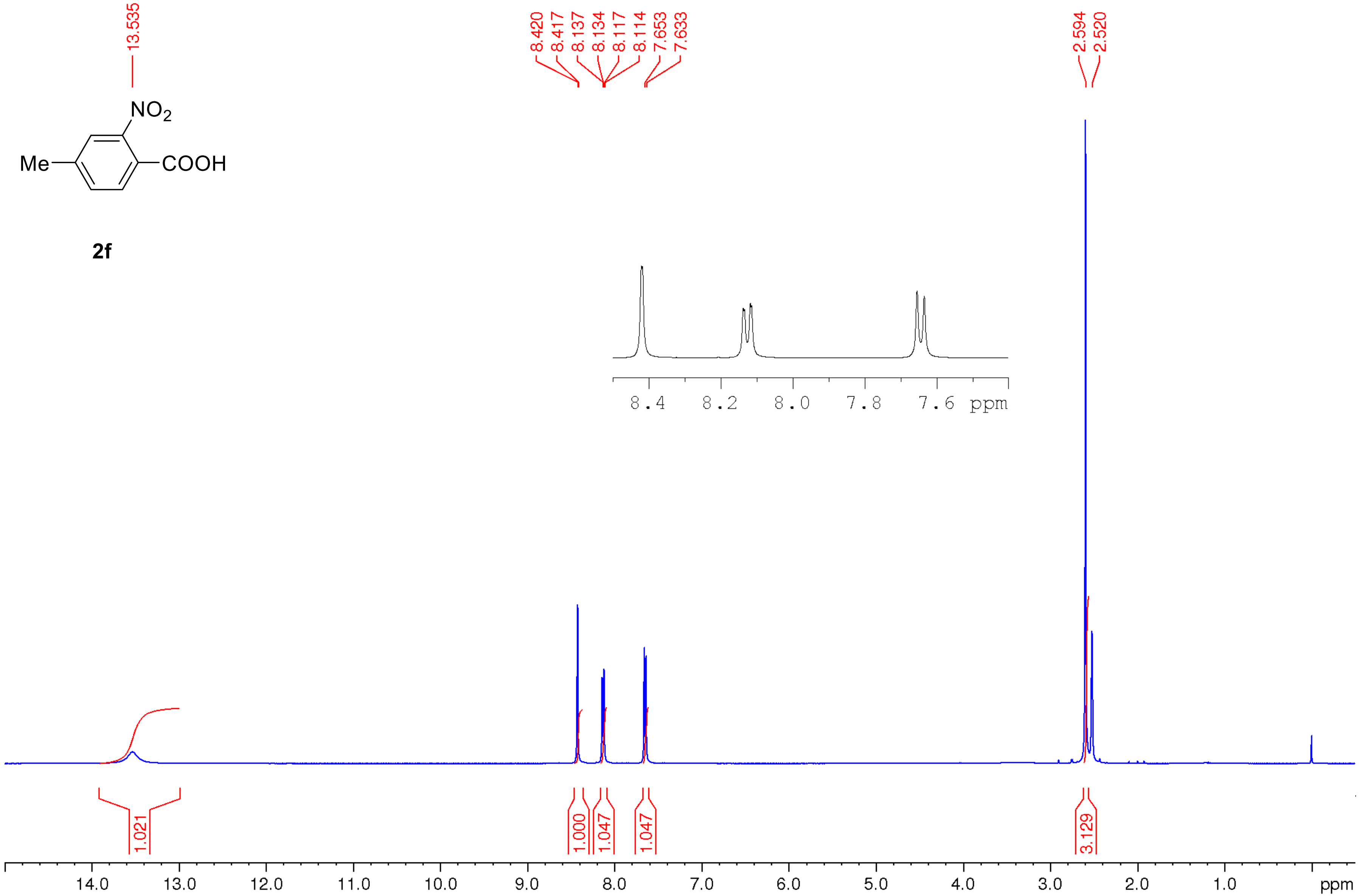

^{13}C NMR-spectrum (100 MHz, DMSO-d_6)

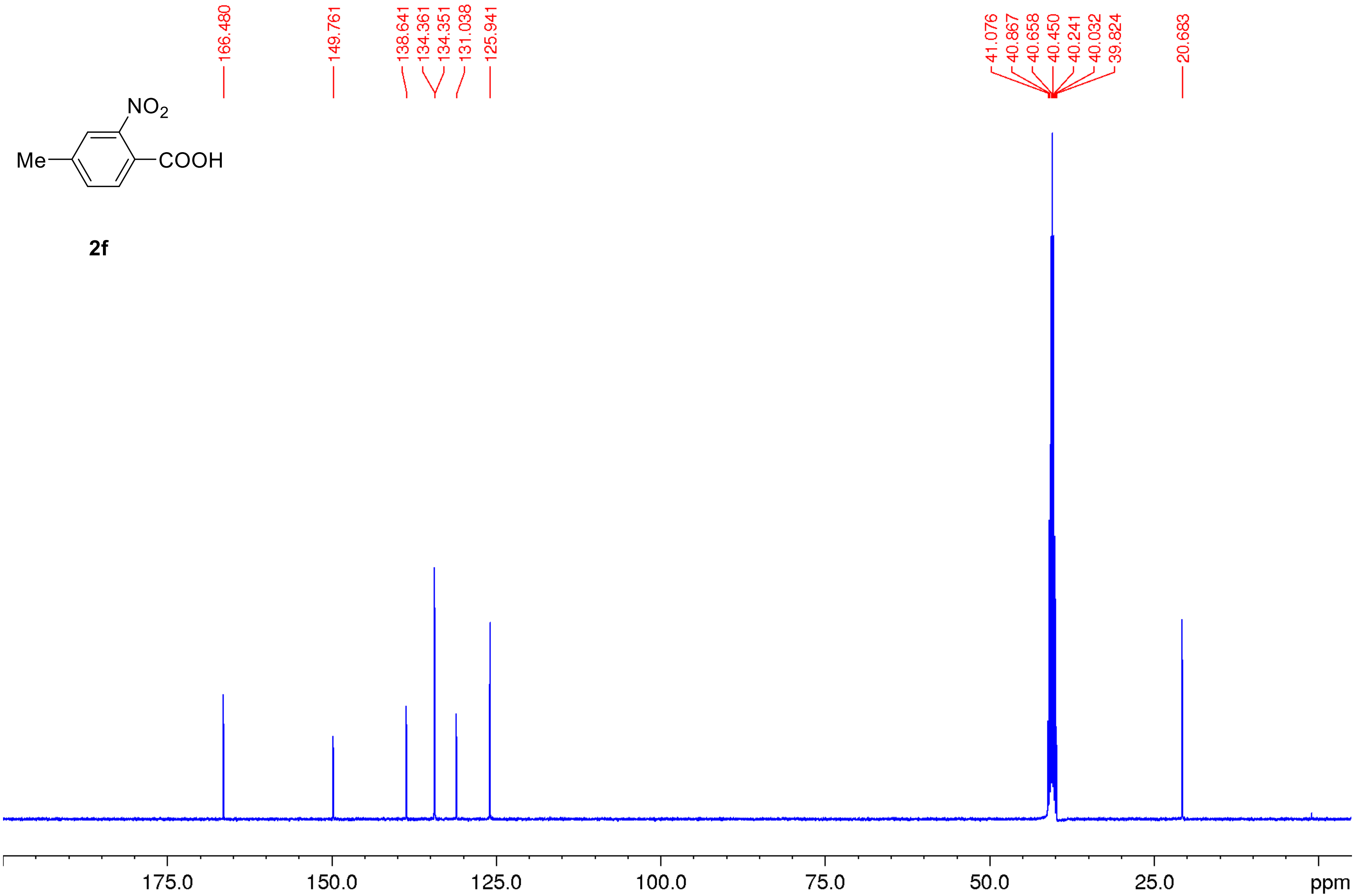

DEPT 135 NMR-spectrum (DMSO-d_6)

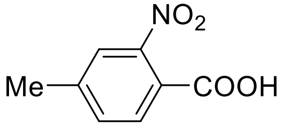

2f

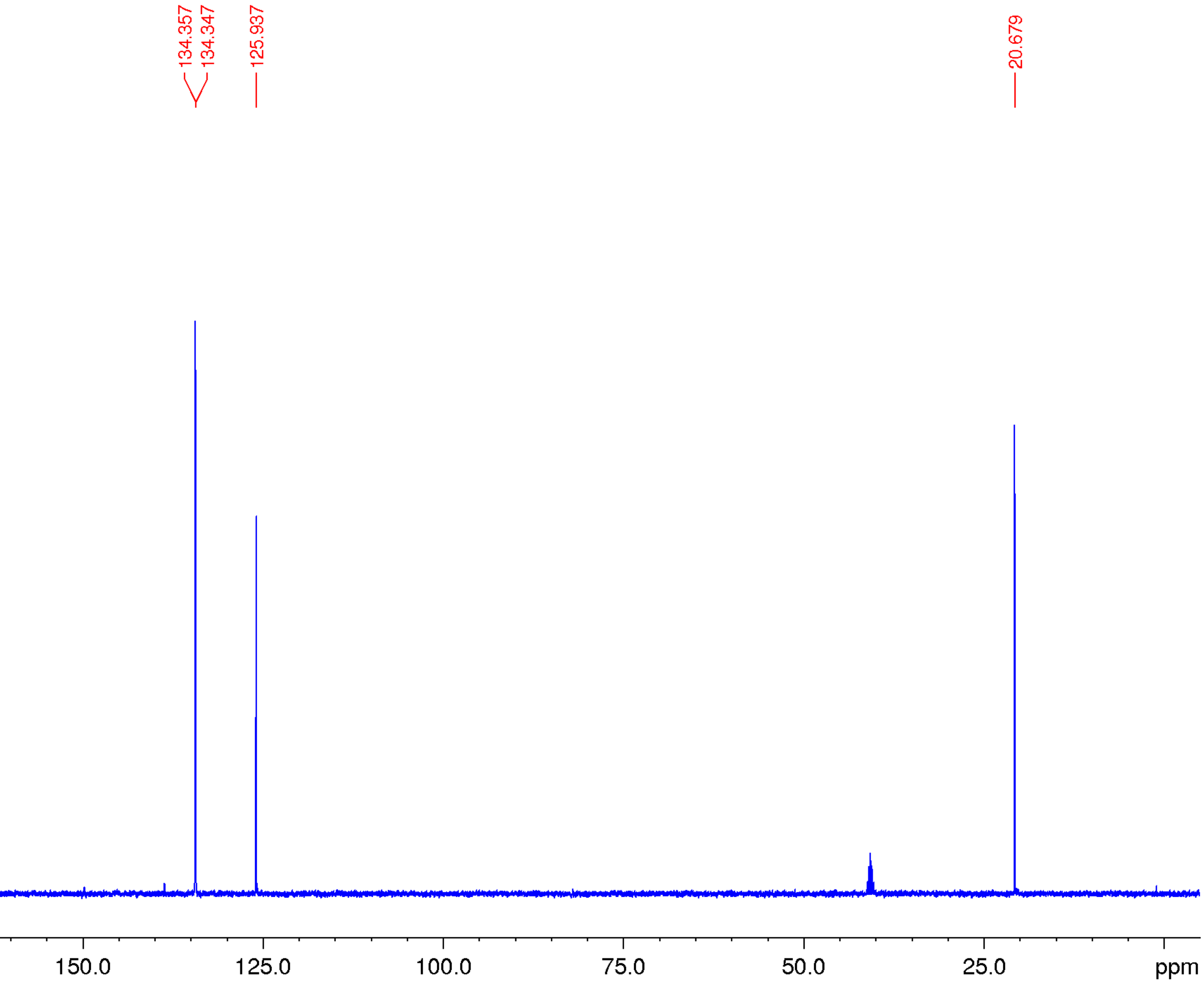

^{1}H NMR-spectrum (400 MHz, DMSO-d_6)

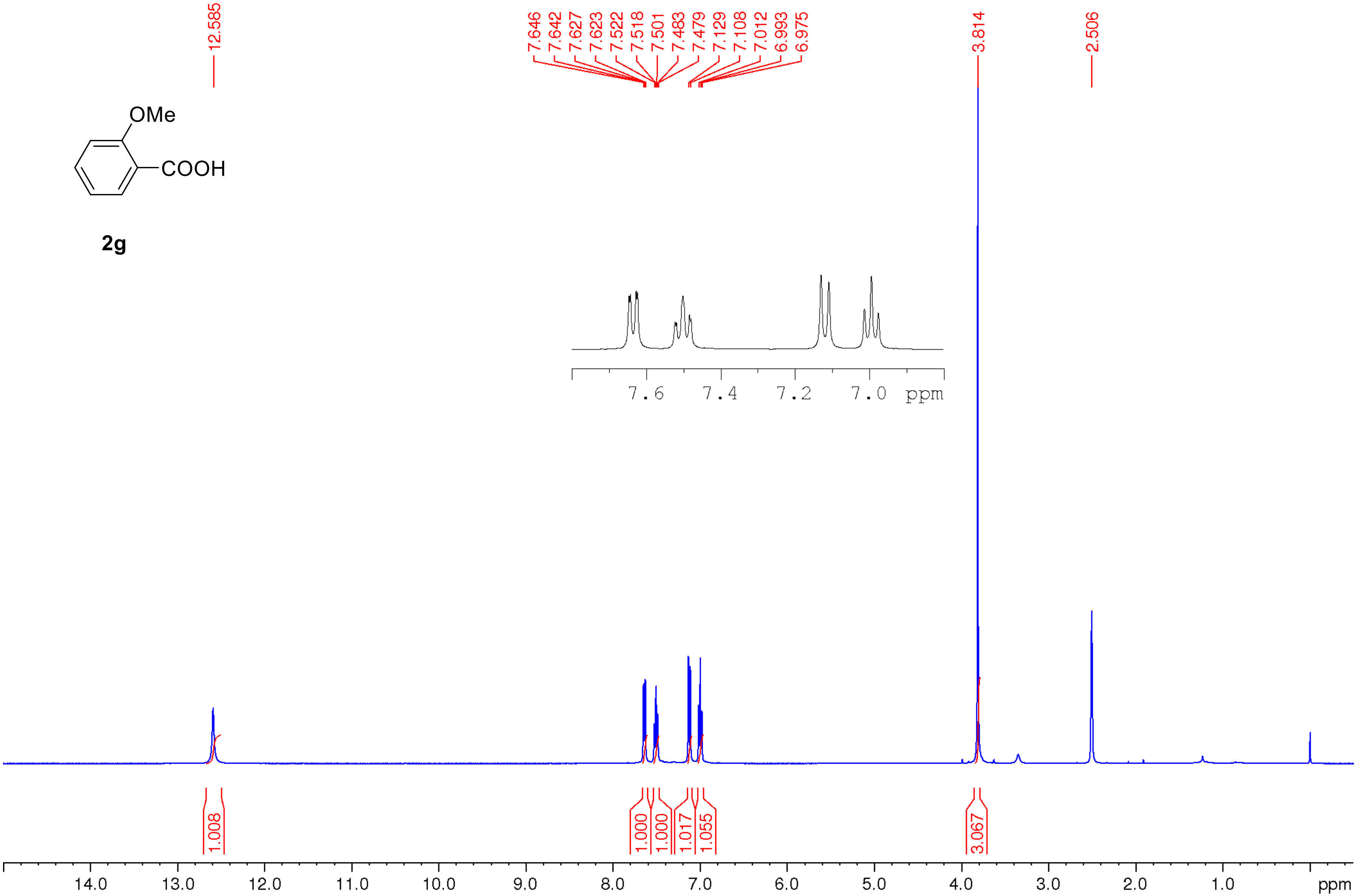

2g

^{13}C NMR-spectrum (100 MHz, DMSO-d$_6$)

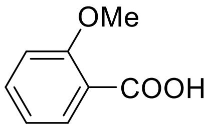

2g

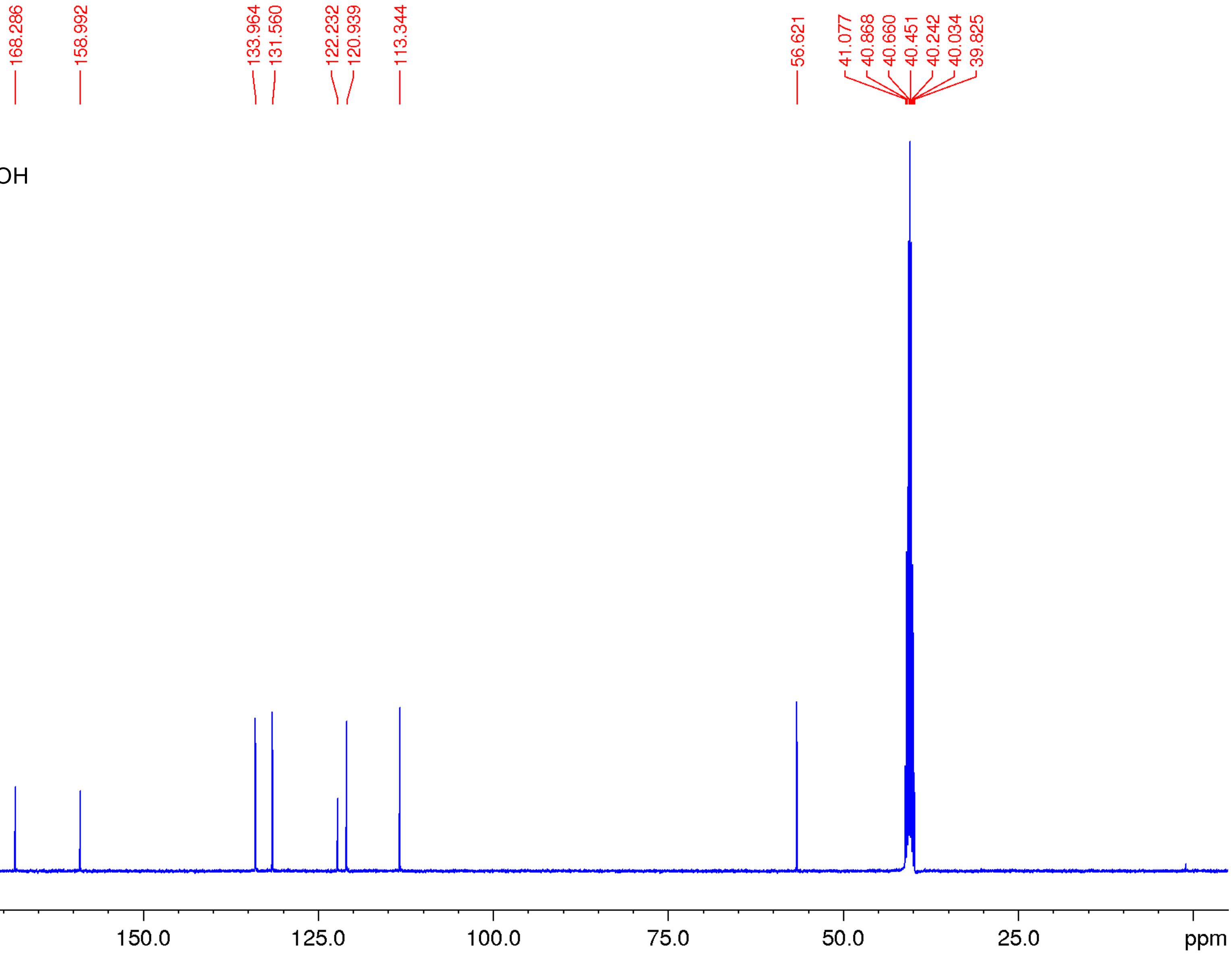

DEPT 135 NMR-spectrum (DMSO-d_6)

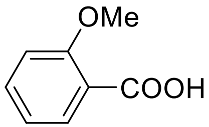

2g

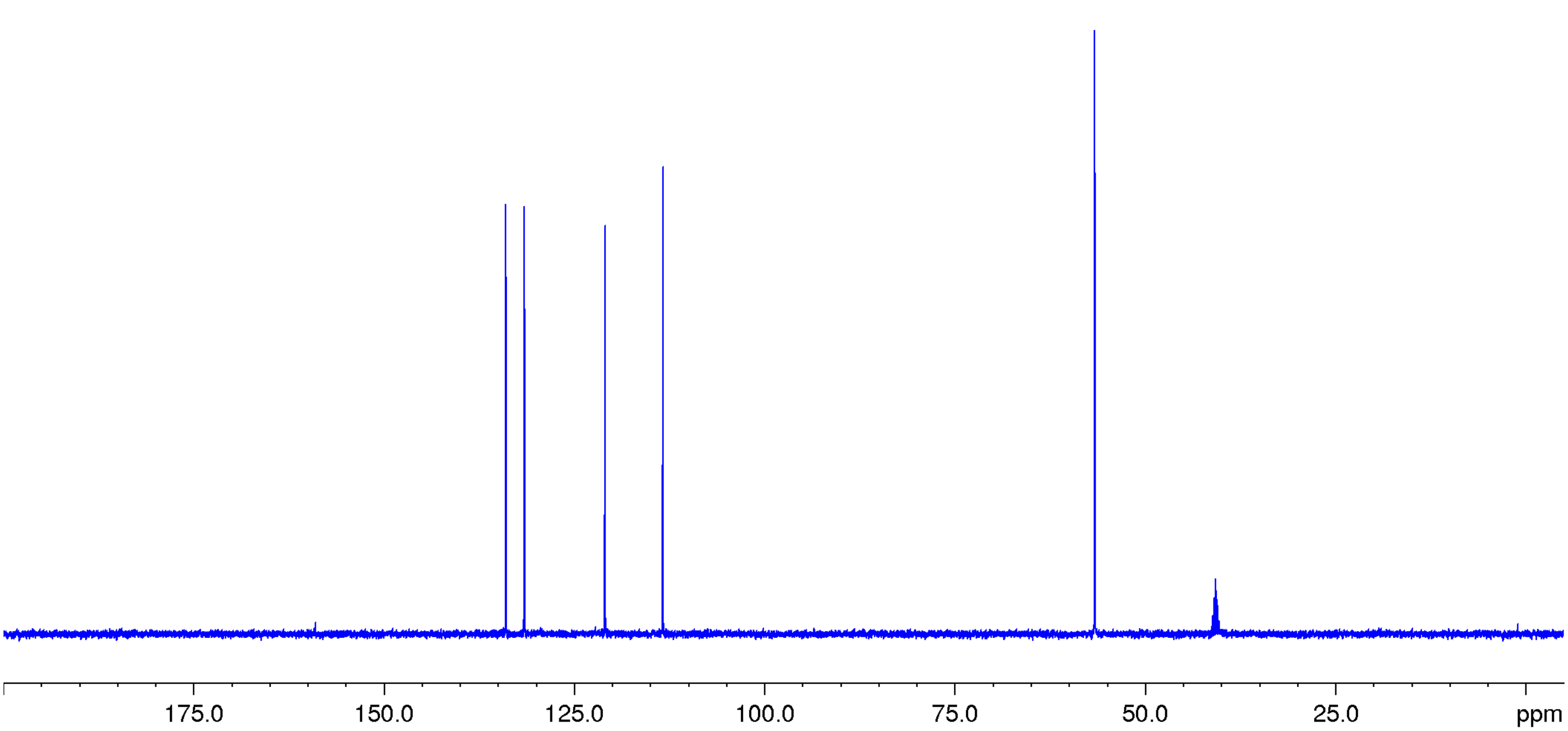

^{1}H NMR-spectrum (400 MHz, DMSO-d_6)

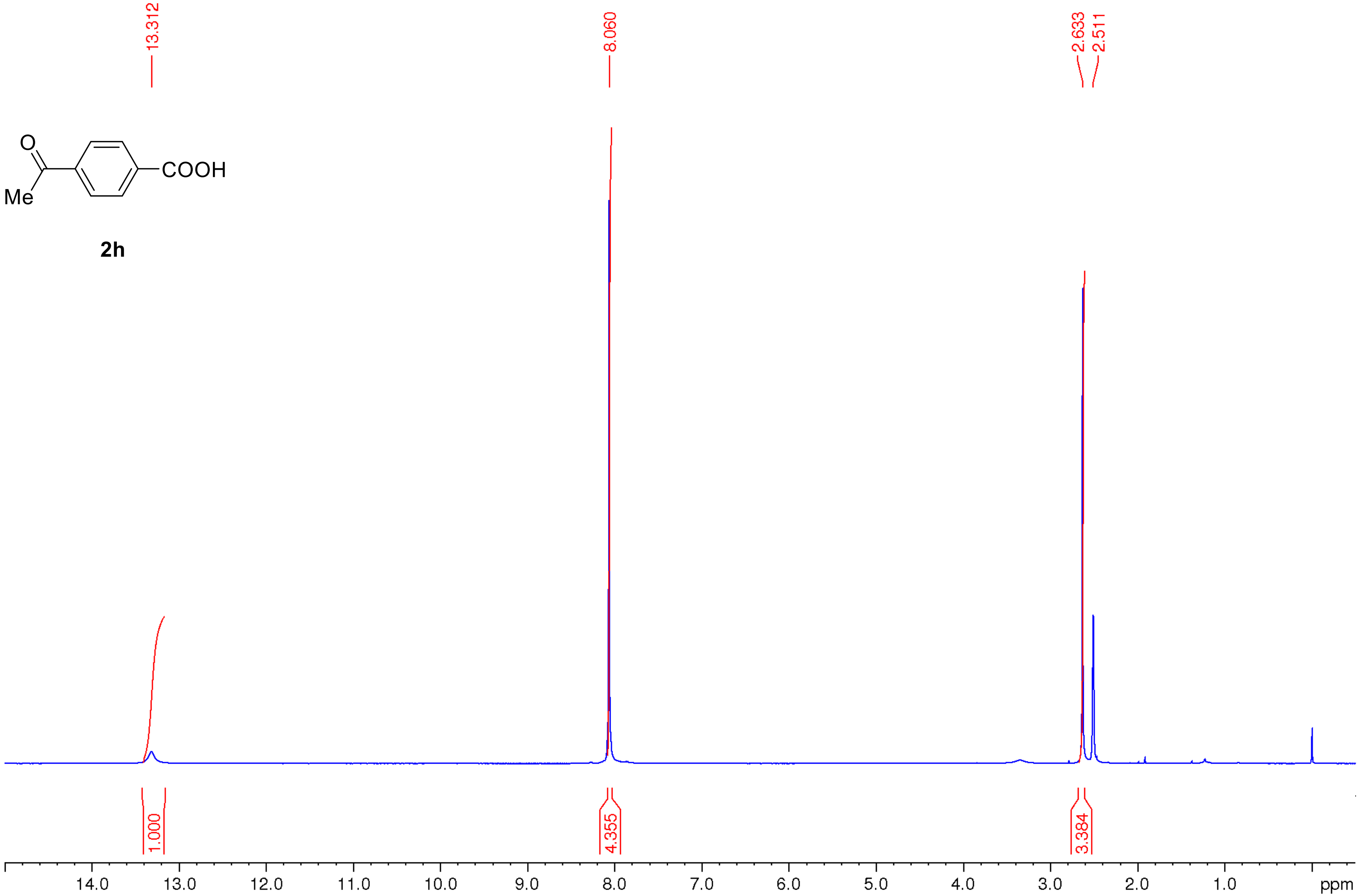

2h

^{13}C NMR-spectrum (100 MHz, DMSO-d_6)

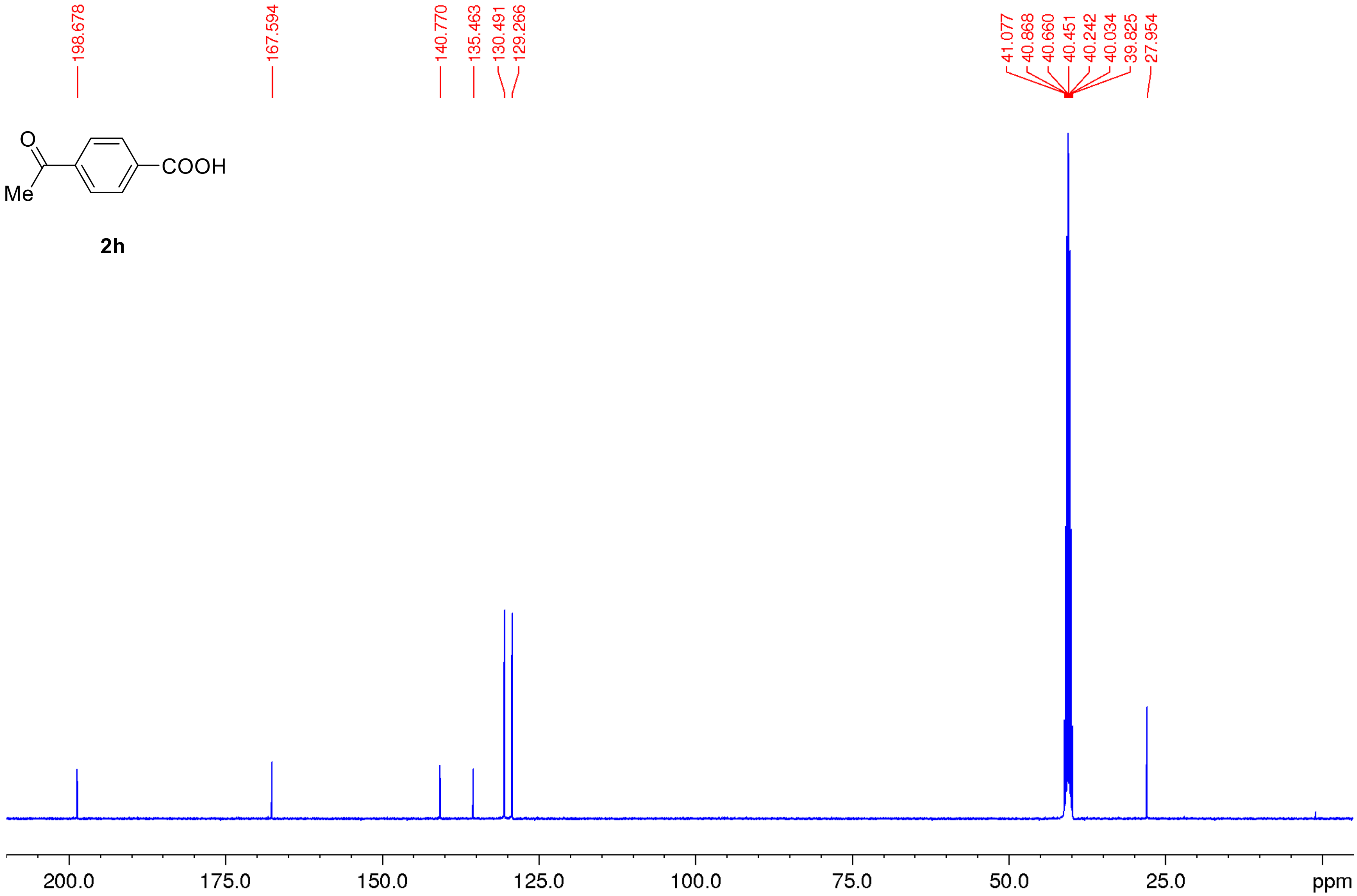

2h

DEPT 135 NMR-spectrum (DMSO-d_6)

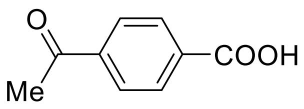

2h

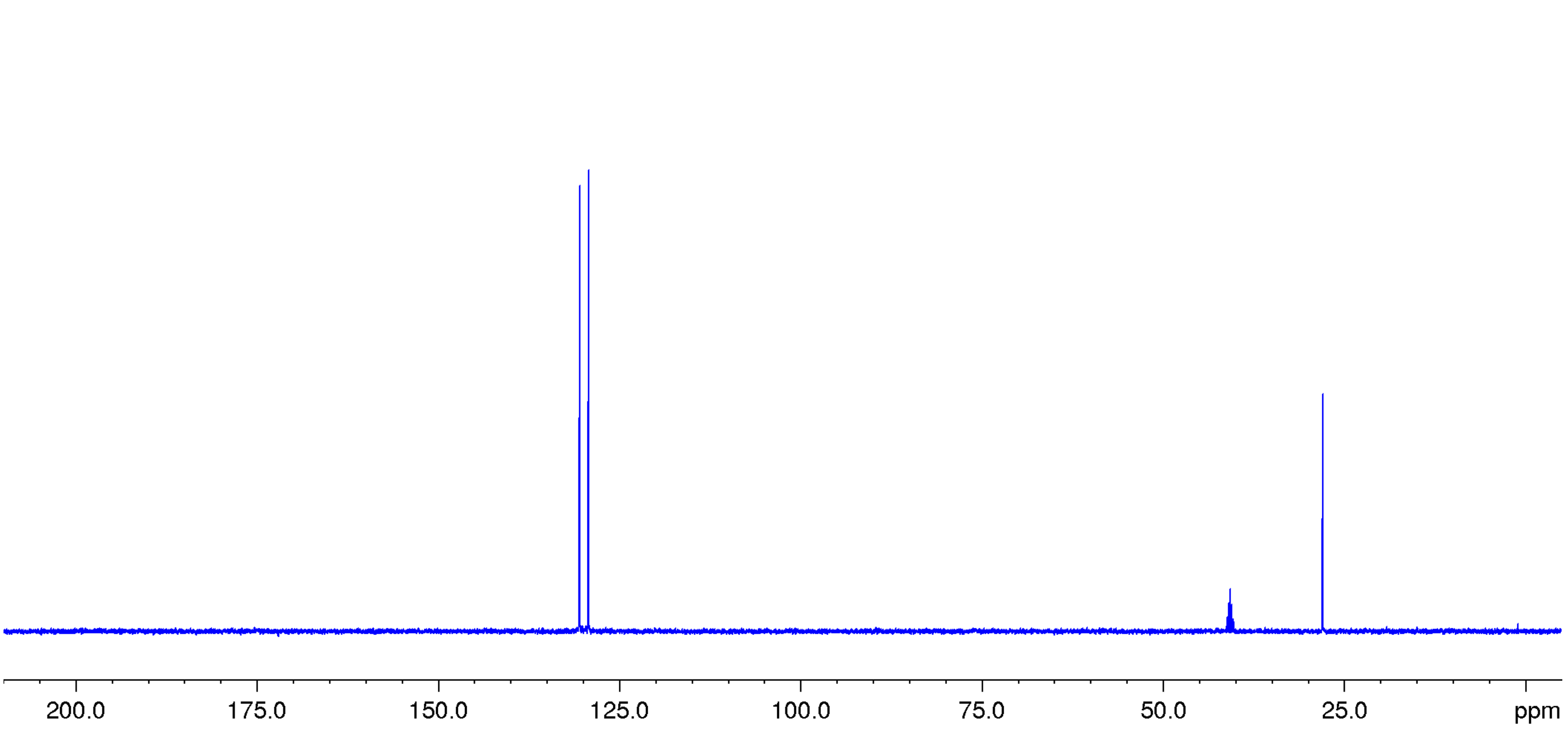

^{1}H NMR-spectrum (400 MHz, DMSO-d_6)

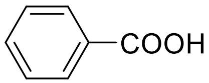

2i

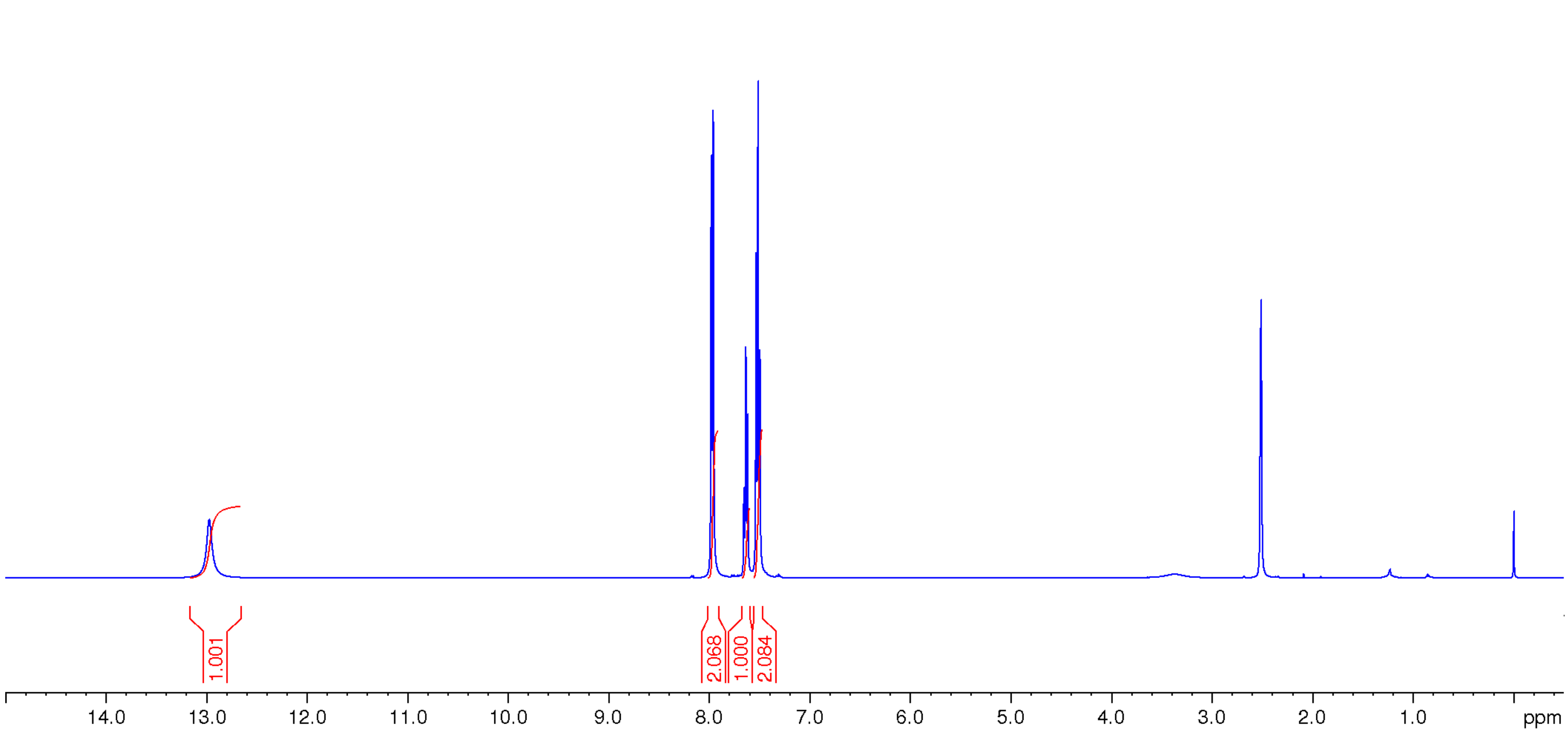

^{13}C NMR-spectrum (100 MHz, DMSO-d_6)

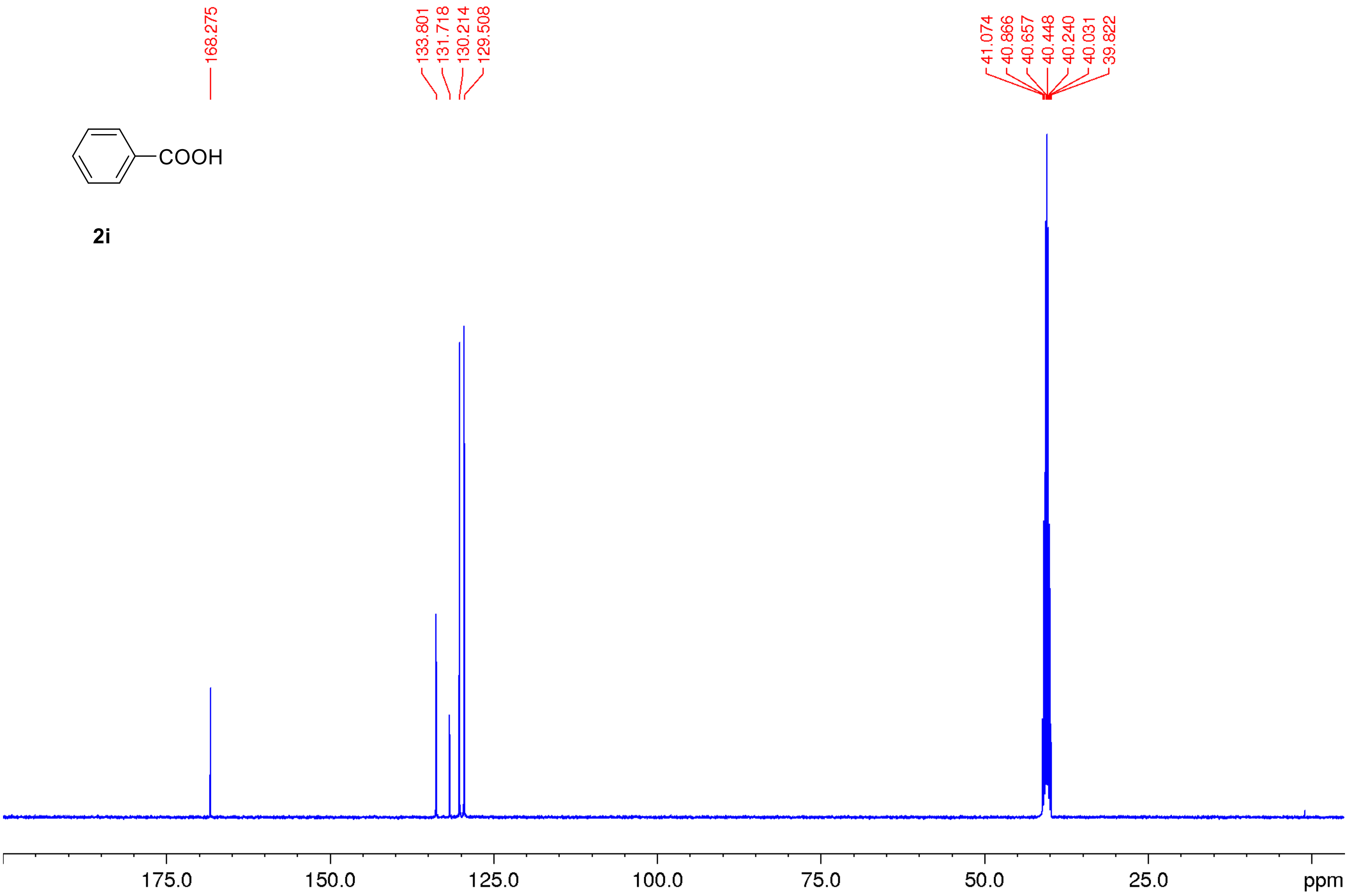

DEPT 135 NMR-spectrum (DMSO-d_6)

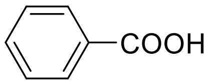

2i

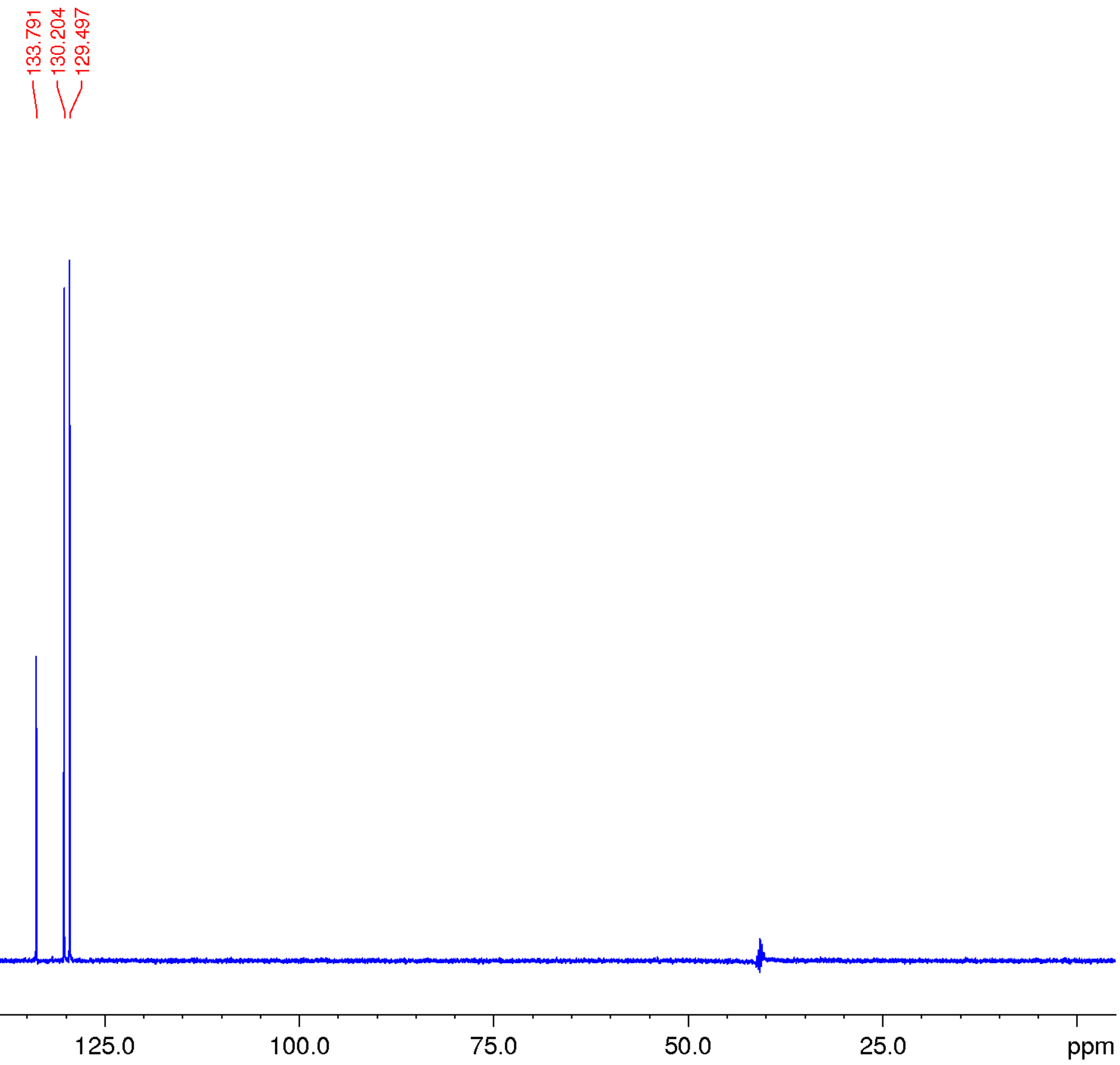

^{1}H NMR-spectrum (400 MHz, DMSO-d_6)

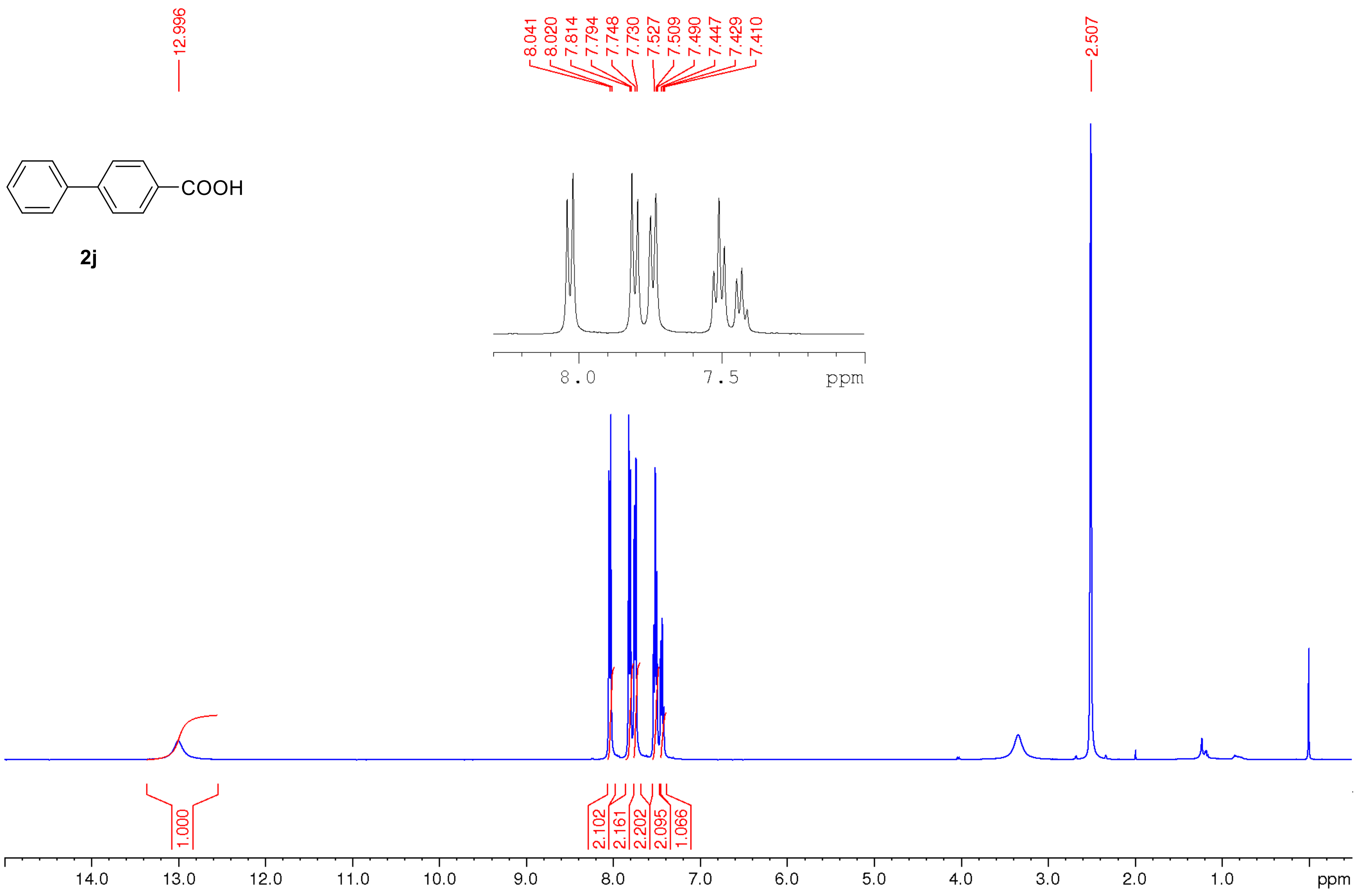

^{13}C NMR-spectrum (100 MHz, DMSO-d_6)

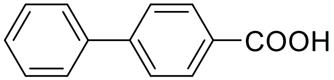

2j

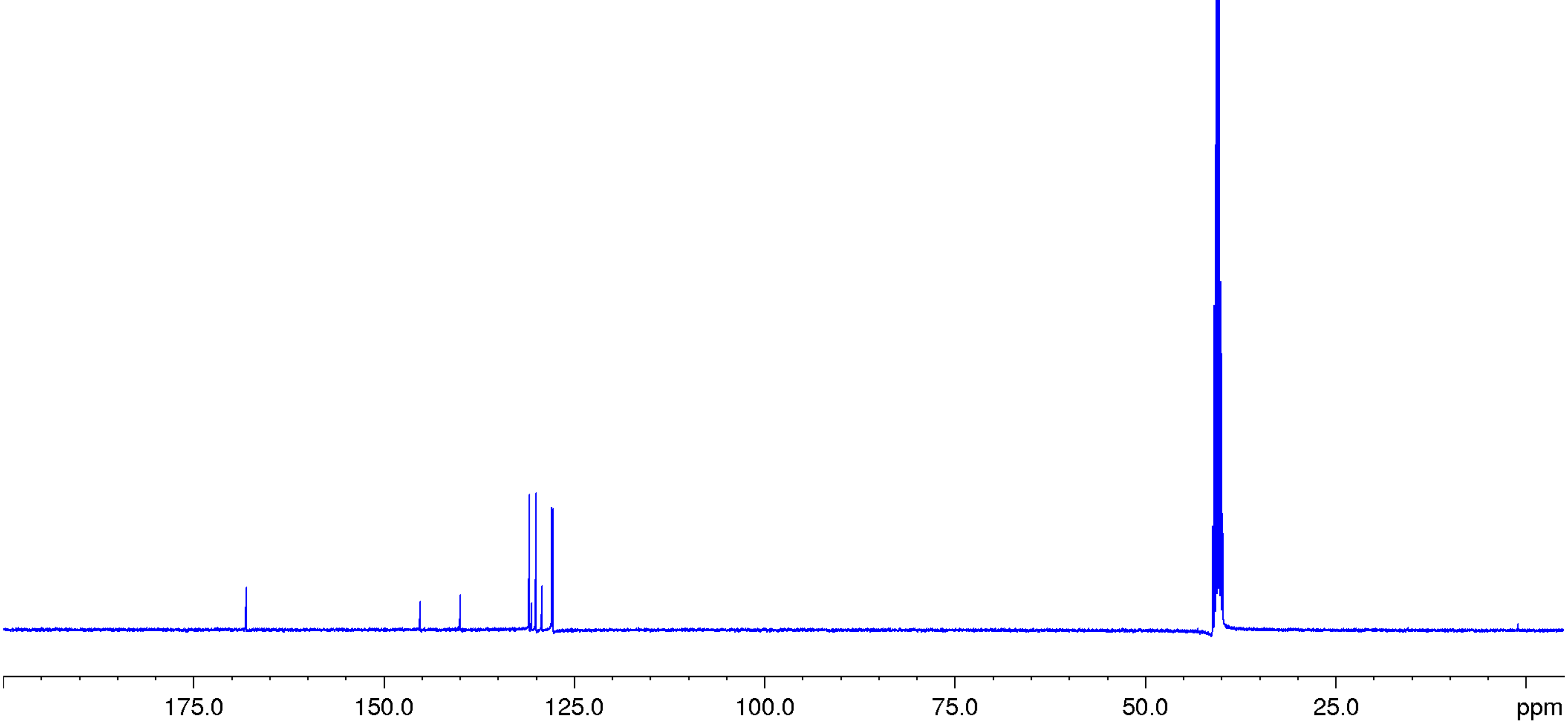

DEPT 135 NMR-spectrum (DMSO-d_6)

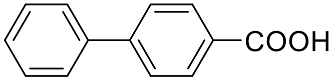

2j

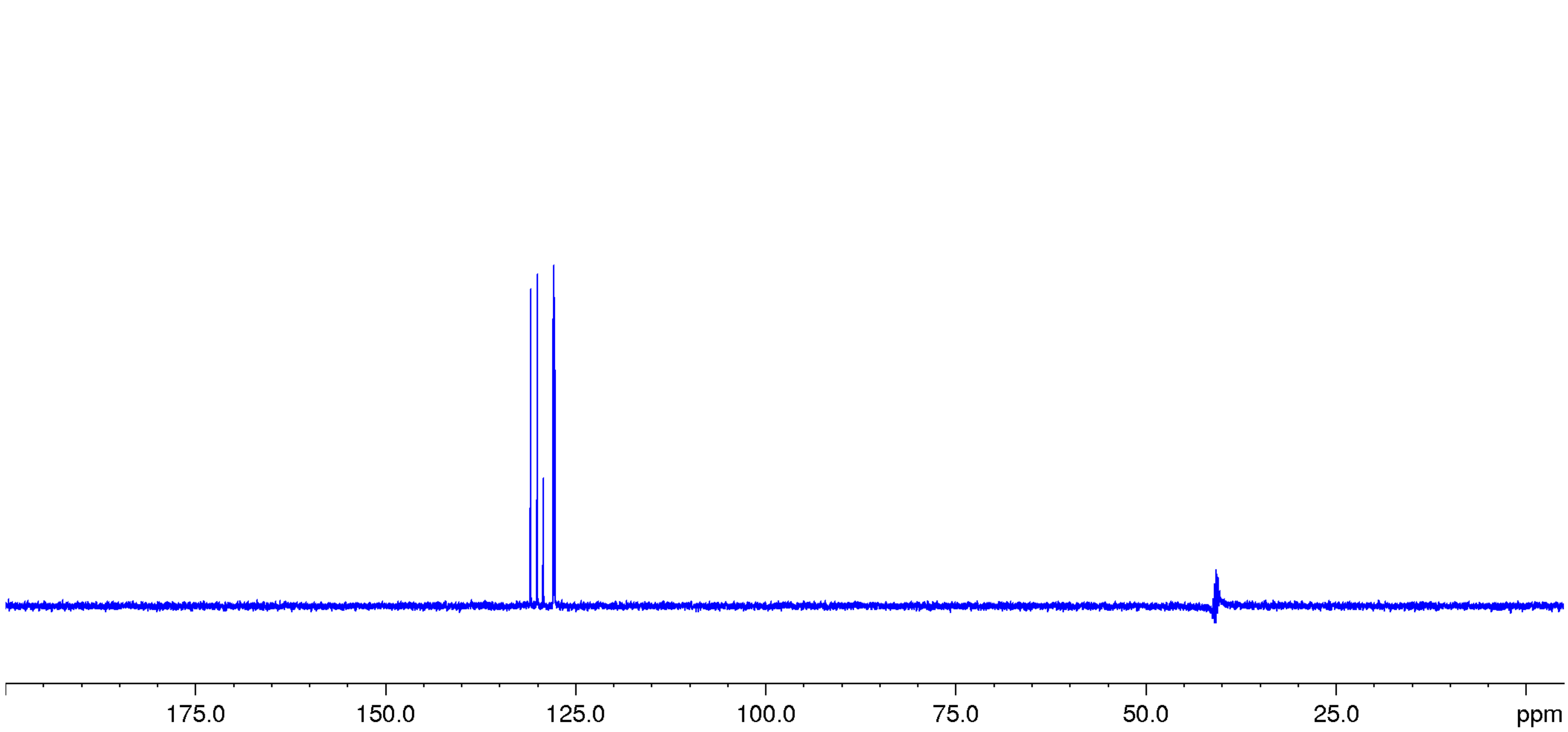

^{1}H NMR-spectrum (400 MHz, DMSO-d_6)

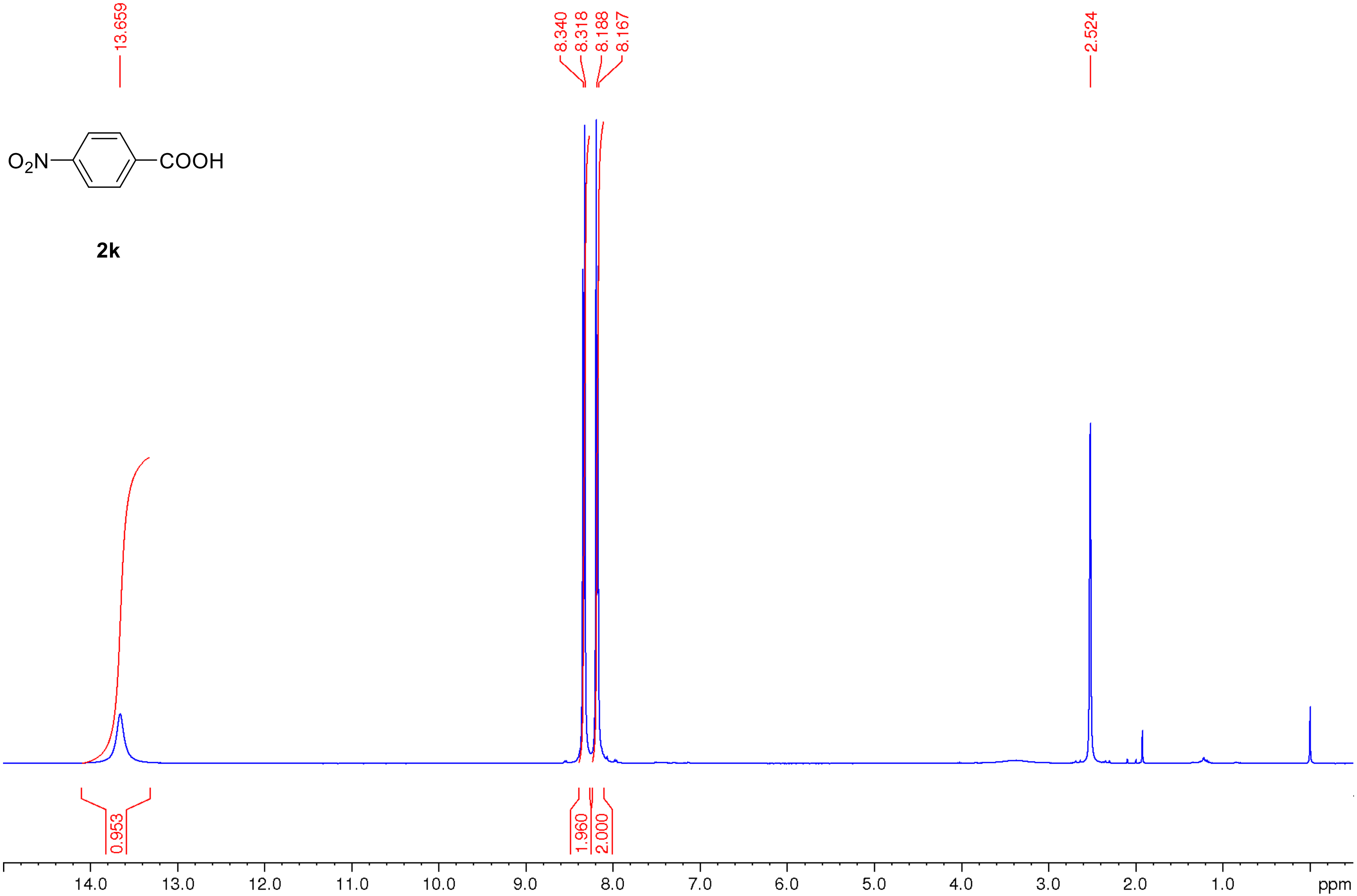

2k

^{13}C NMR-spectrum (100 MHz, DMSO-d_6)

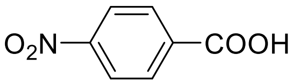

2k

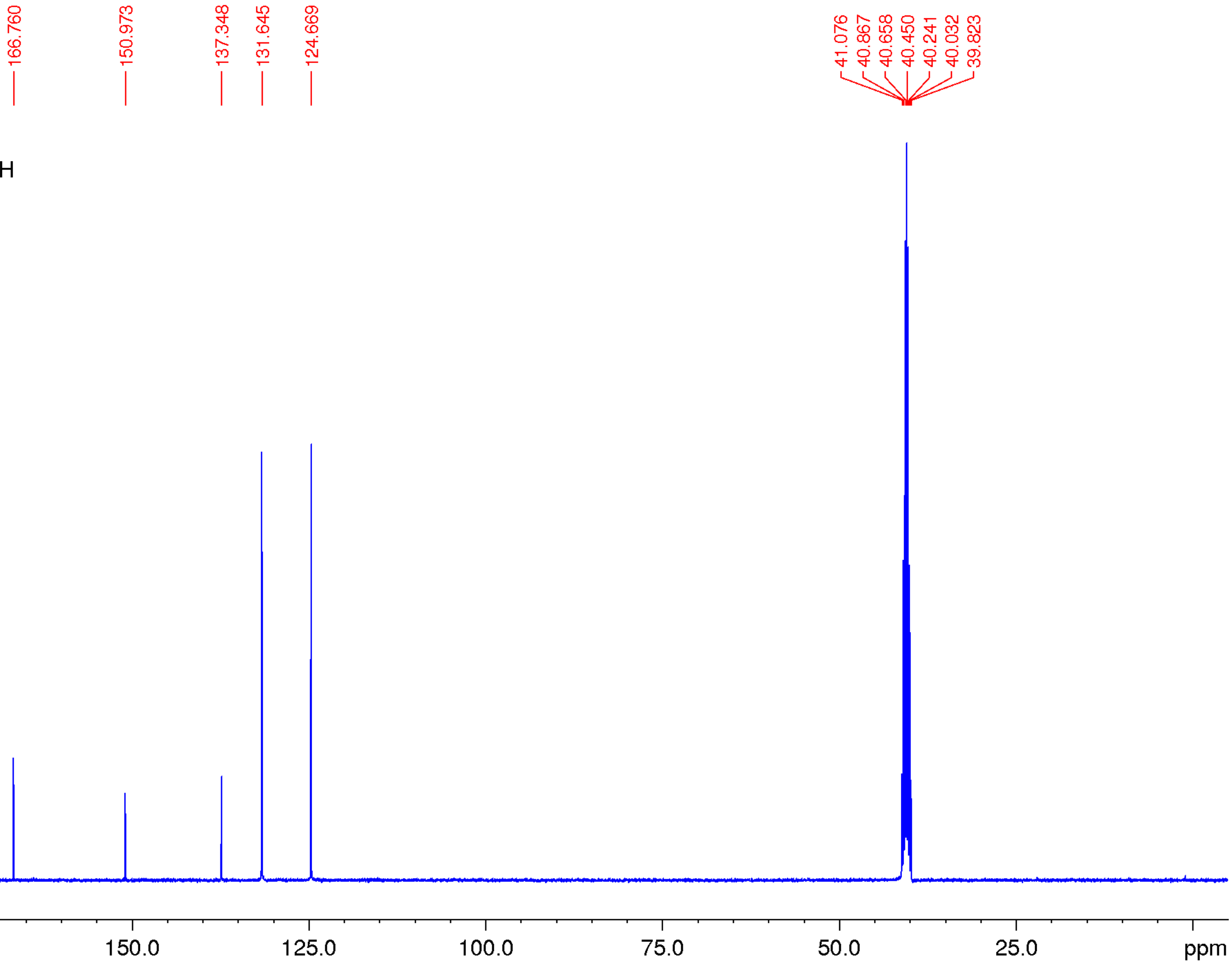

DEPT 135 NMR-spectrum (DMSO-d_6)

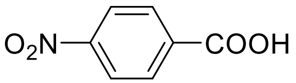

2k

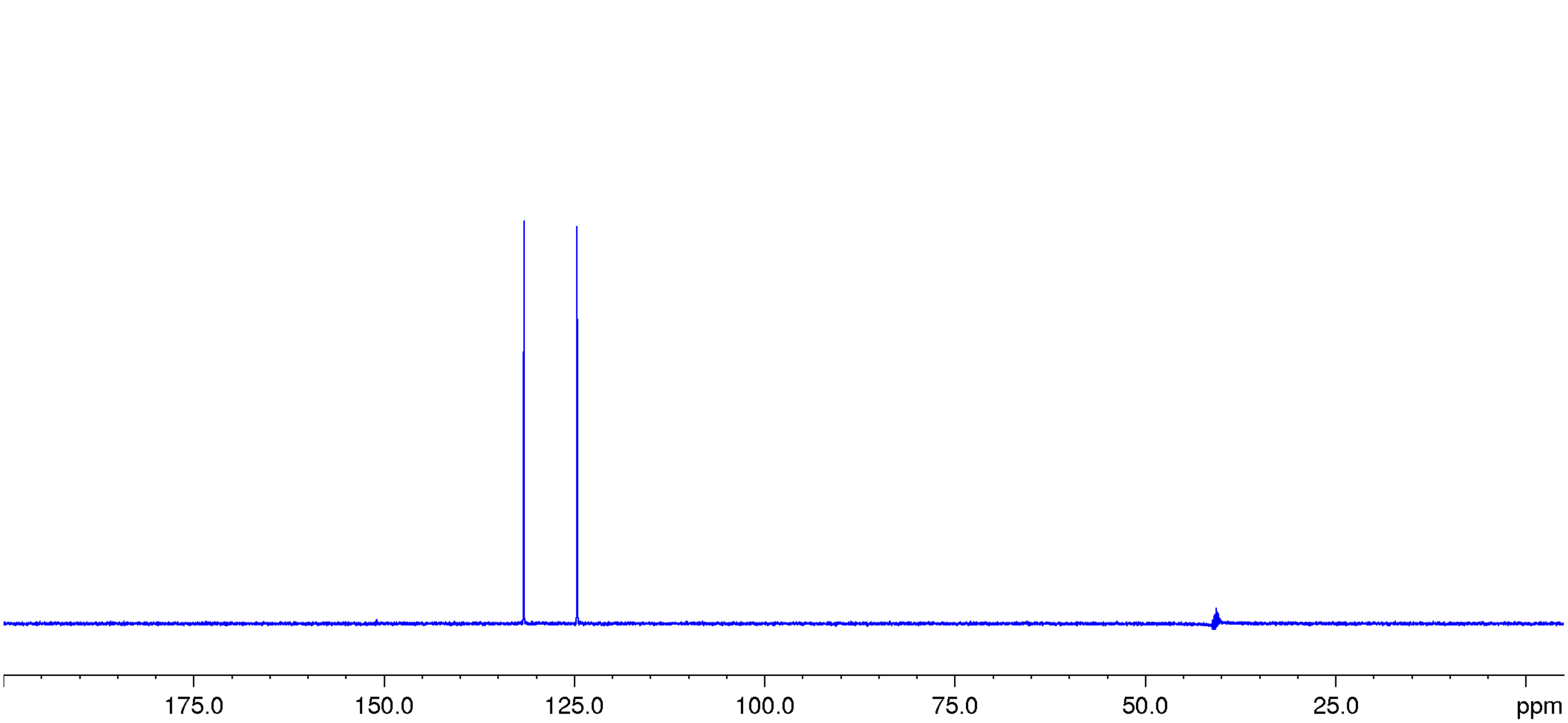

^{1}H NMR-spectrum (400 MHz, $CDCl_3$)

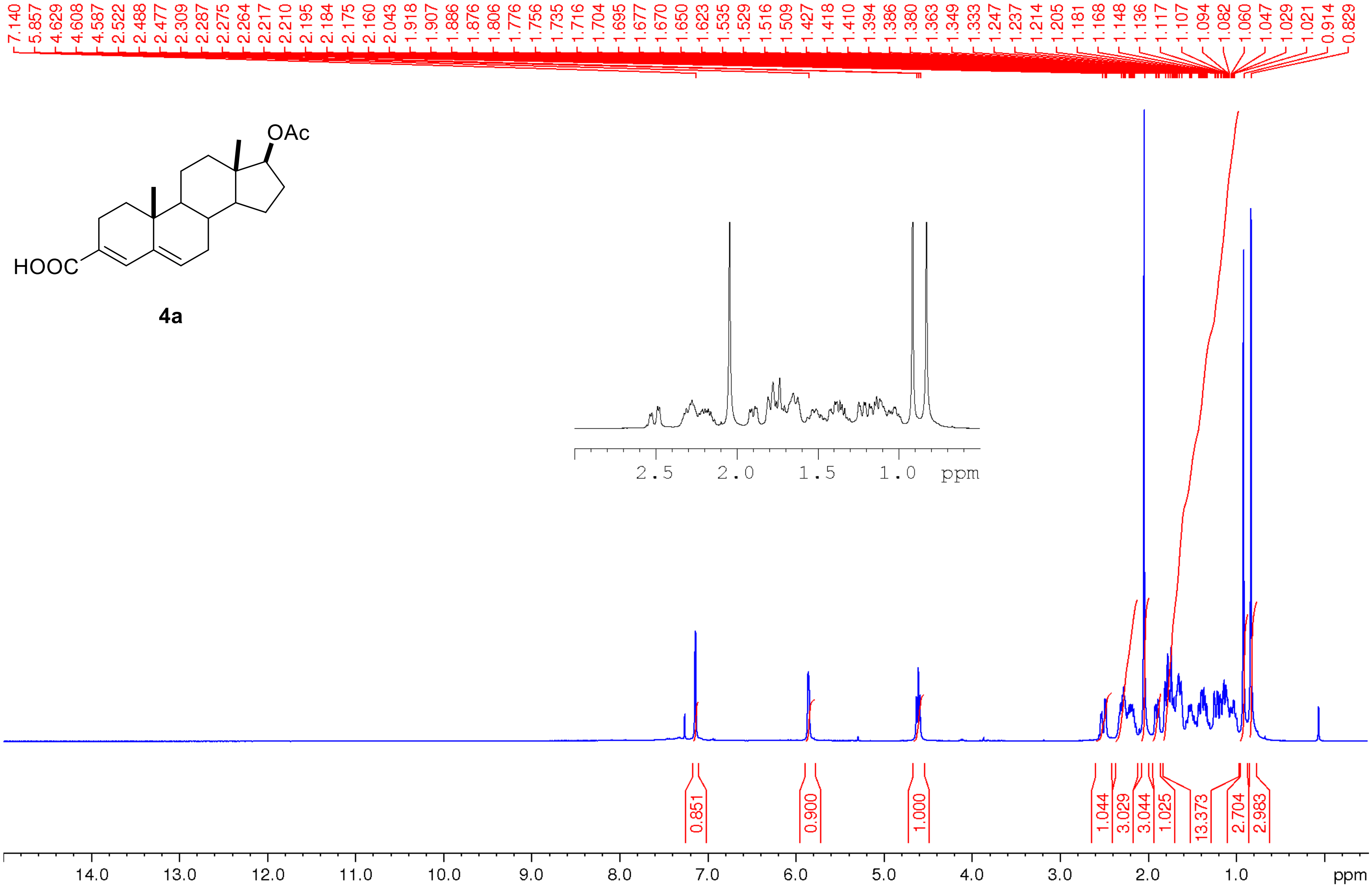

^{13}C NMR-spectrum (100 MHz, $CDCl_3$)

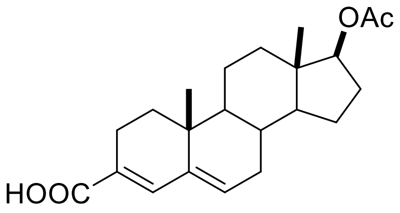

4a

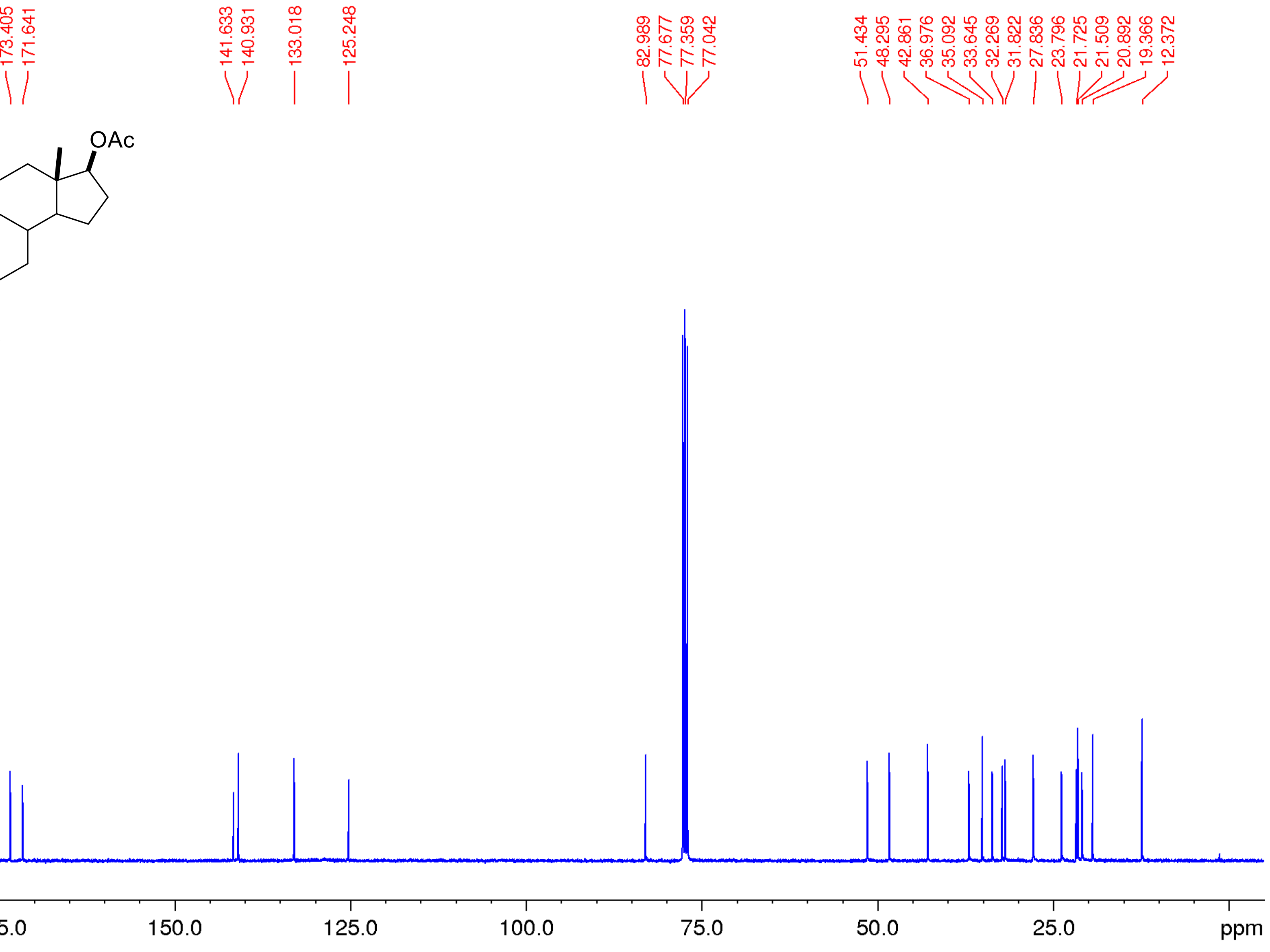

DEPT 135 NMR-spectrum ($CDCl_3$)

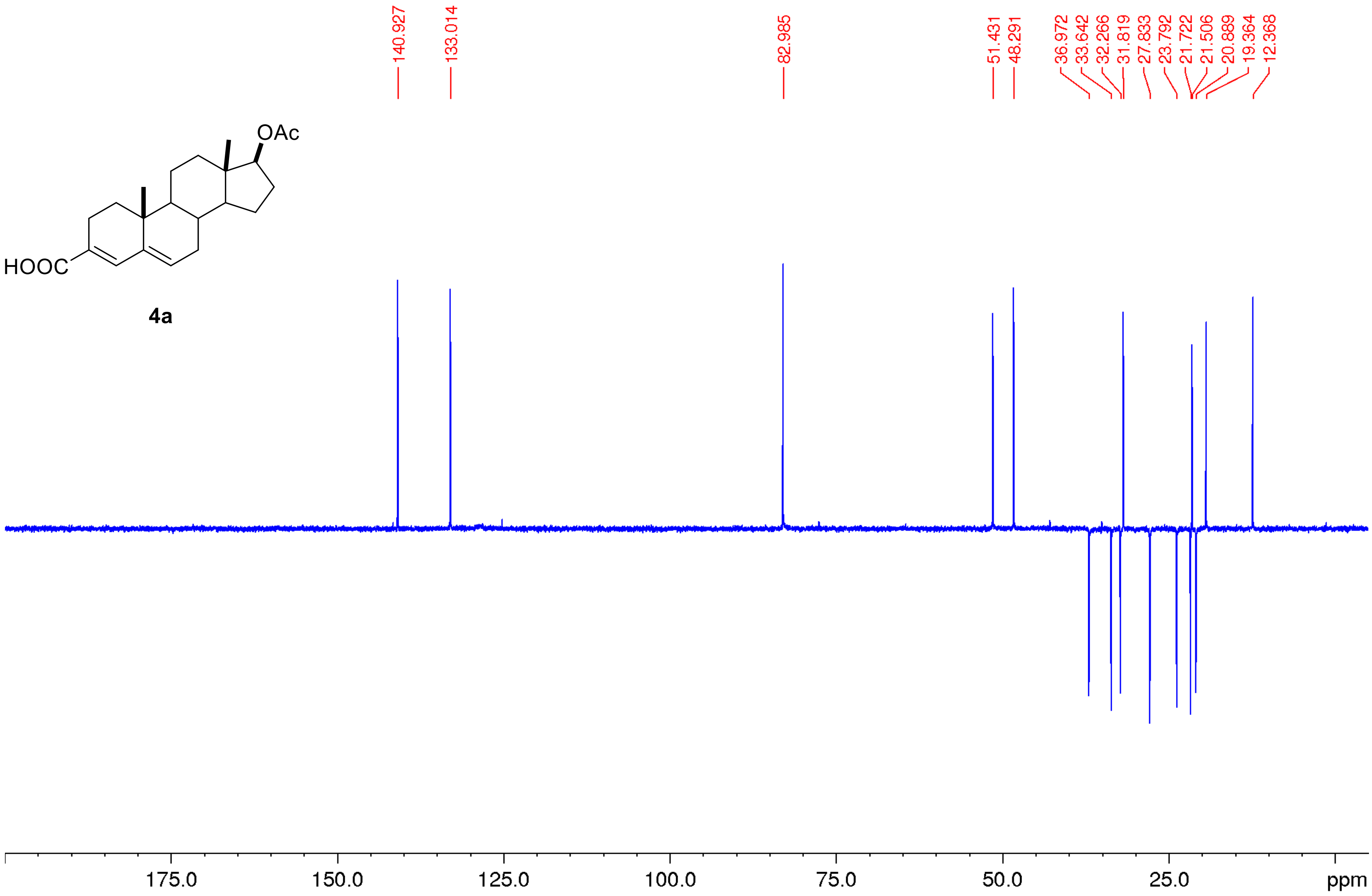

^{1}H NMR-spectrum (400 MHz, $CDCl_3$)

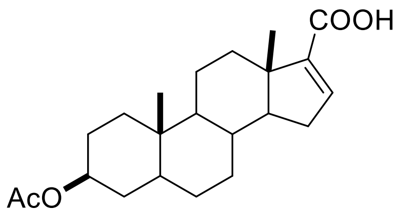

4b

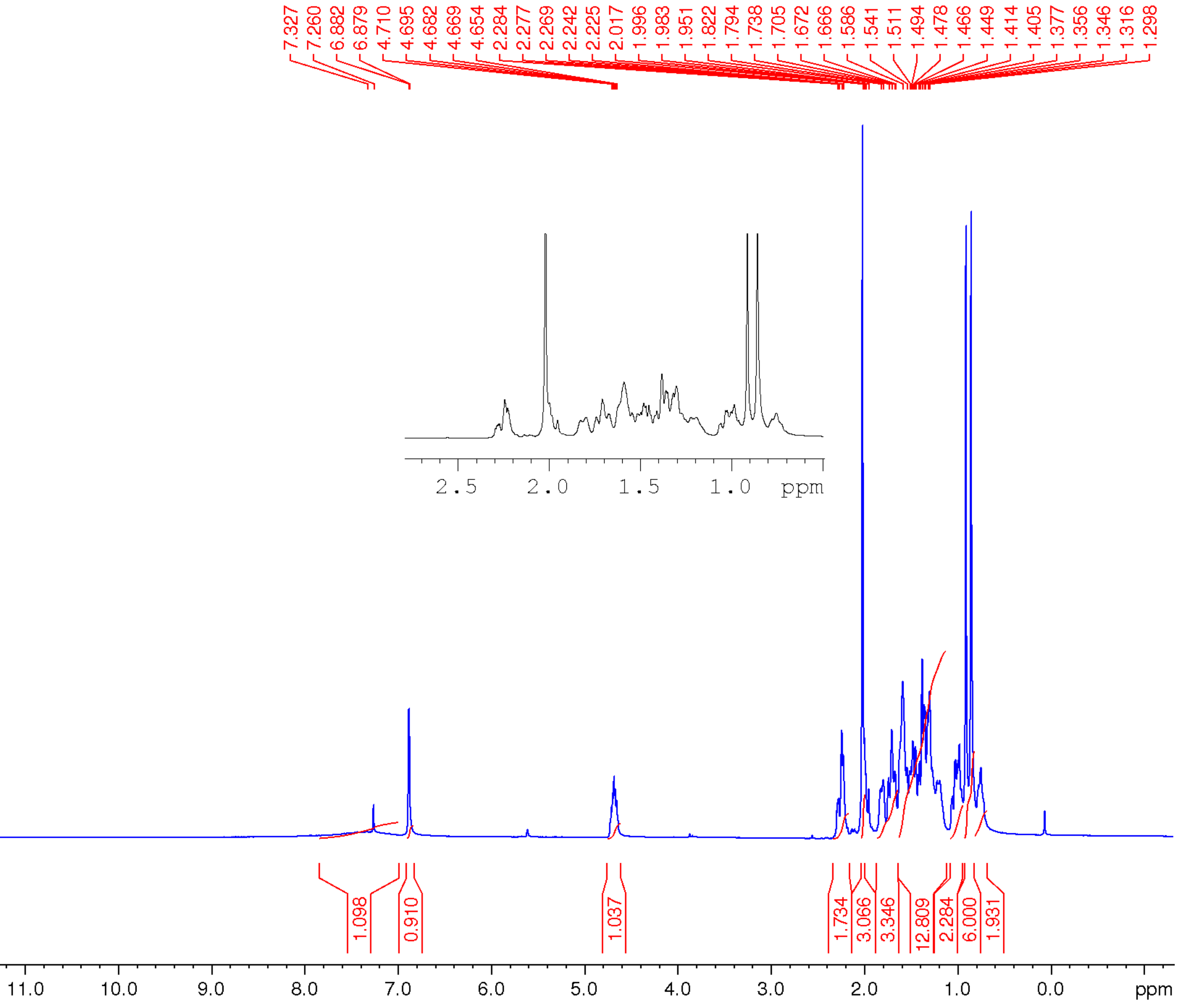

^{13}C NMR-spectrum (100 MHz, $CDCl_3$)

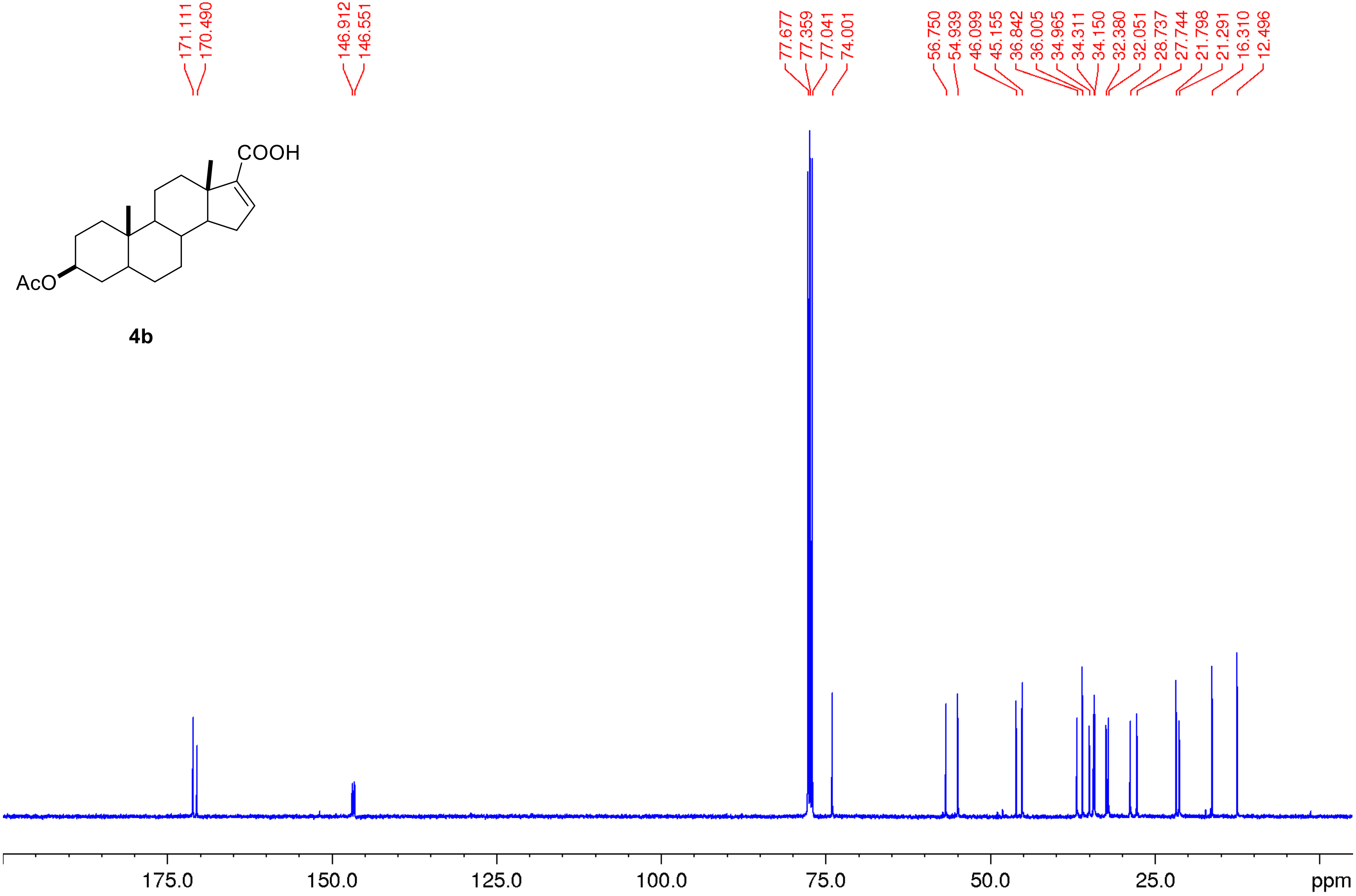

DEPT 135 NMR-spectrum ($CDCl_3$)

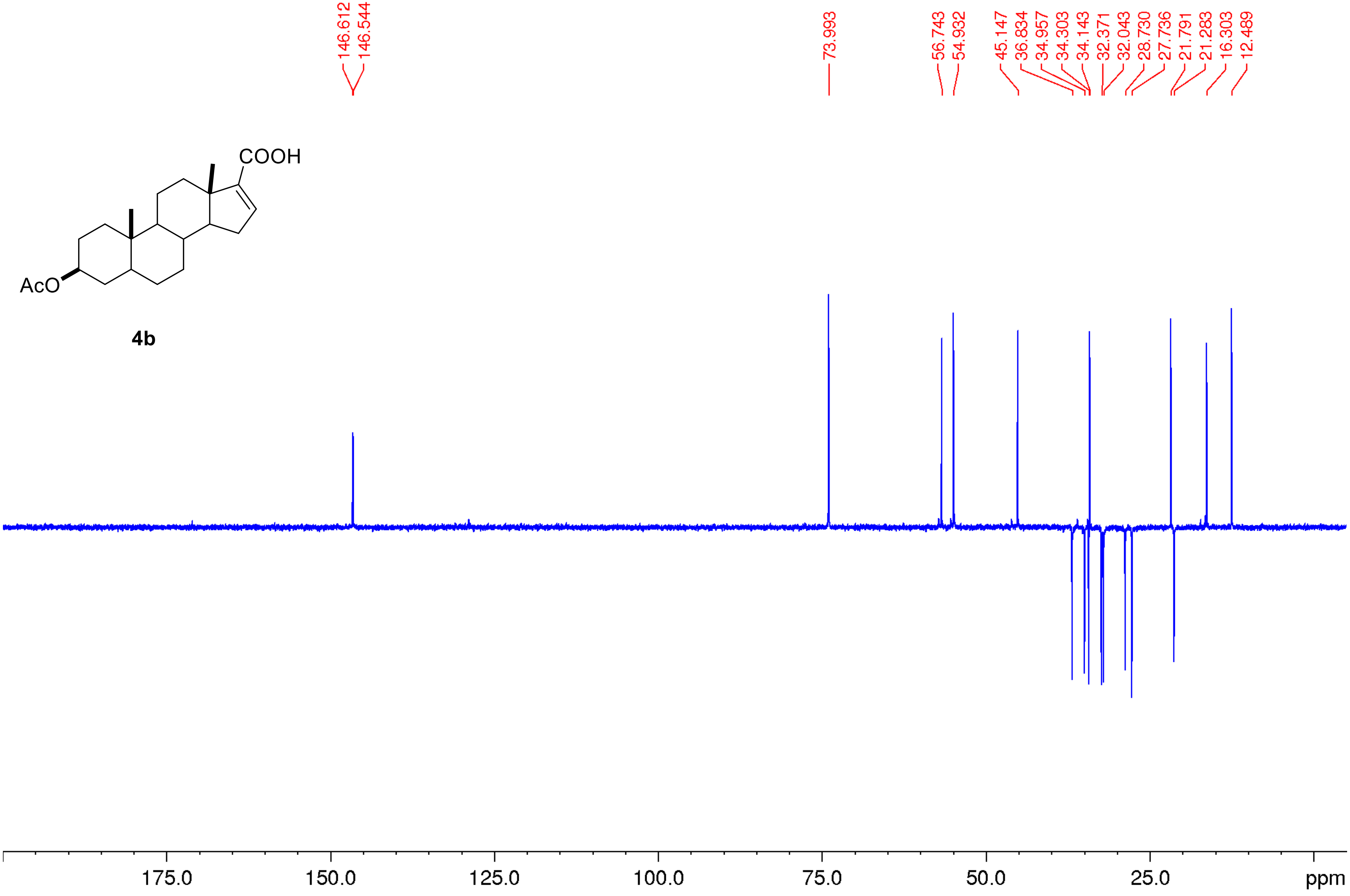

^{1}H NMR-spectrum (400 MHz, $CDCl_3$)

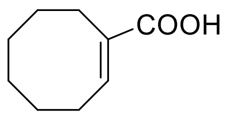

4c

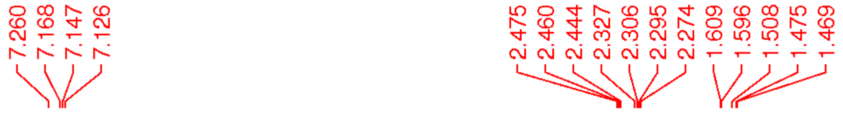

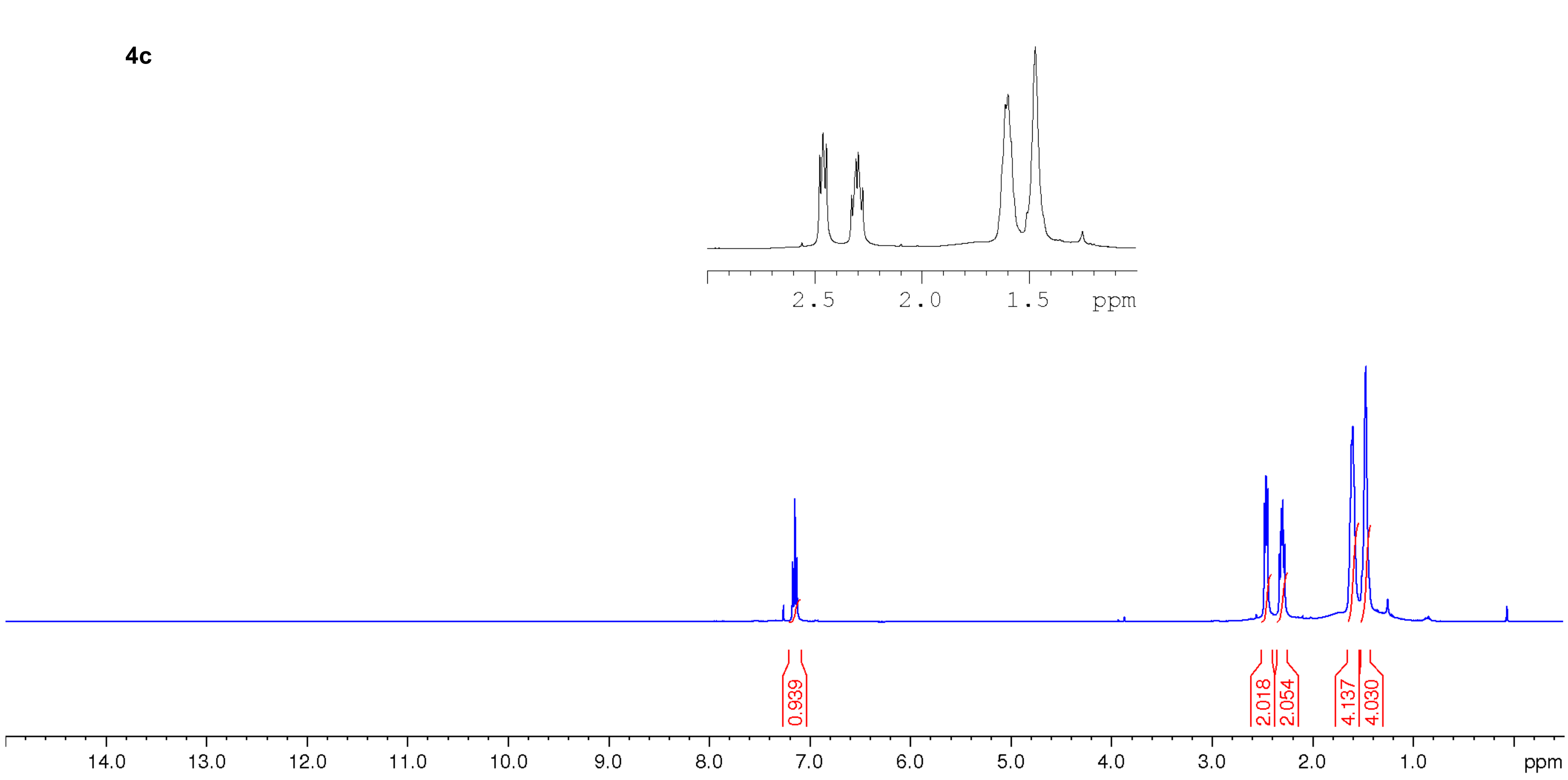

^{13}C NMR-spectrum (100 MHz, $CDCl_3$)

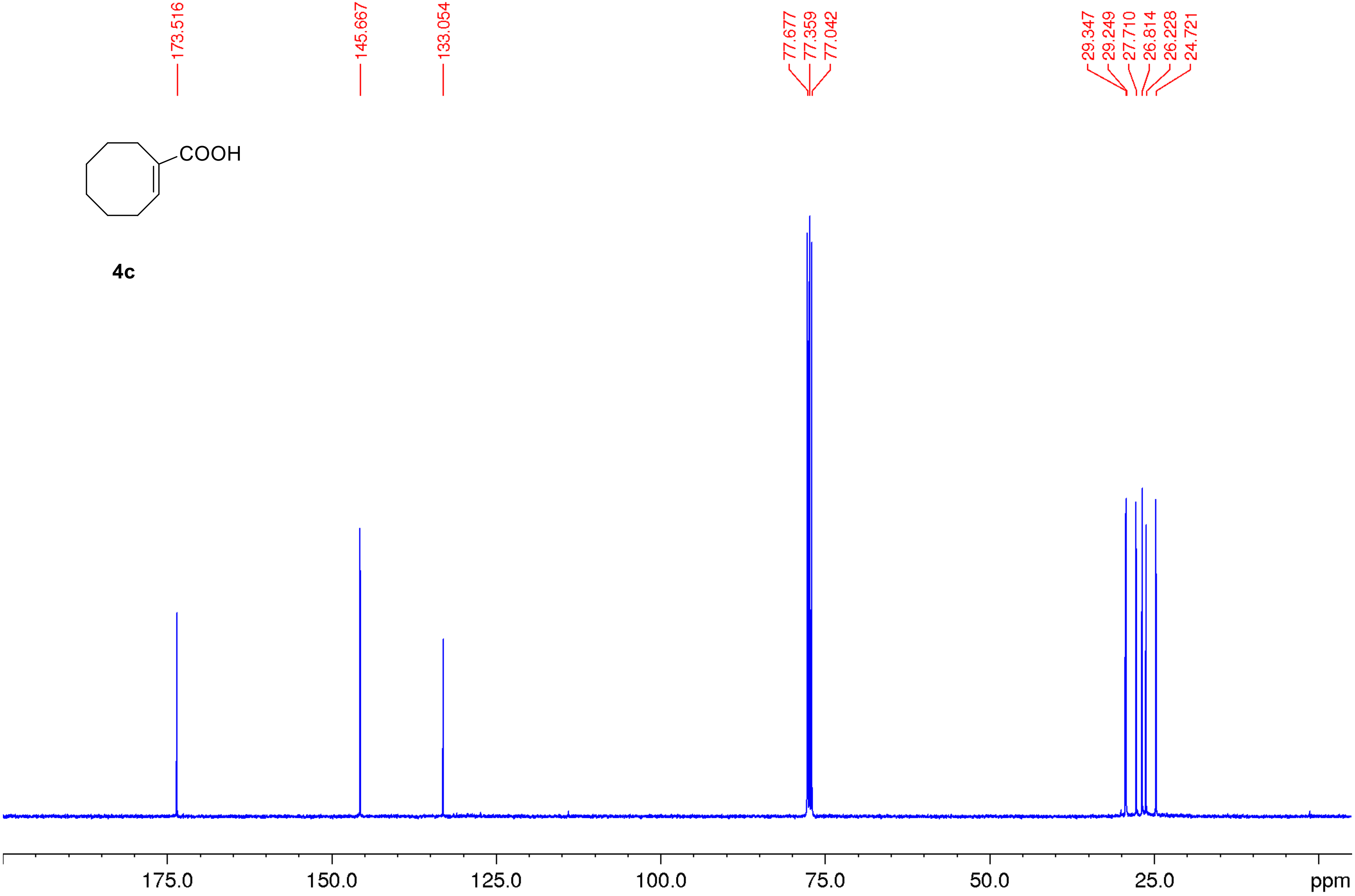

DEPT 135 NMR-spectrum ($CDCl_3$)

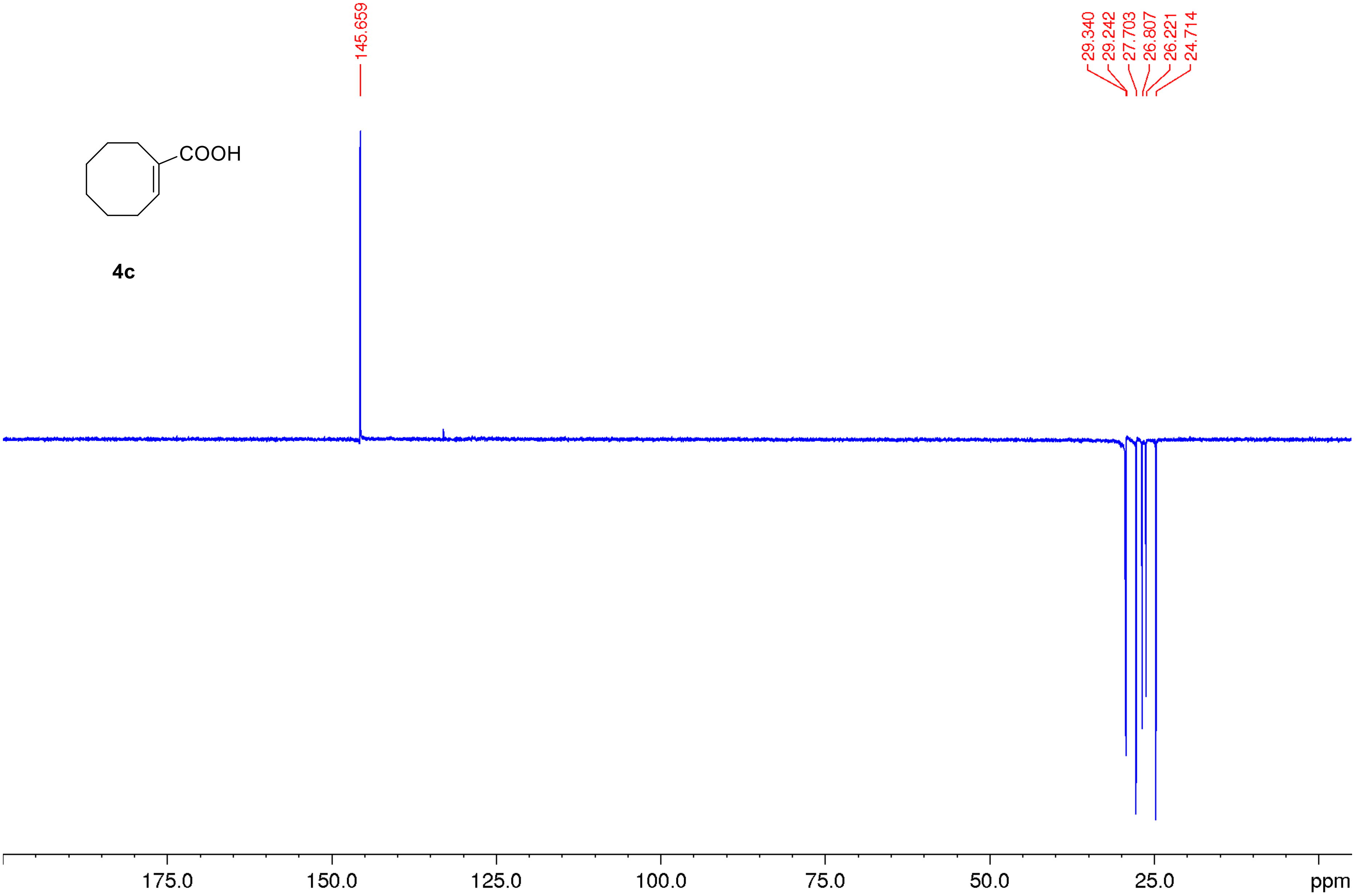

4c

^1H NMR-spectrum (400 MHz, $CDCl_3$)

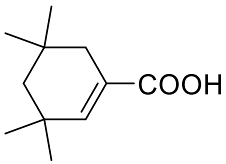

4d

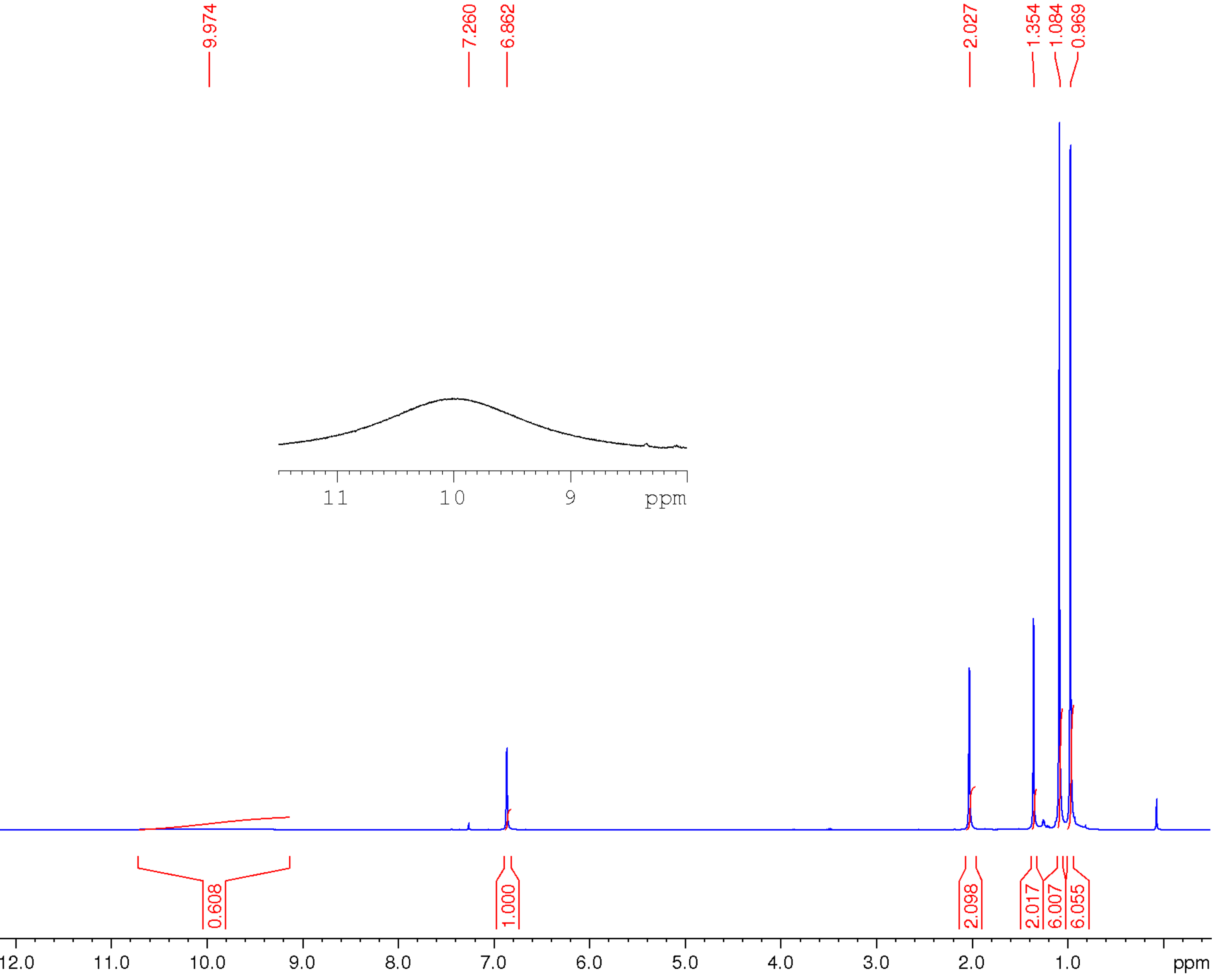

^{13}C NMR-spectrum (100 MHz, $CDCl_3$)

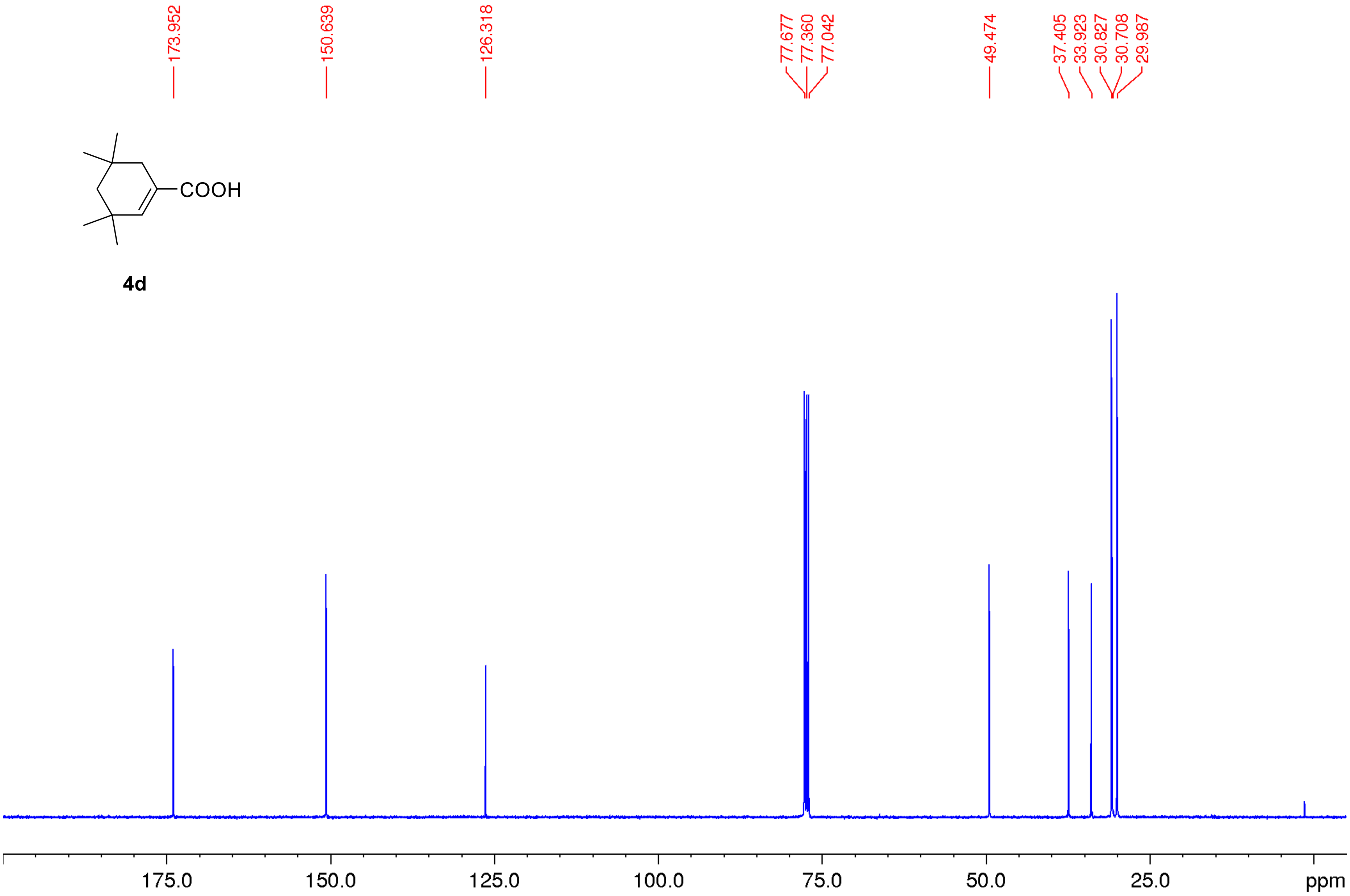

4d

DEPT 135 NMR-spectrum ($CDCl_3$)

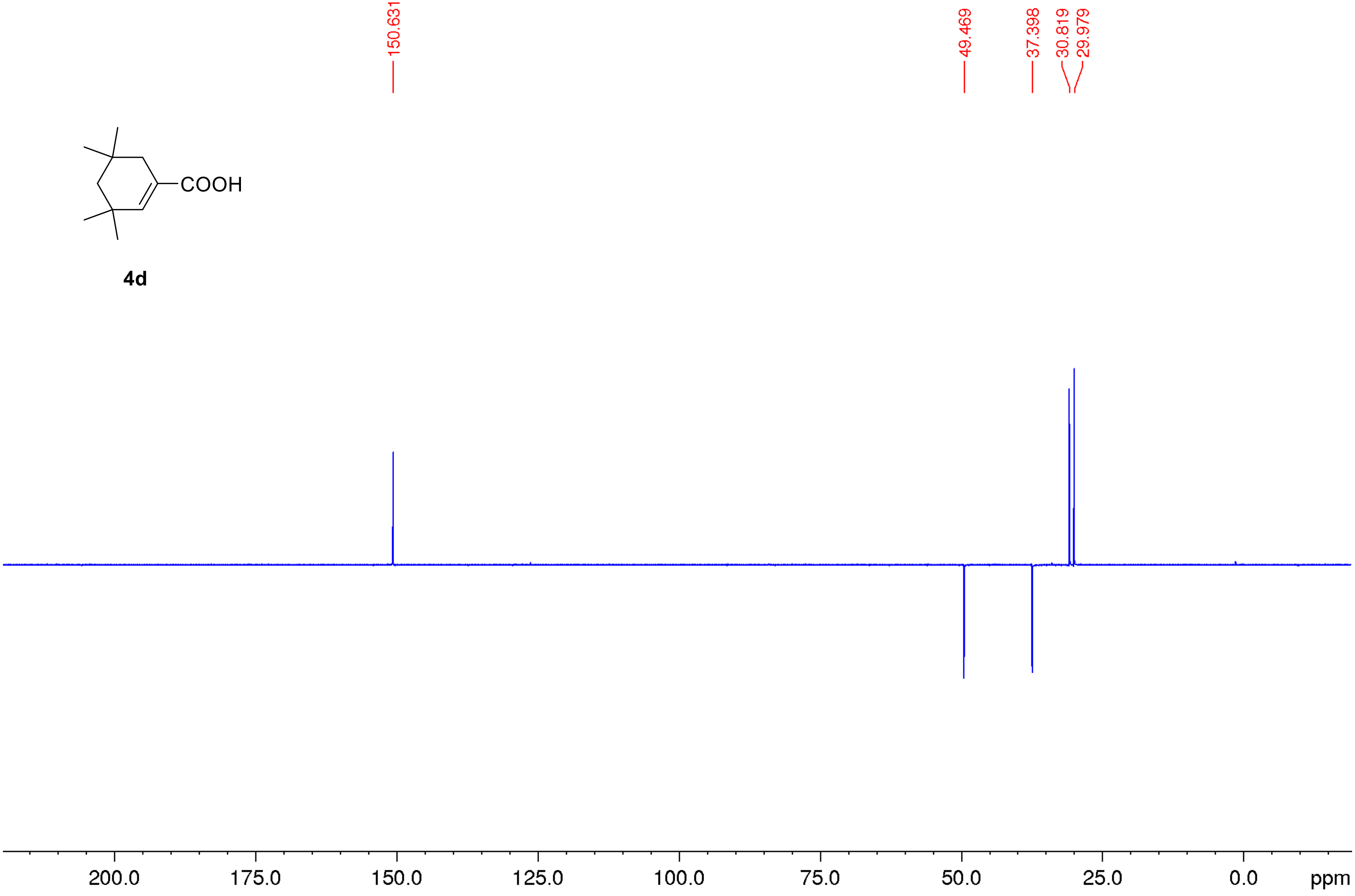

^{1}H NMR-spectrum (400 MHz, $CDCl_3$)

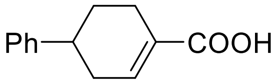

4e

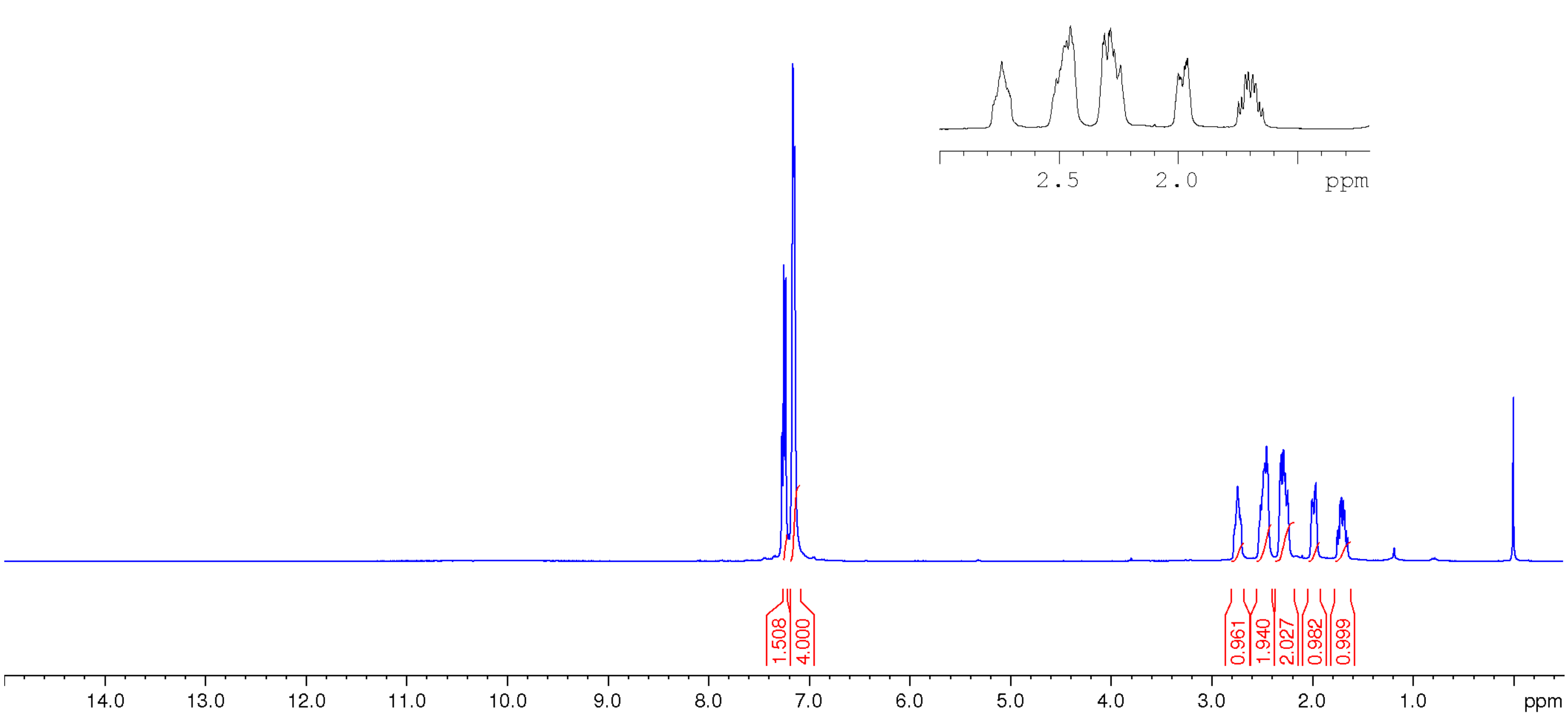

^{13}C NMR-spectrum (100 MHz, $CDCl_3$)

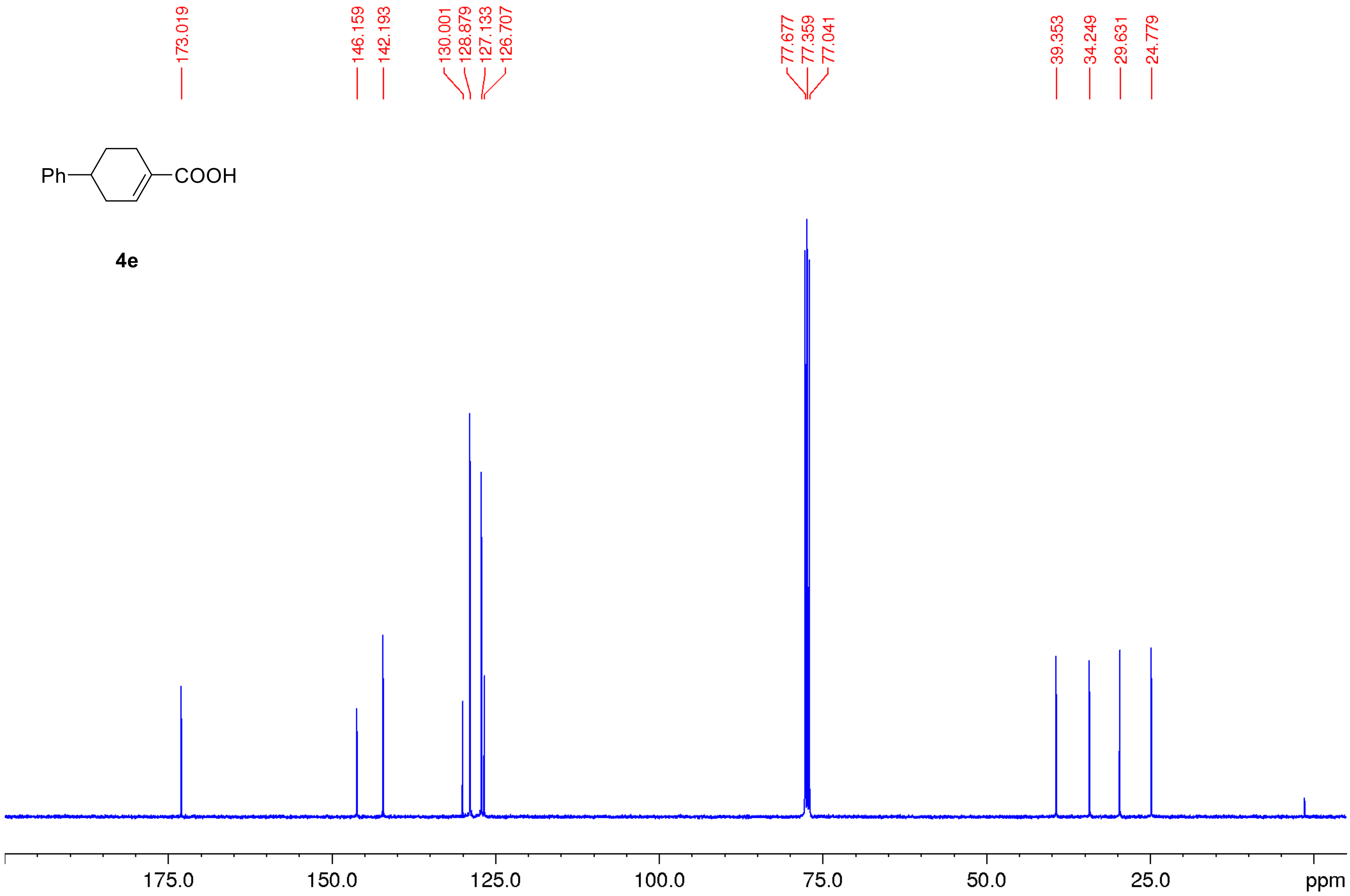

DEPT 135 NMR-spectrum ($CDCl_3$)

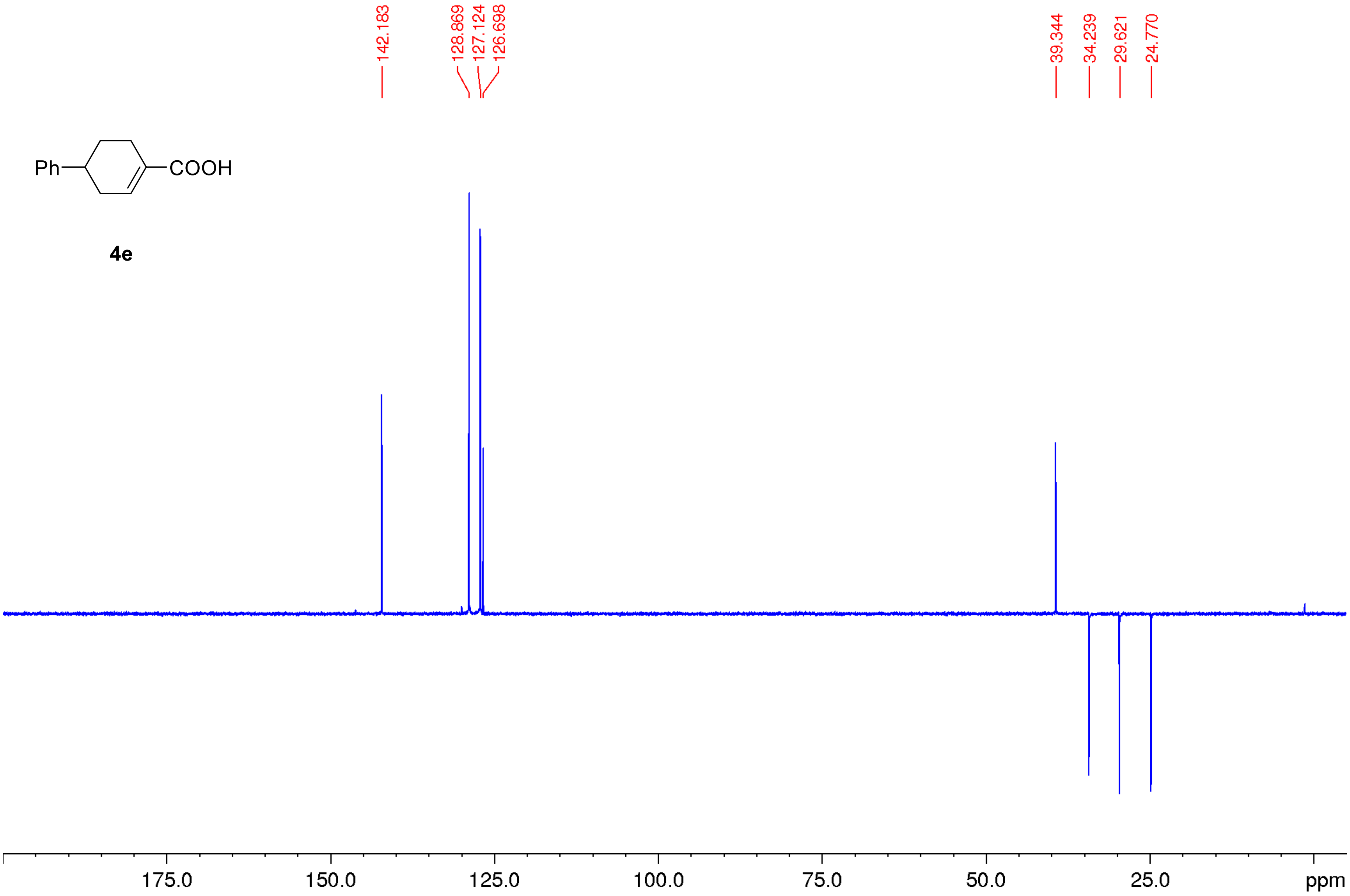

4e

^{1}H NMR-spectrum (400 MHz, $CDCl_3$)

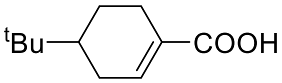

4f

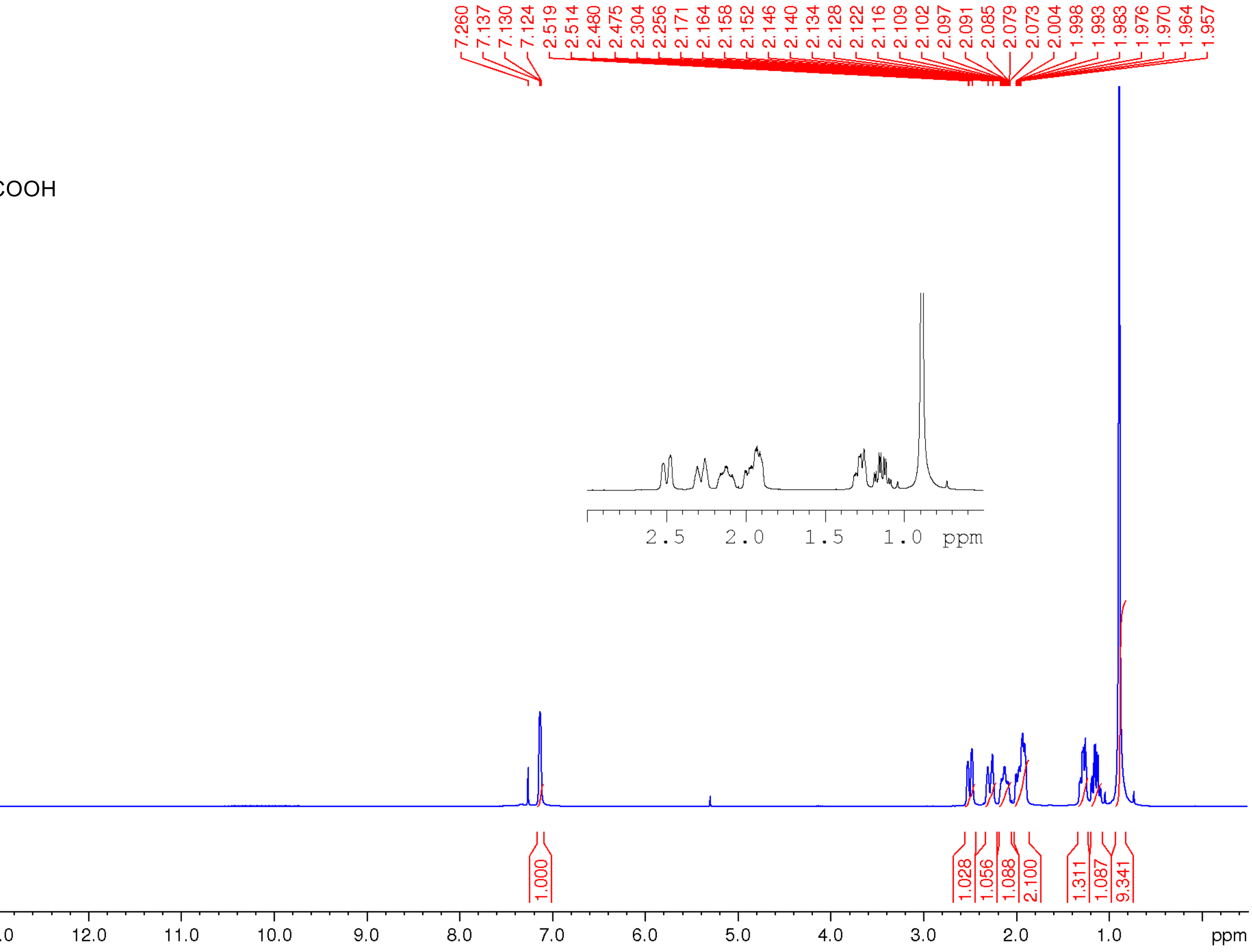

^{13}C NMR-spectrum (100 MHz, $CDCl_3$)

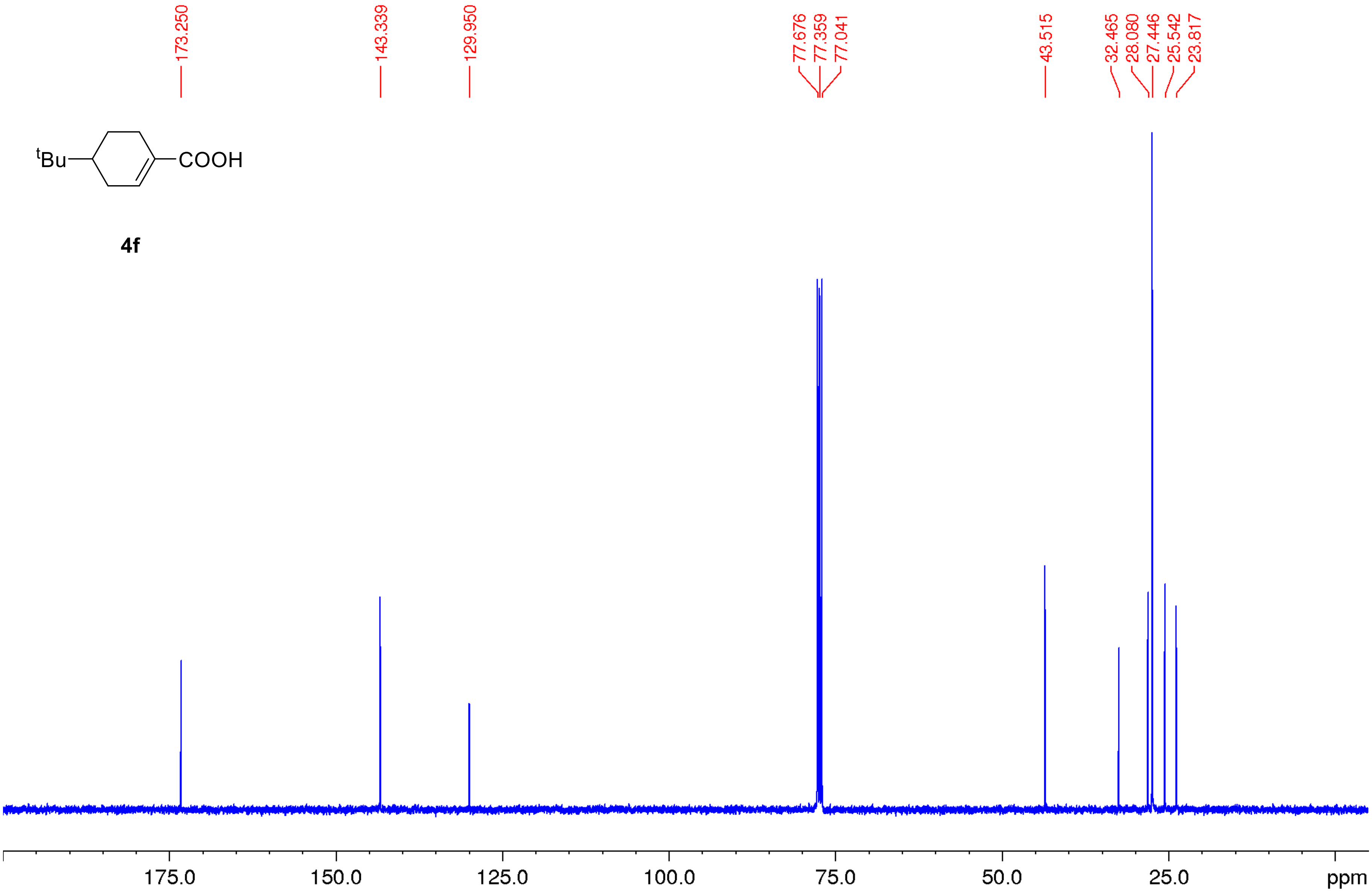

4f

DEPT 135 NMR-spectrum ($CDCl_3$)

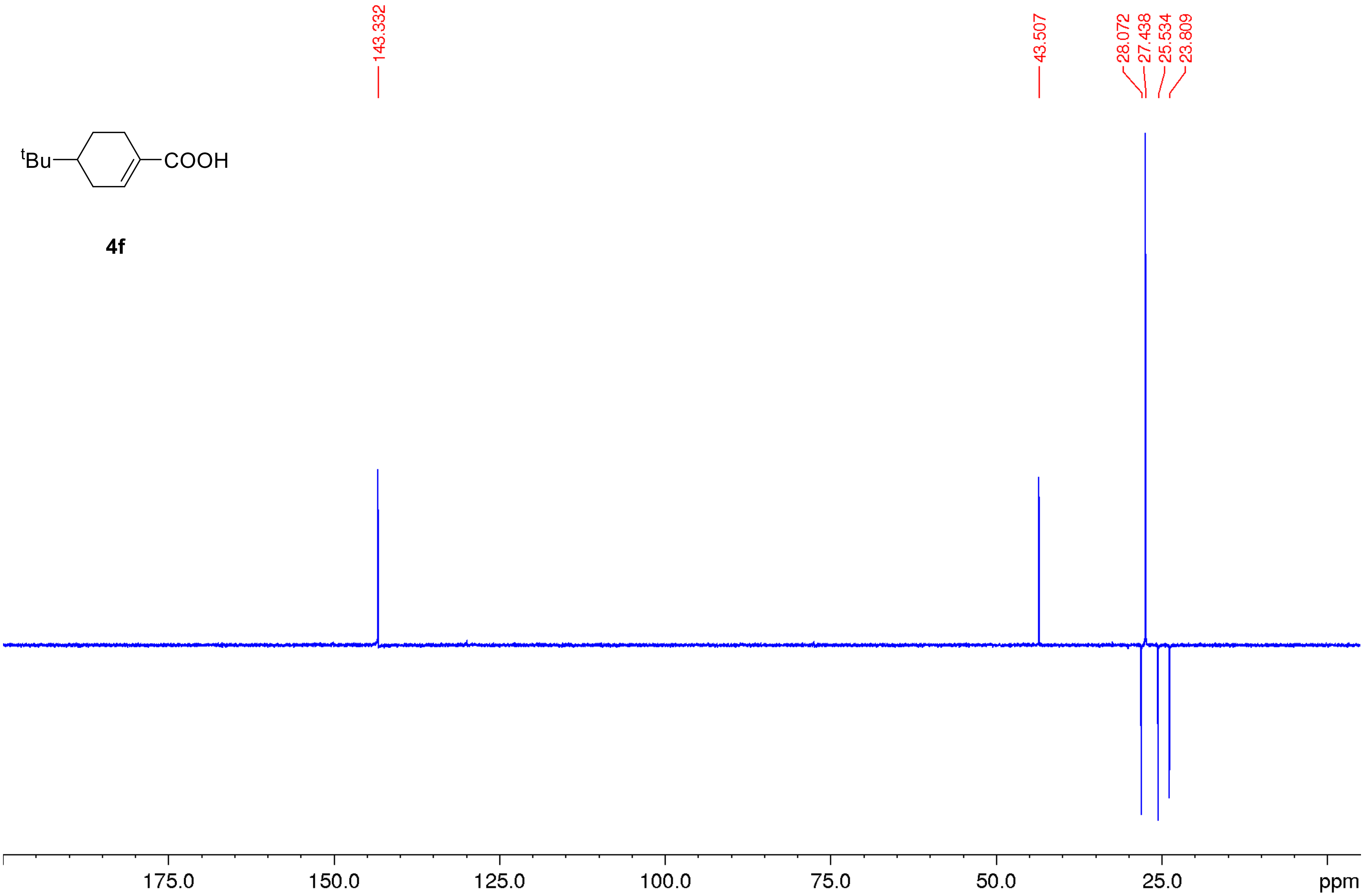

4f

^{1}H NMR-spectrum (400 MHz, $CDCl_3$)

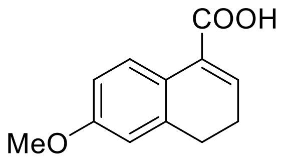

4g

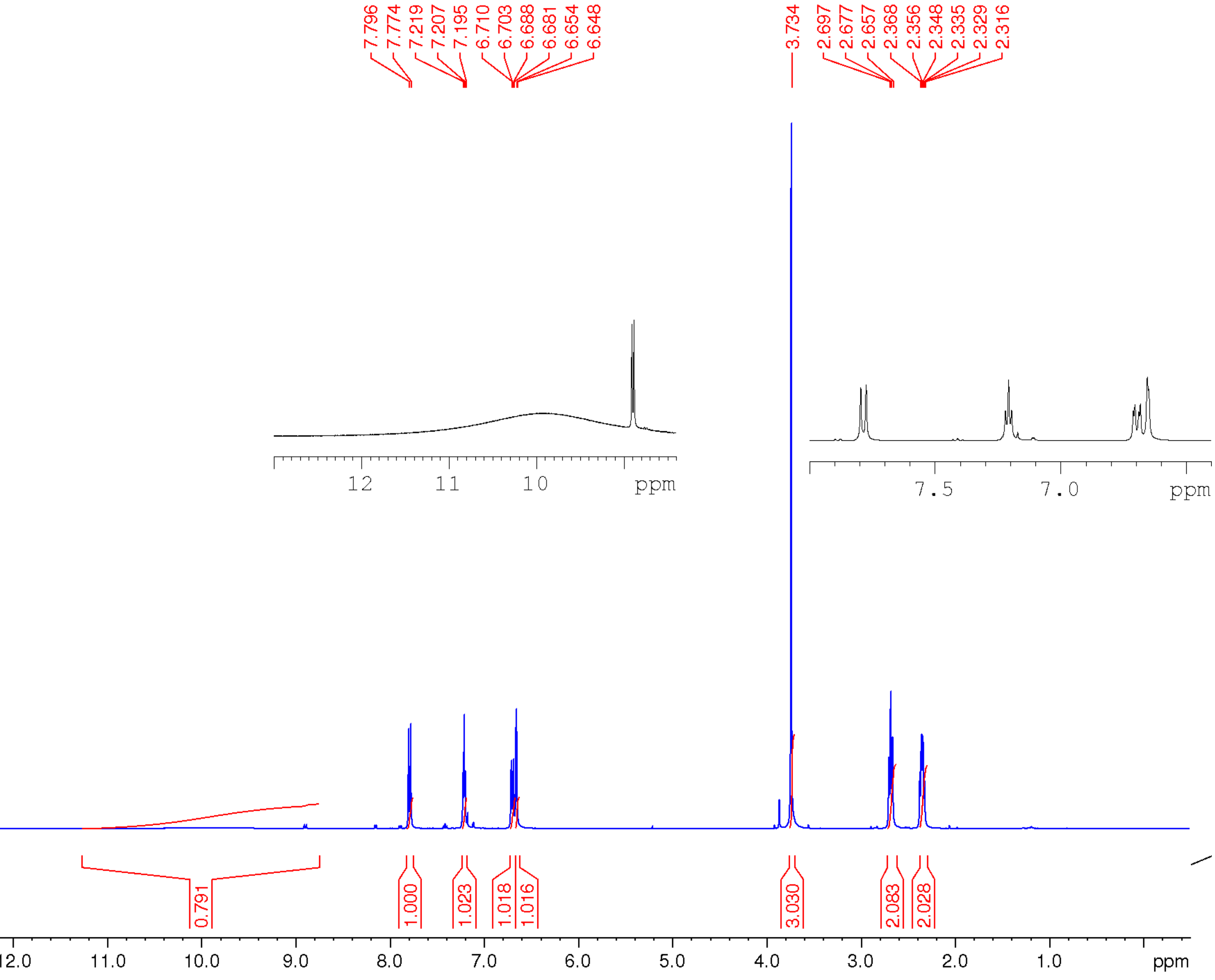

^{13}C NMR-spectrum (100 MHz, $CDCl_3$)

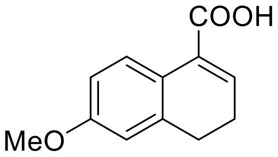

4g

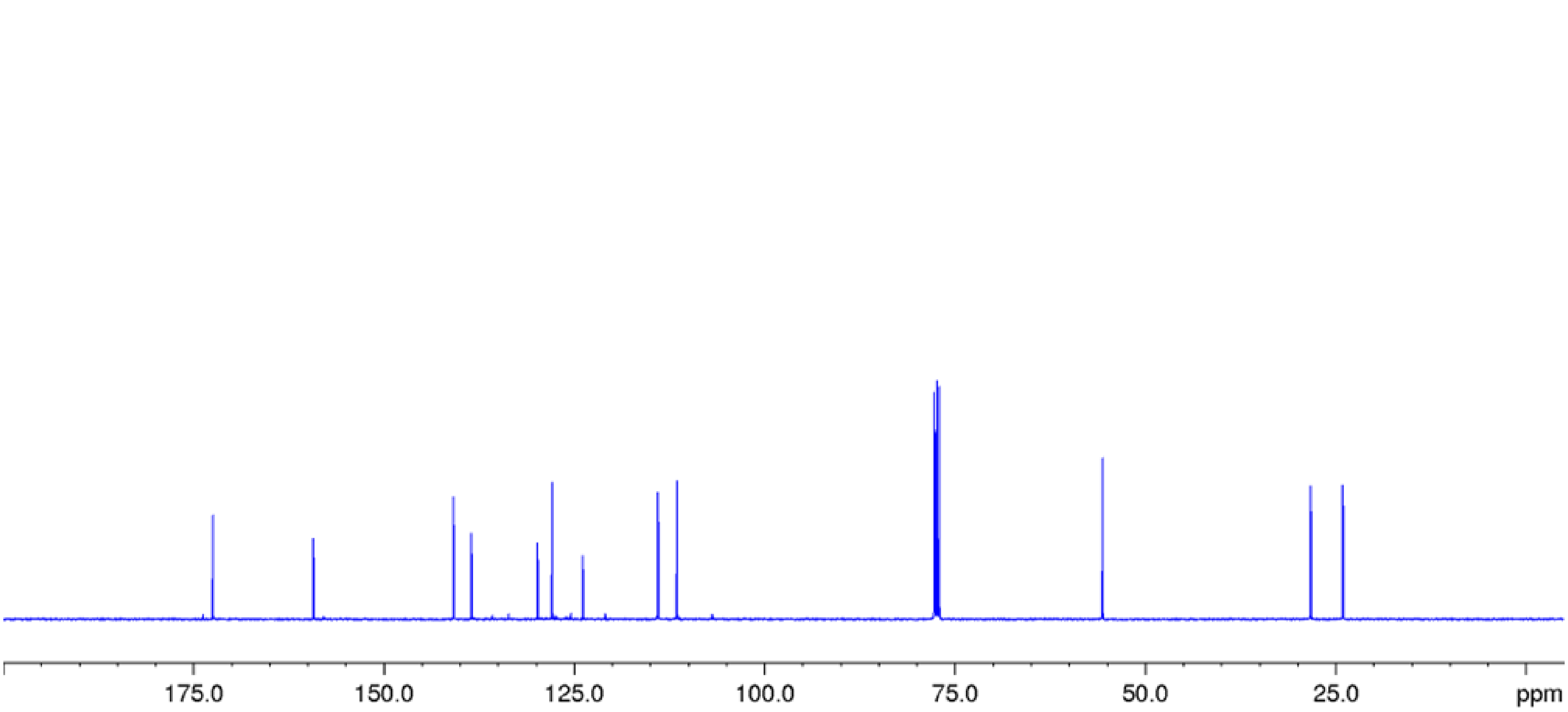

DEPT 135 NMR-spectrum ($CDCl_3$)

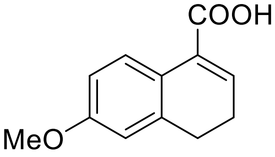

4g

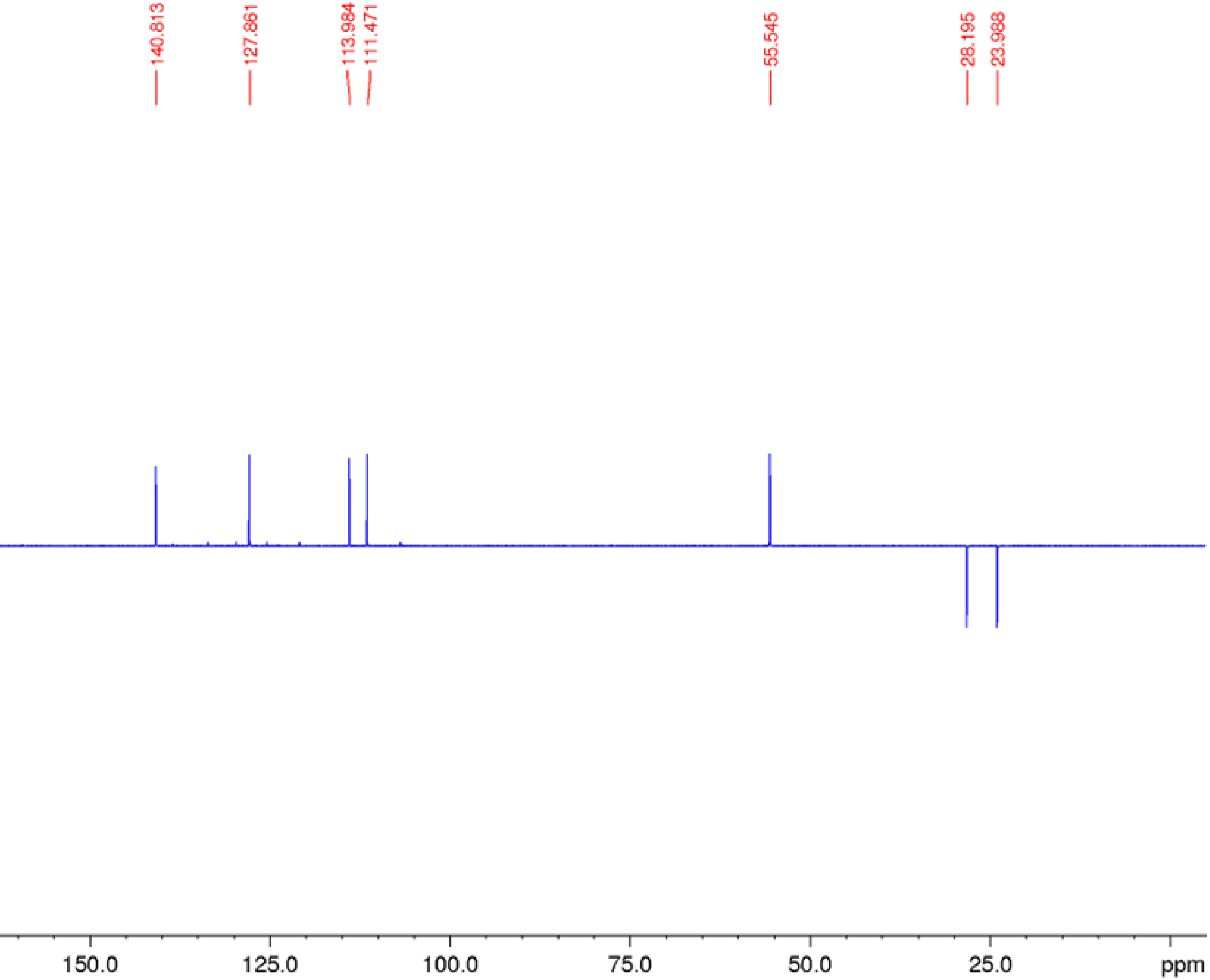

^{1}H NMR-spectrum (400 MHz, $CDCl_3$)

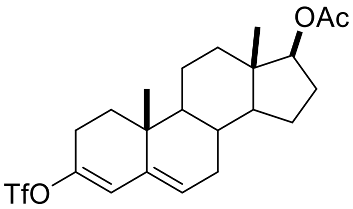

3a

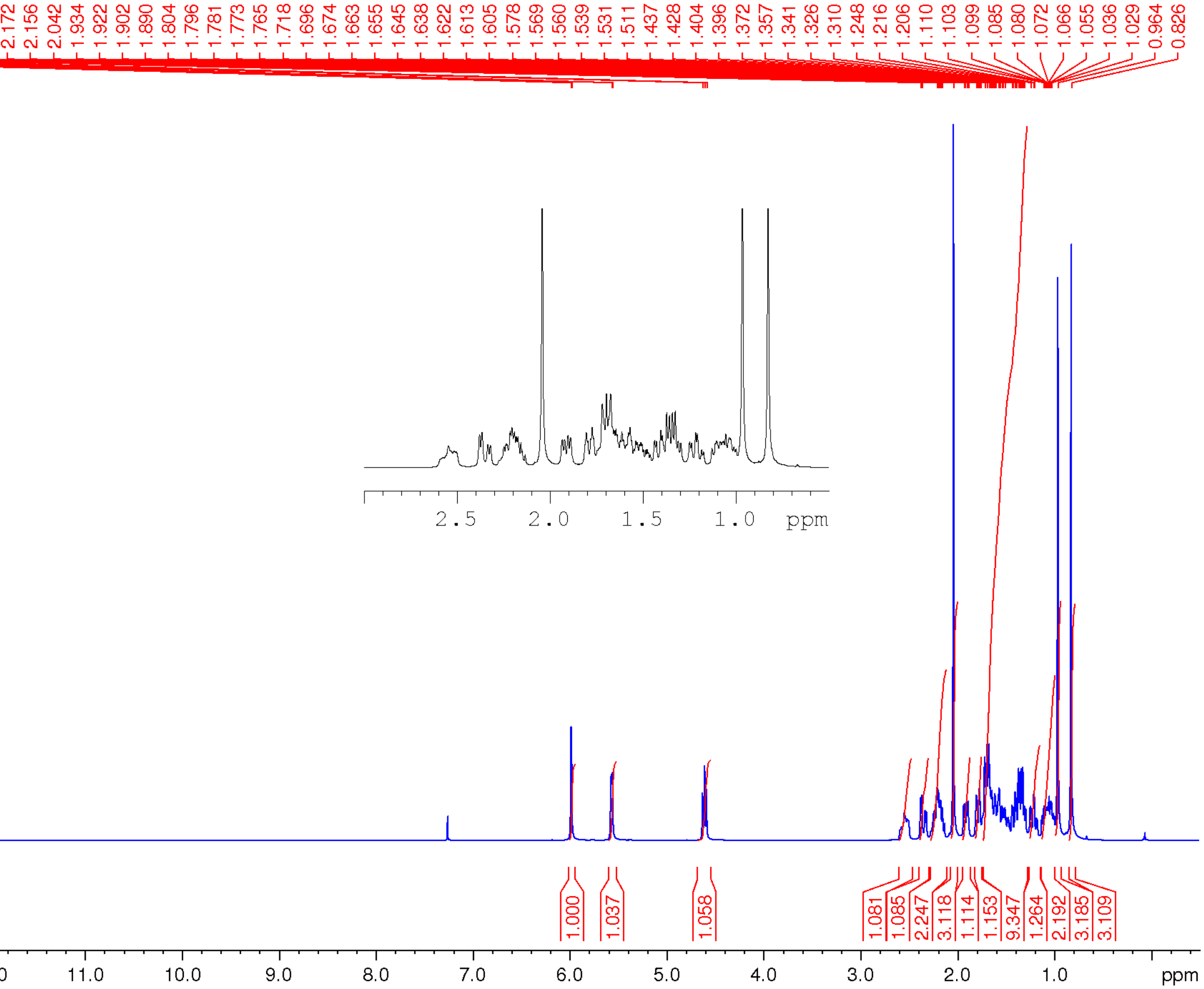

^{13}C NMR-spectrum (100 MHz, $CDCl_3$)

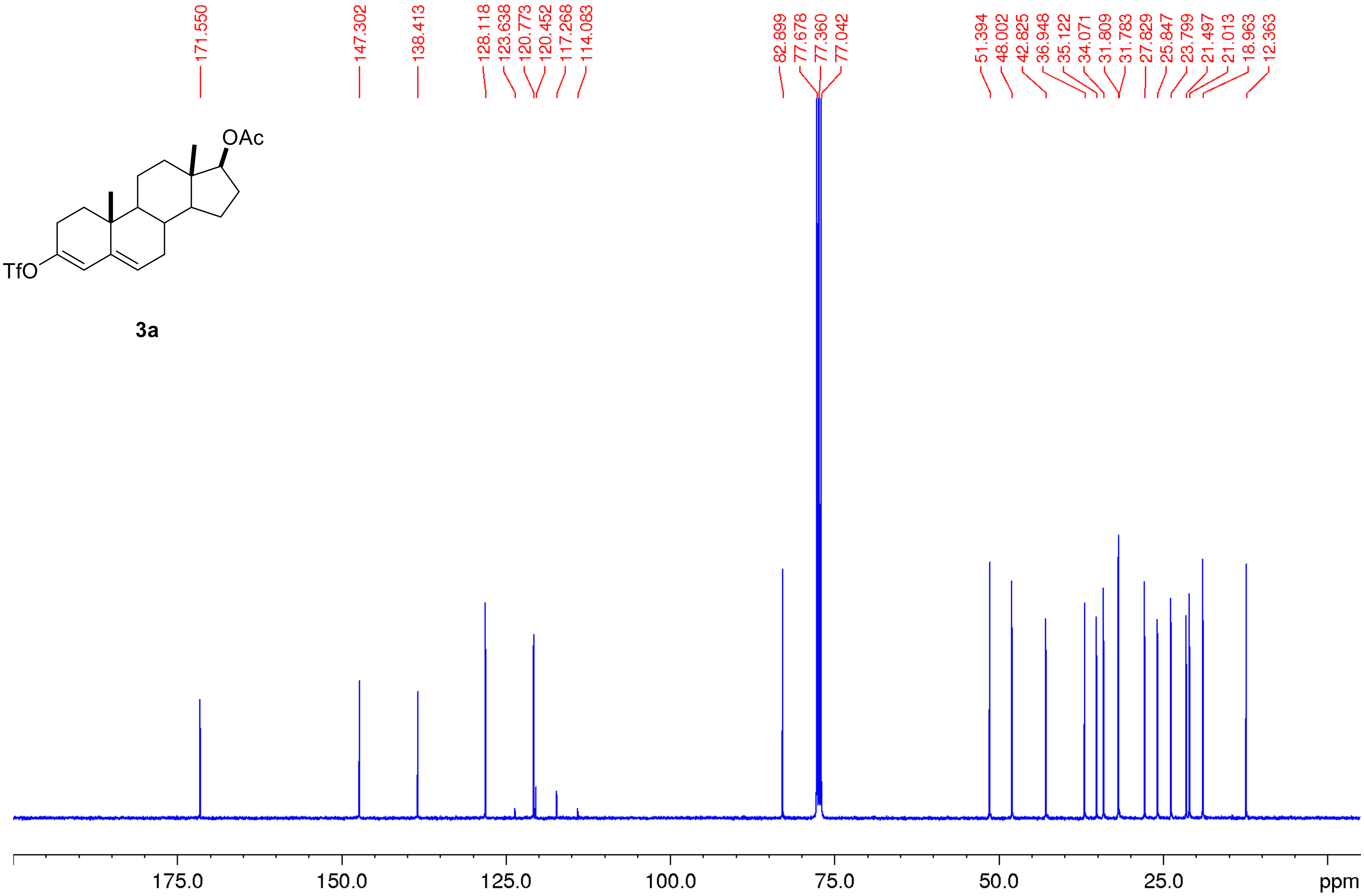

DEPT 135 NMR-spectrum ($CDCl_3$)

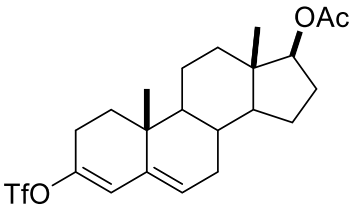

3a

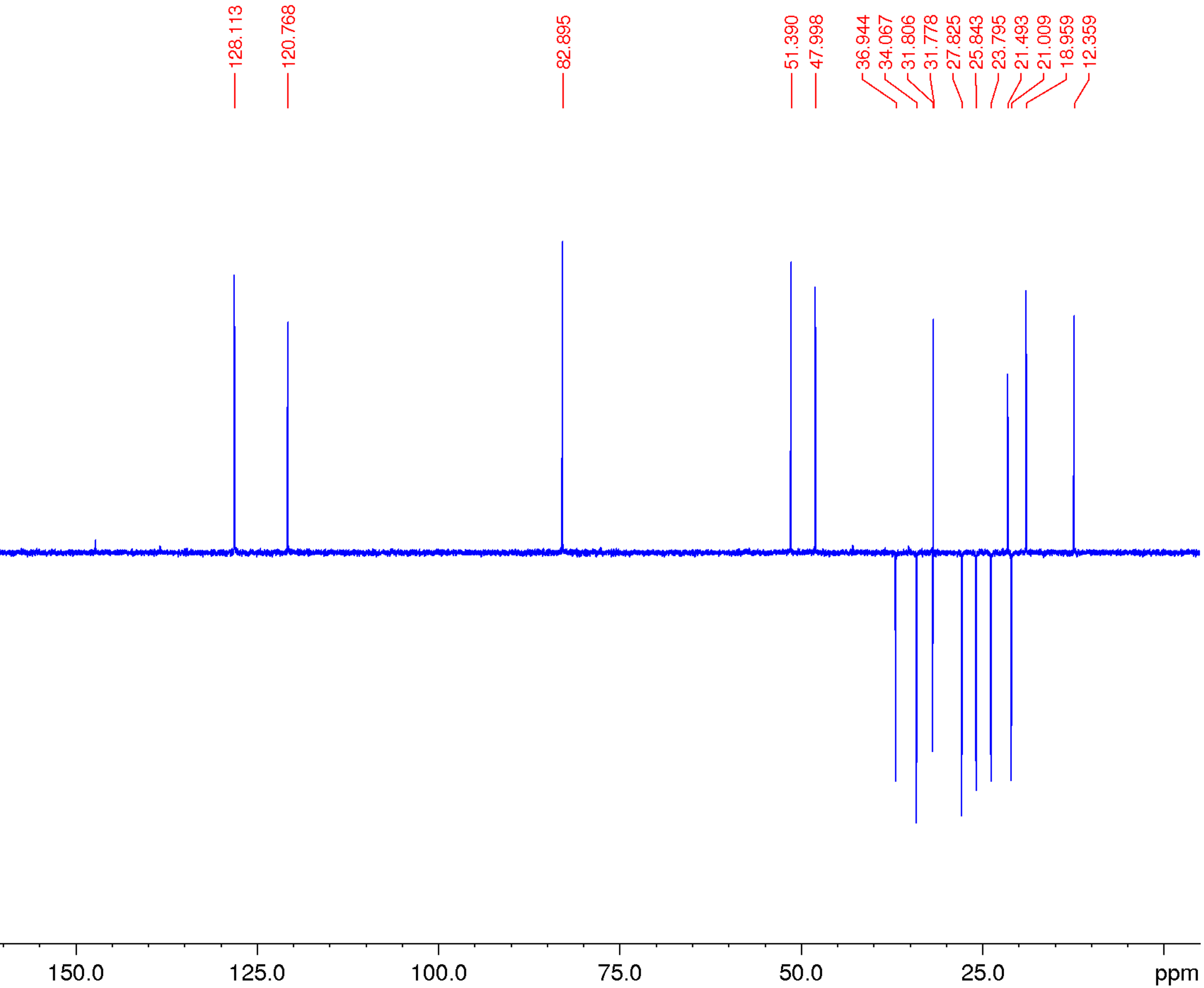

^{19}F NMR-spectrum (376.5 Hz, $CDCl_3$)

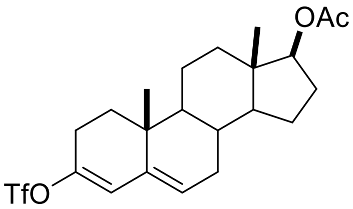

3a

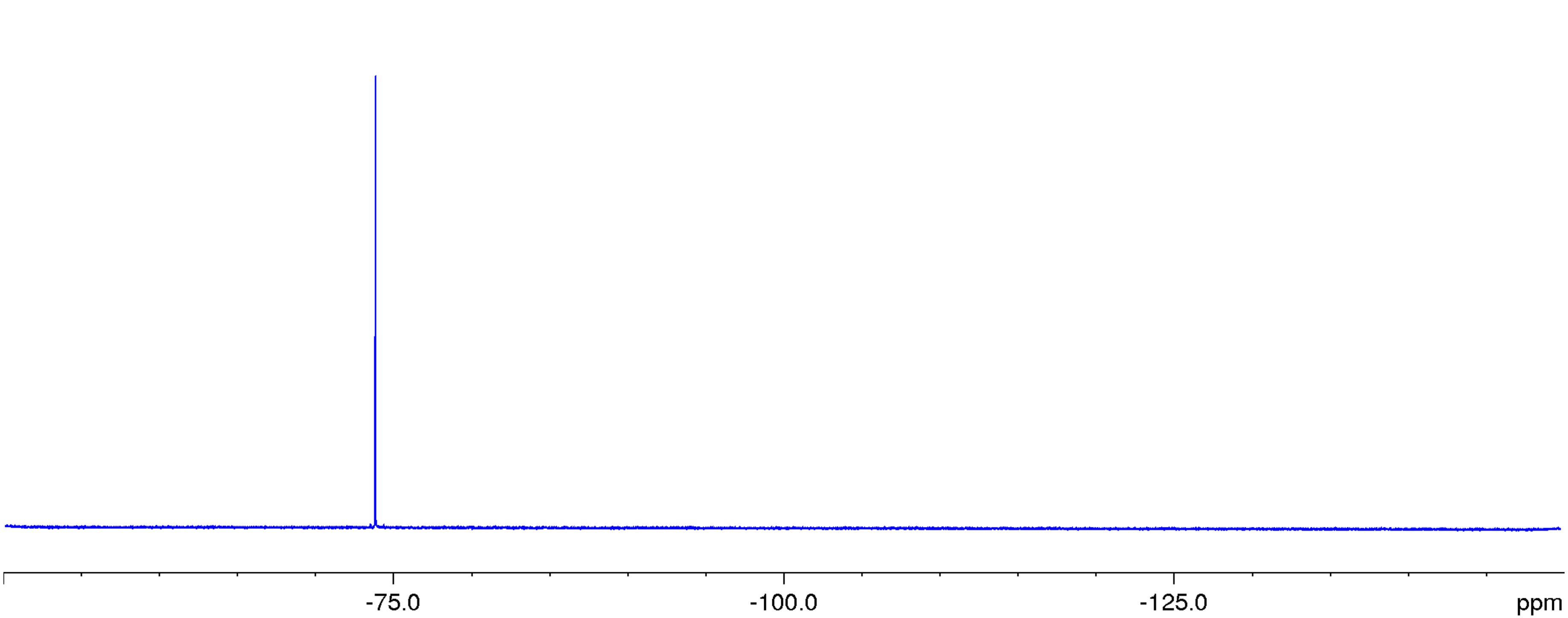

^{1}H NMR-spectrum (400 MHz, $CDCl_3$)

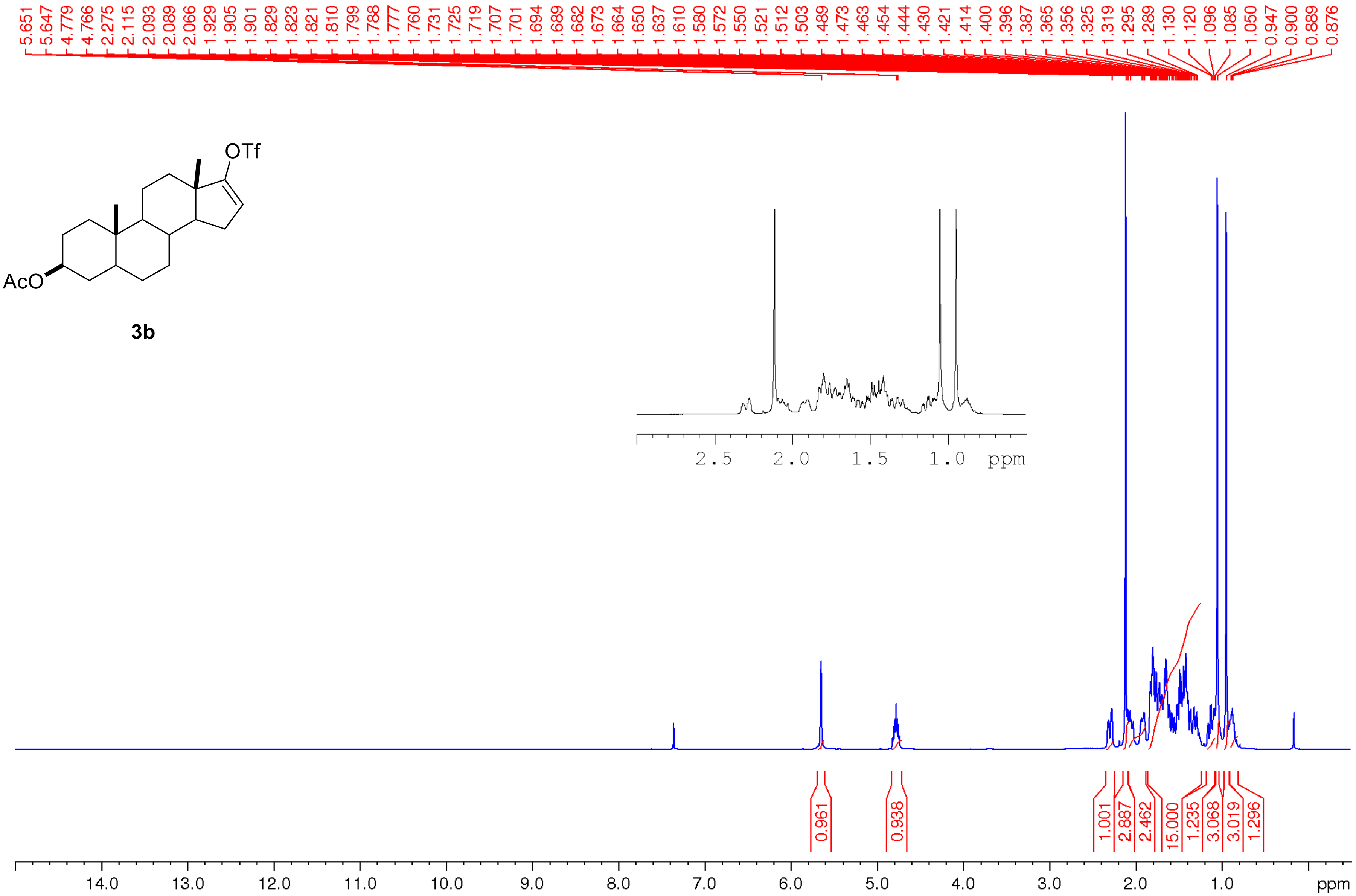

^{13}C NMR-spectrum (100 MHz, $CDCl_3$)

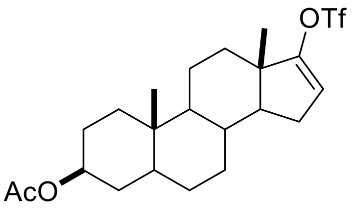

3b

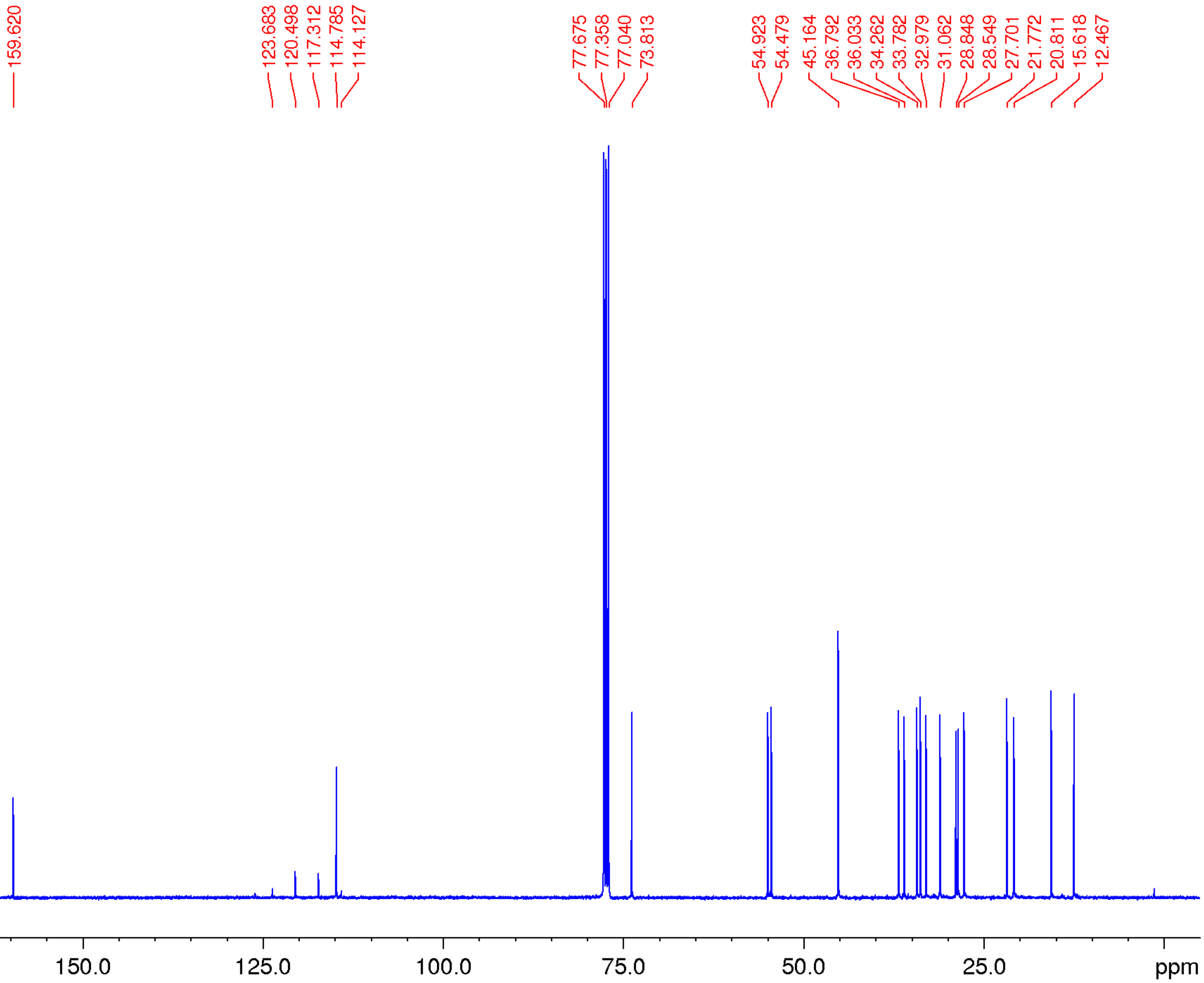

DEPT 135 NMR-spectrum ($CDCl_3$)

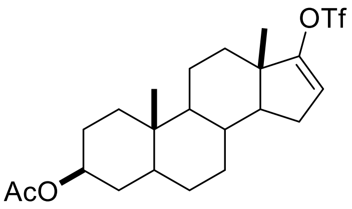

3b

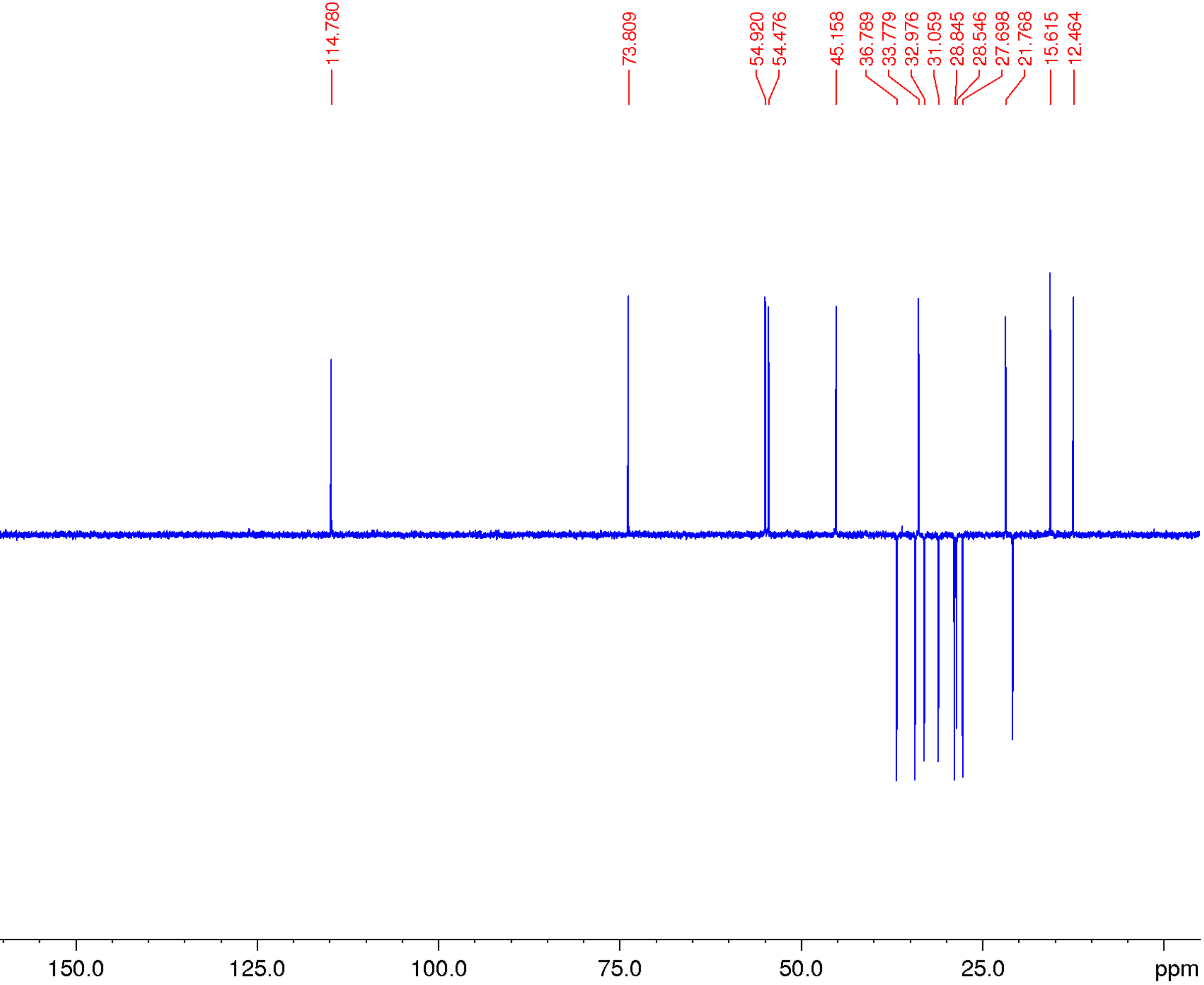

^{19}F NMR-spectrum (376.5 Hz, $CDCl_3$)

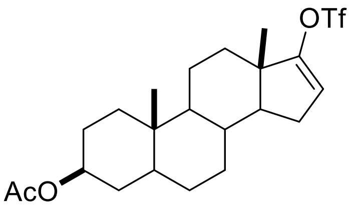

3b

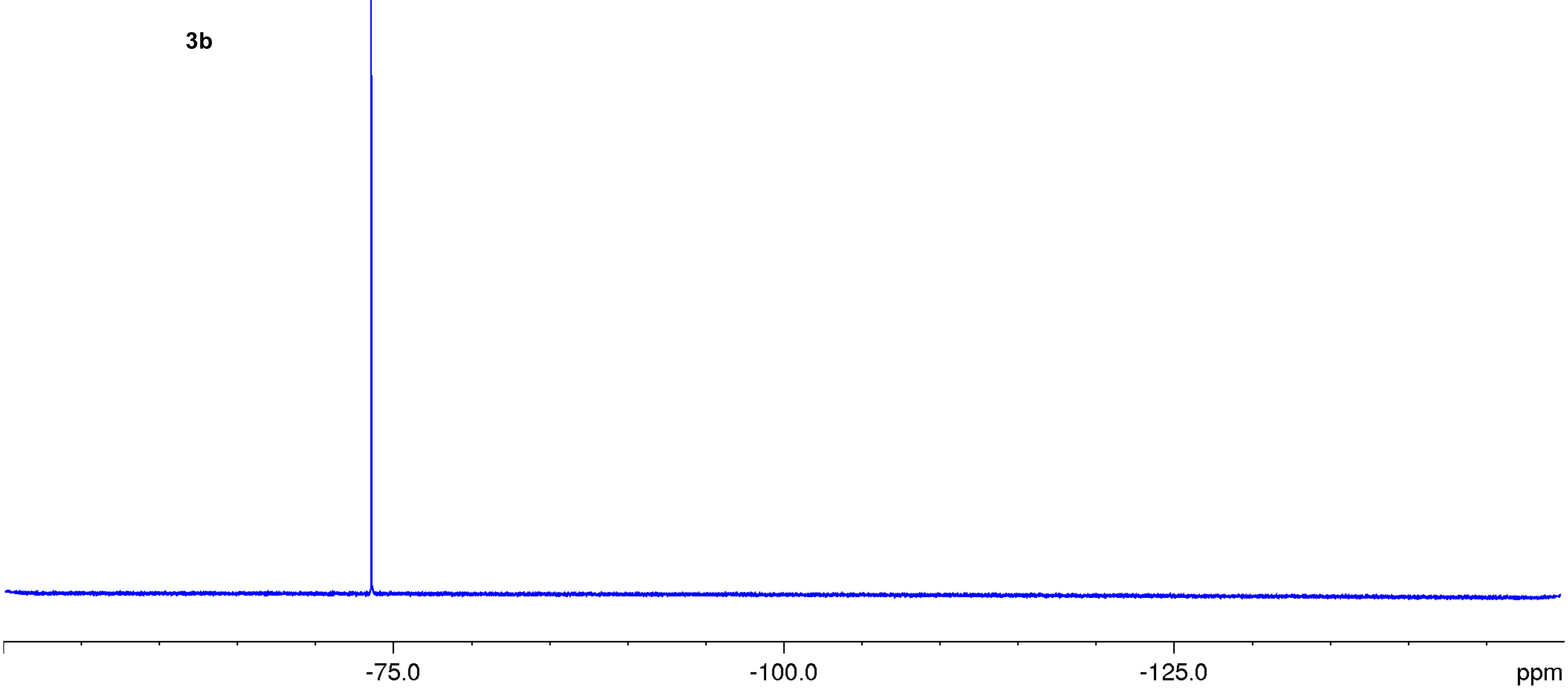

^{1}H NMR-spectrum (400 MHz, $CDCl_3$)

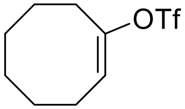

3c

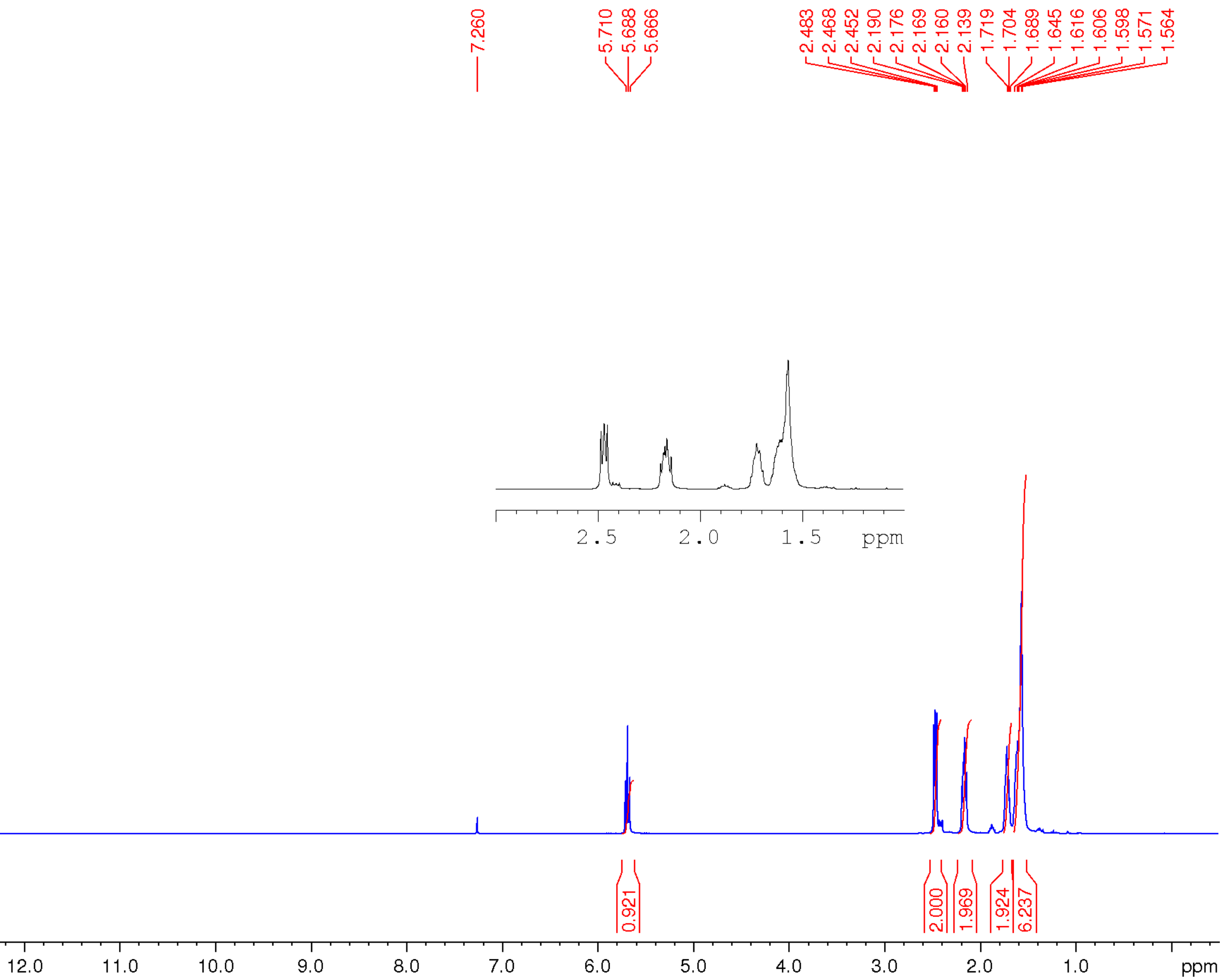

^{13}C NMR-spectrum (100 MHz, $CDCl_3$)

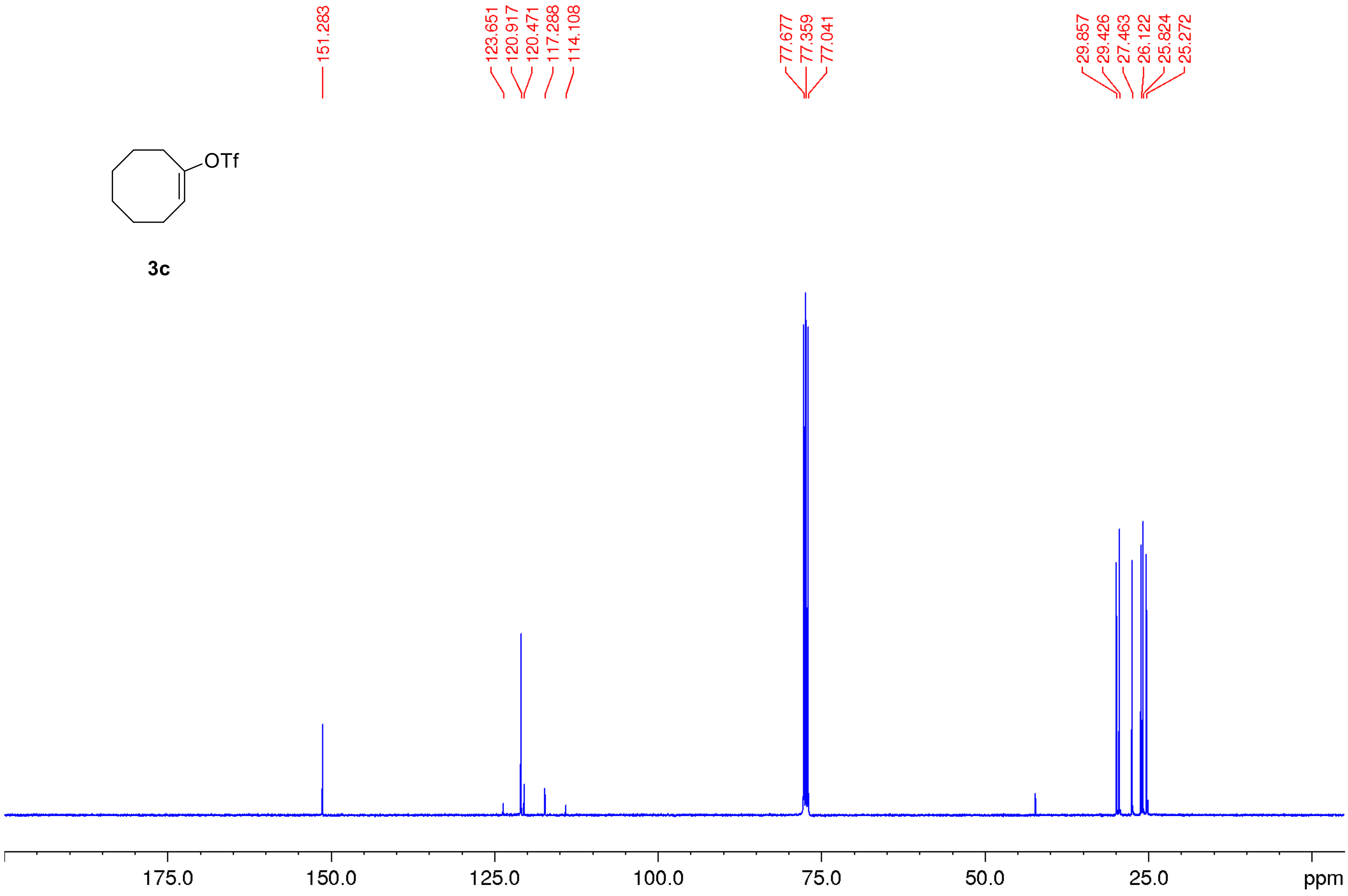

3c

DEPT 135 NMR-spectrum ($CDCl_3$)

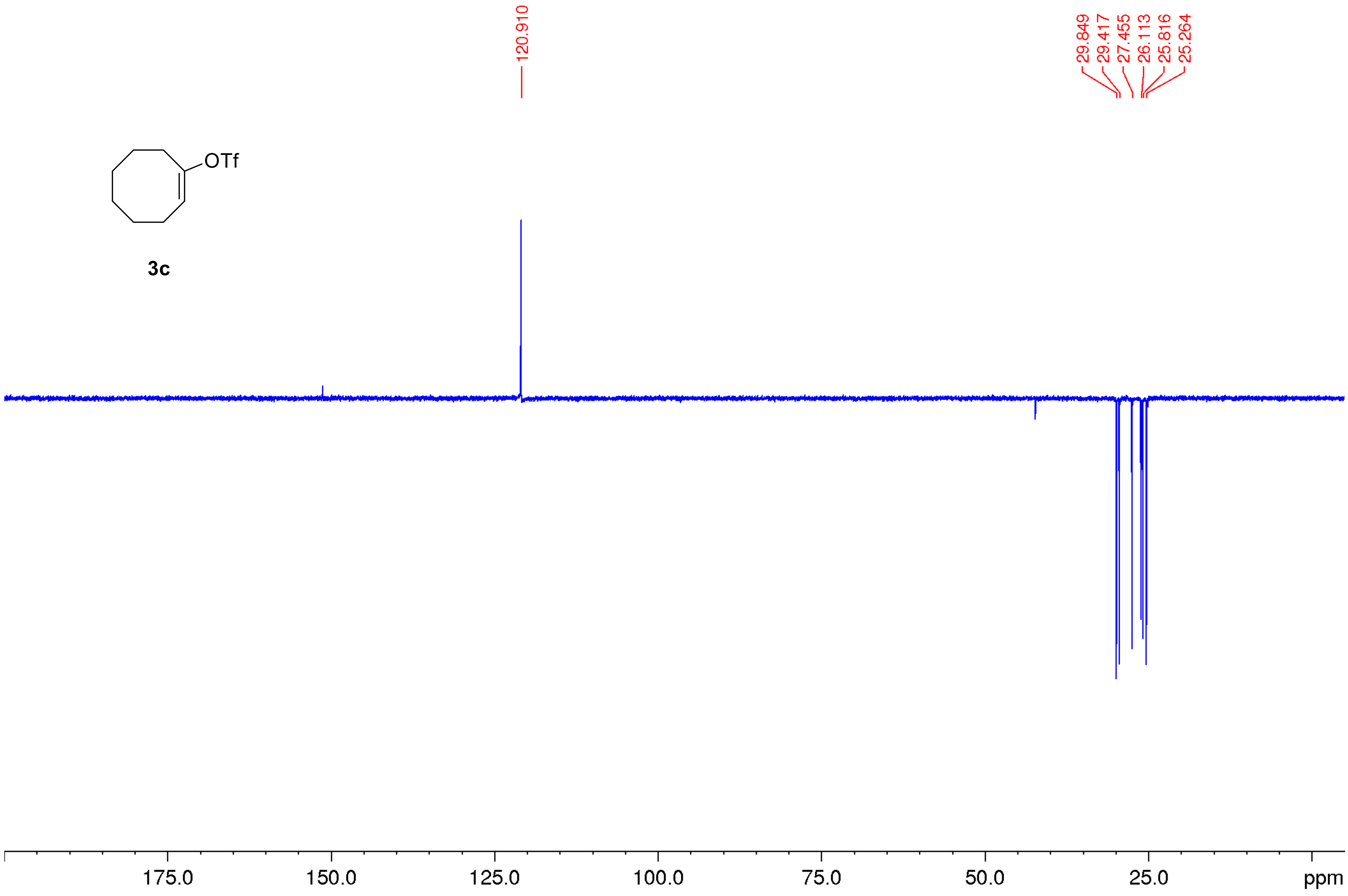

^{19}F NMR-spectrum (376.5 Hz, $CDCl_3$)

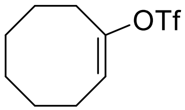

3c

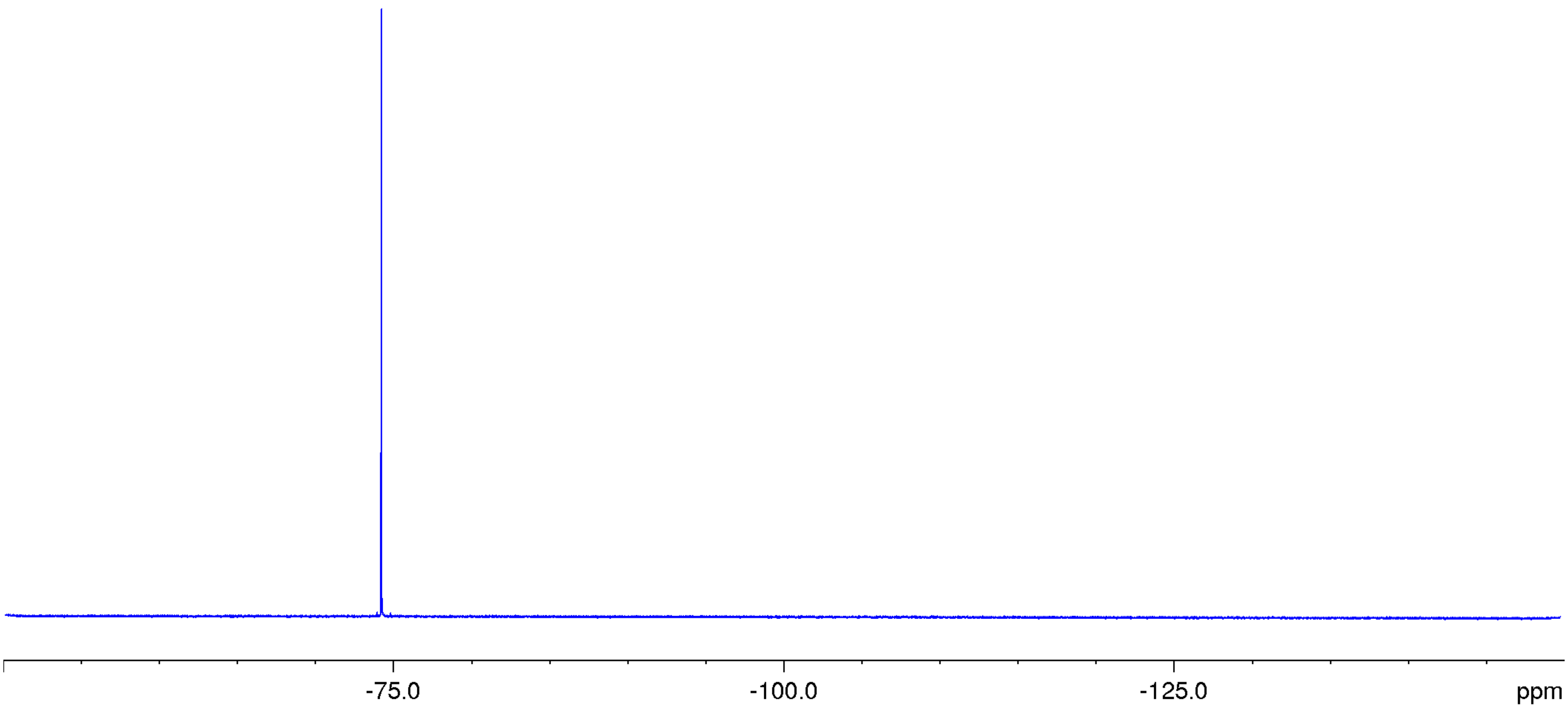

^{1}H NMR-spectrum (400 MHz, $CDCl_3$)

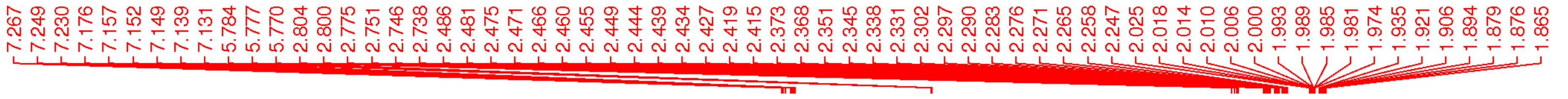

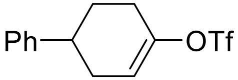

3e

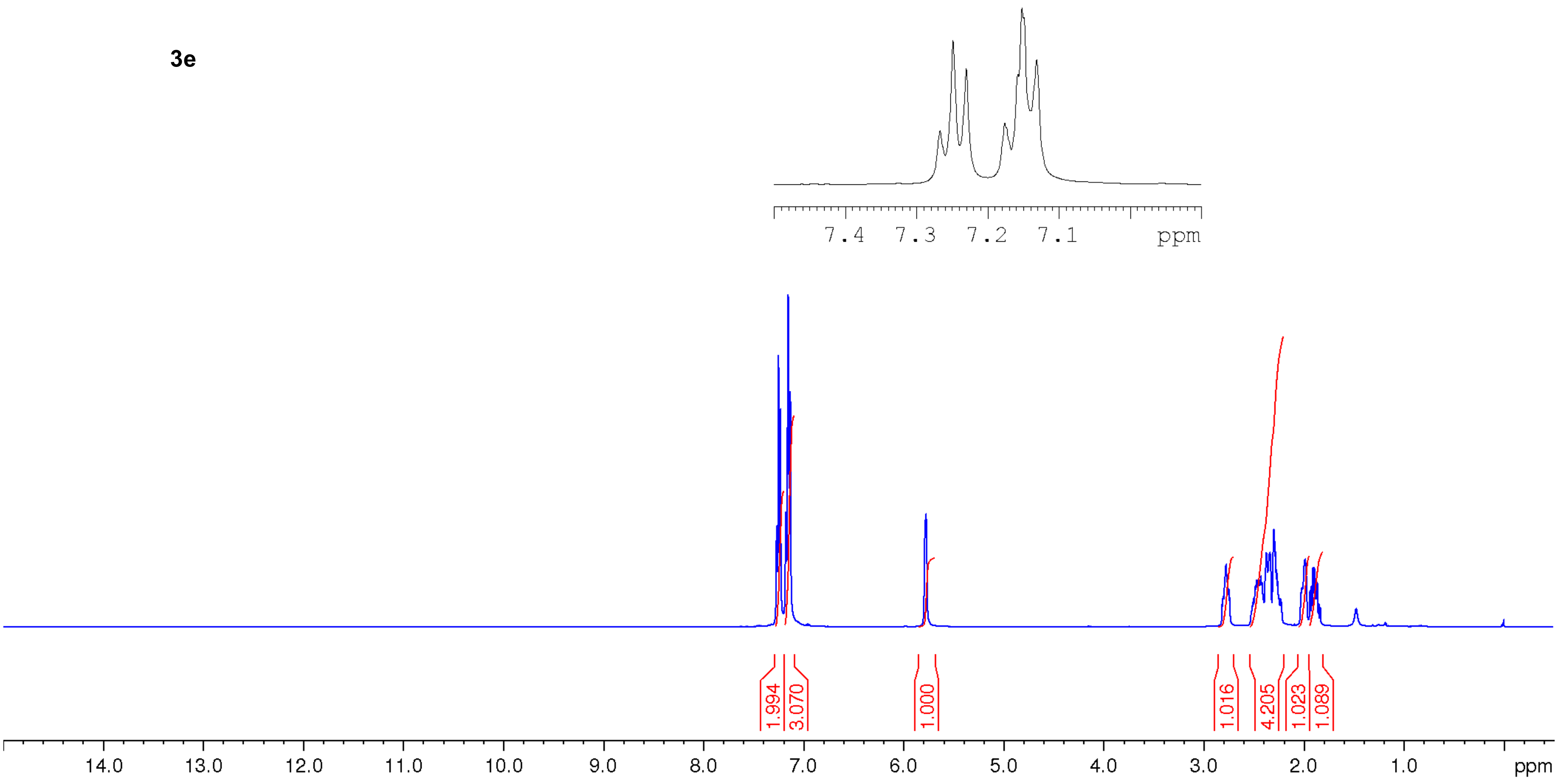

^{13}C NMR-spectrum (100 MHz, $CDCl_3$)

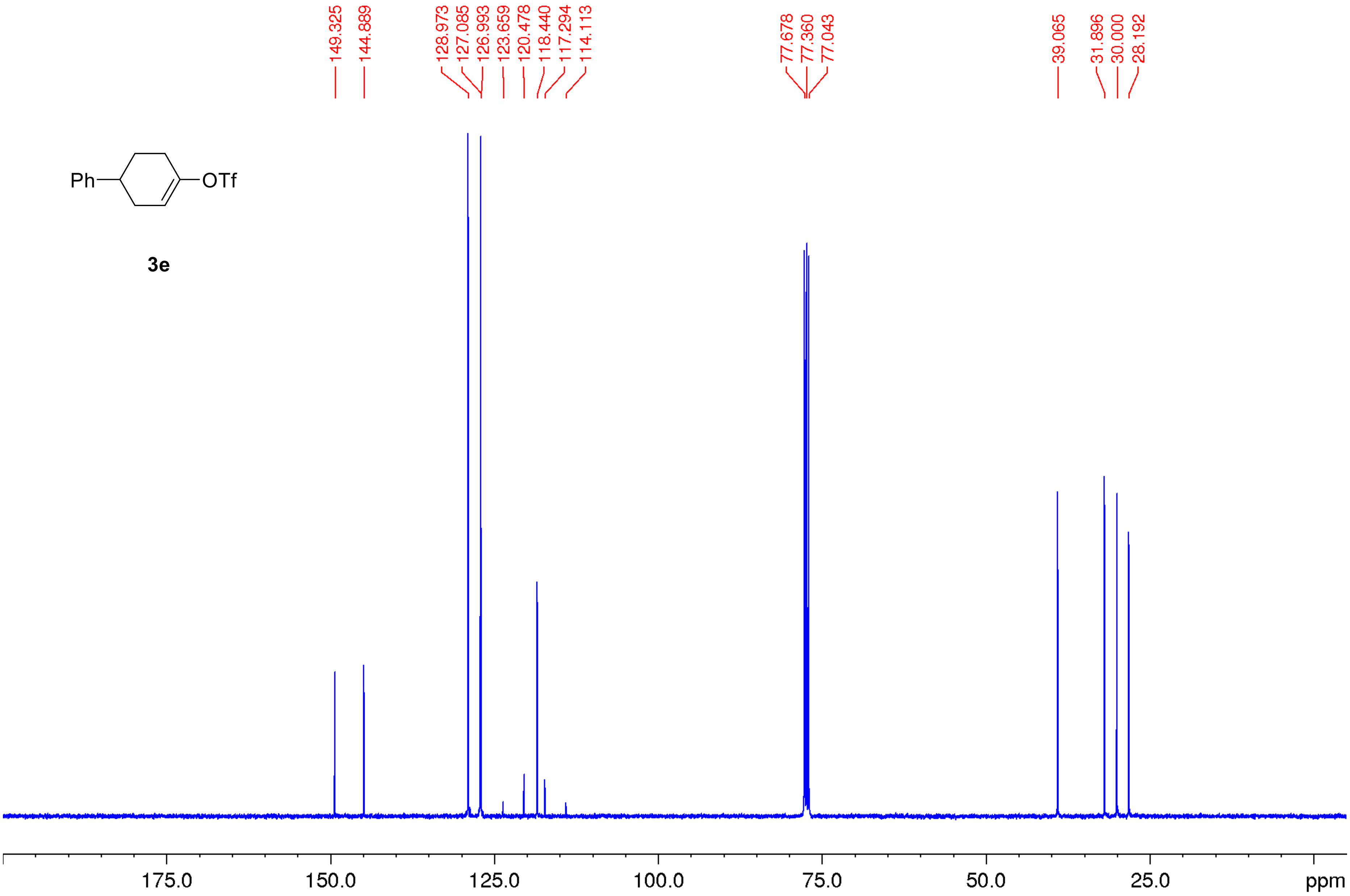

DEPT 135 NMR-spectrum ($CDCl_3$)

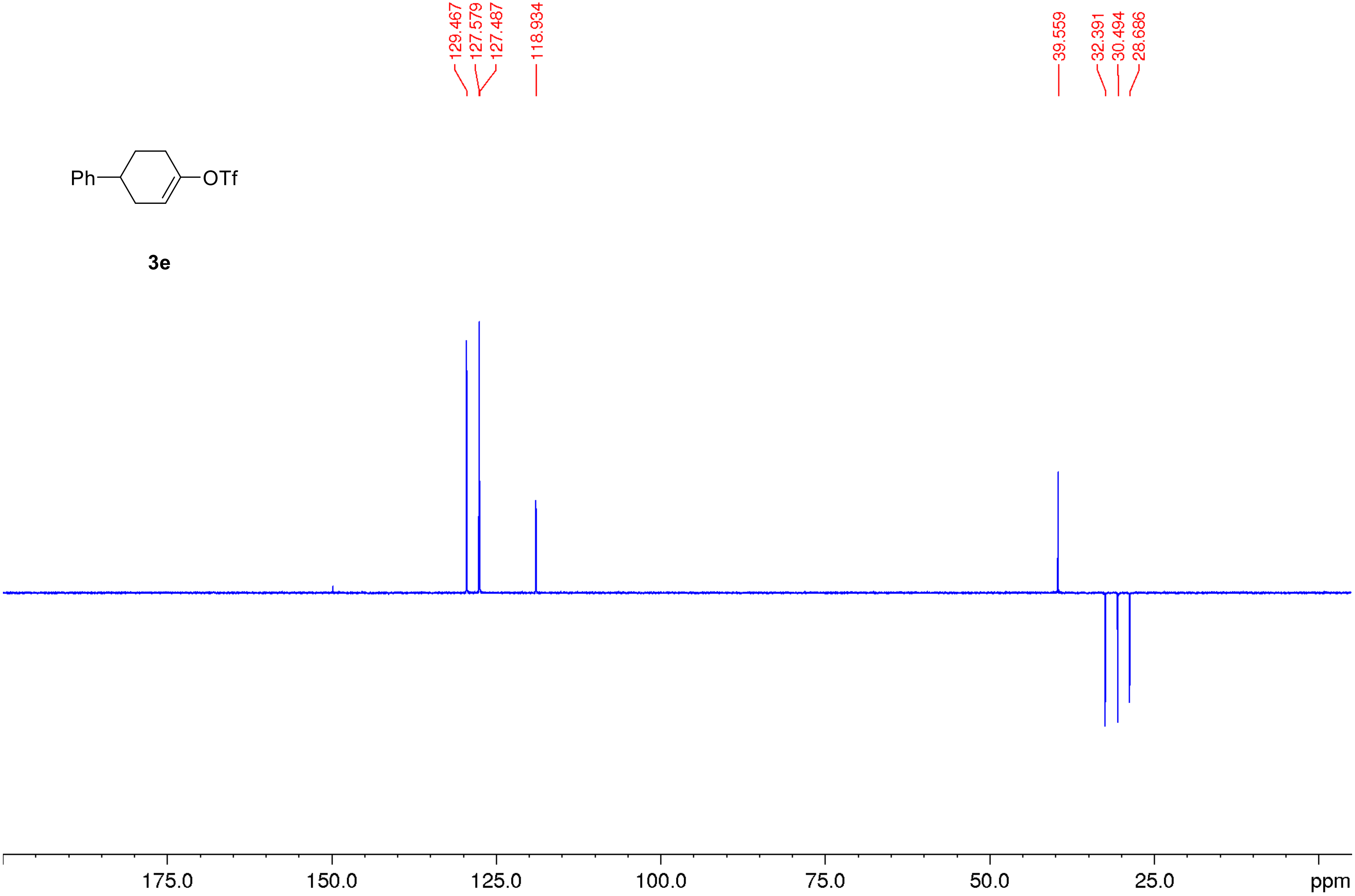

^{19}F NMR-spectrum (376.5 Hz, $CDCl_3$)

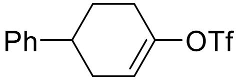

3e

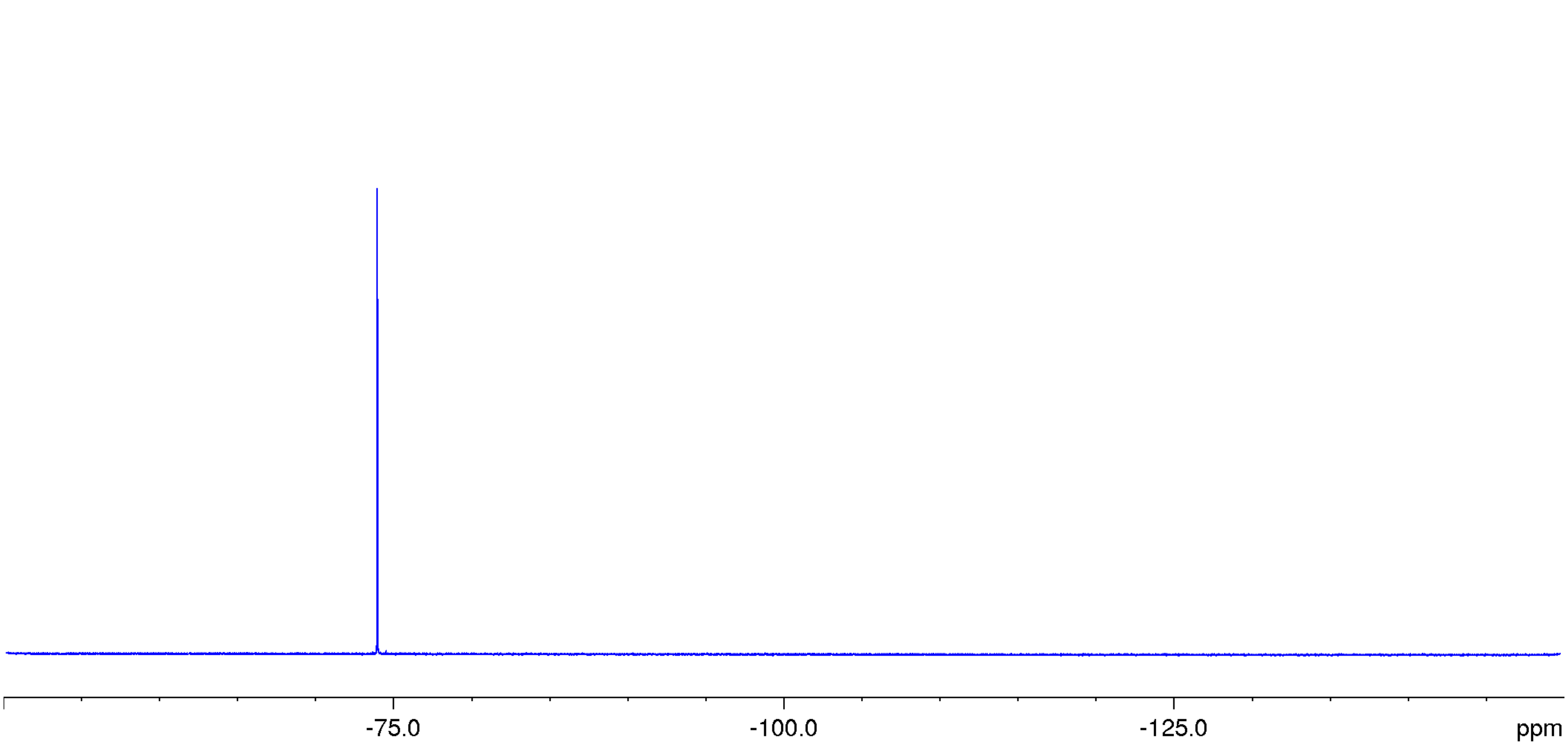

^{1}H NMR-spectrum (400 MHz, $CDCl_3$)

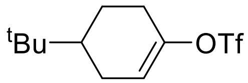

3f

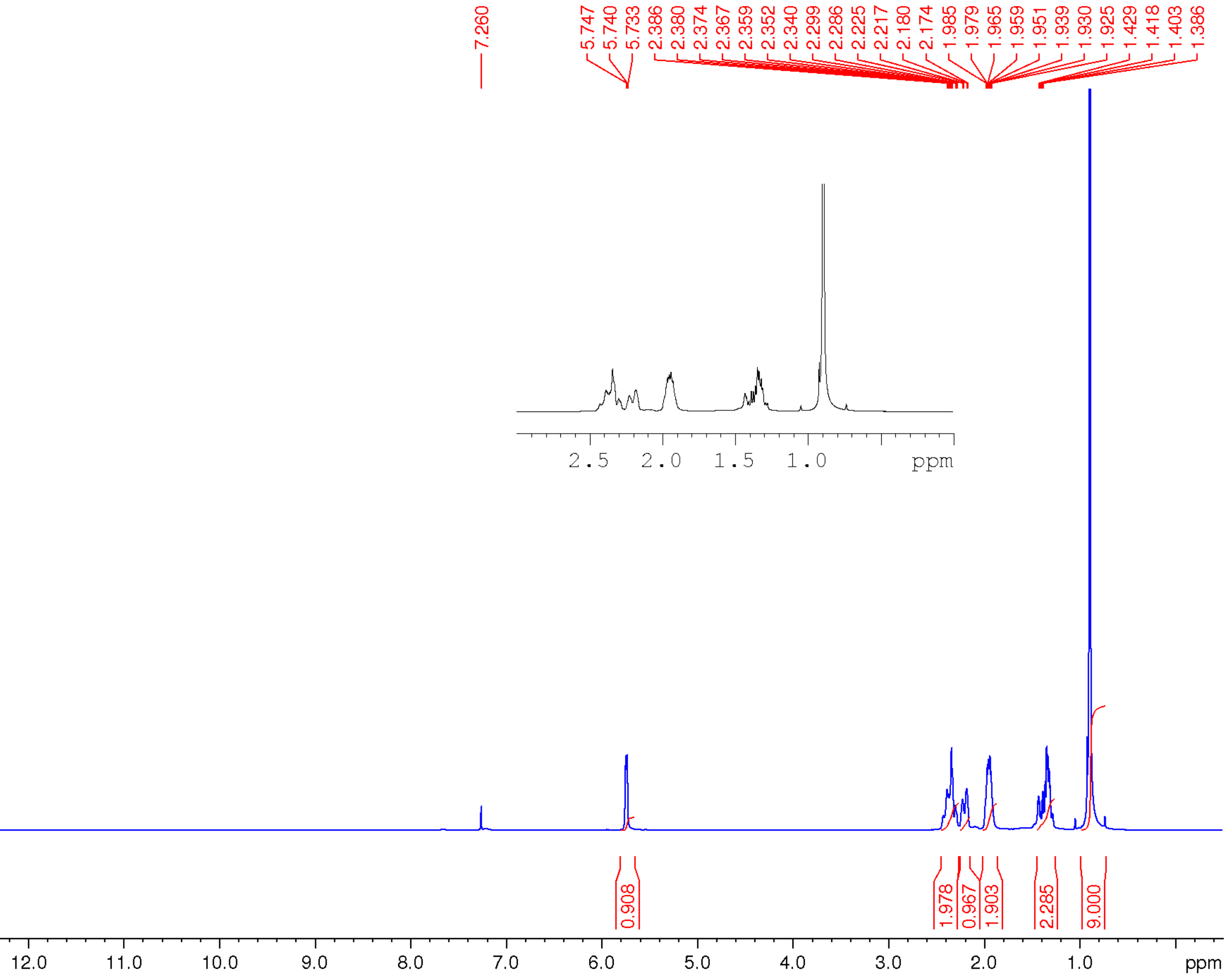

^{13}C NMR-spectrum (100 MHz, $CDCl_3$)

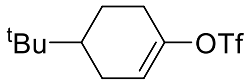

3f

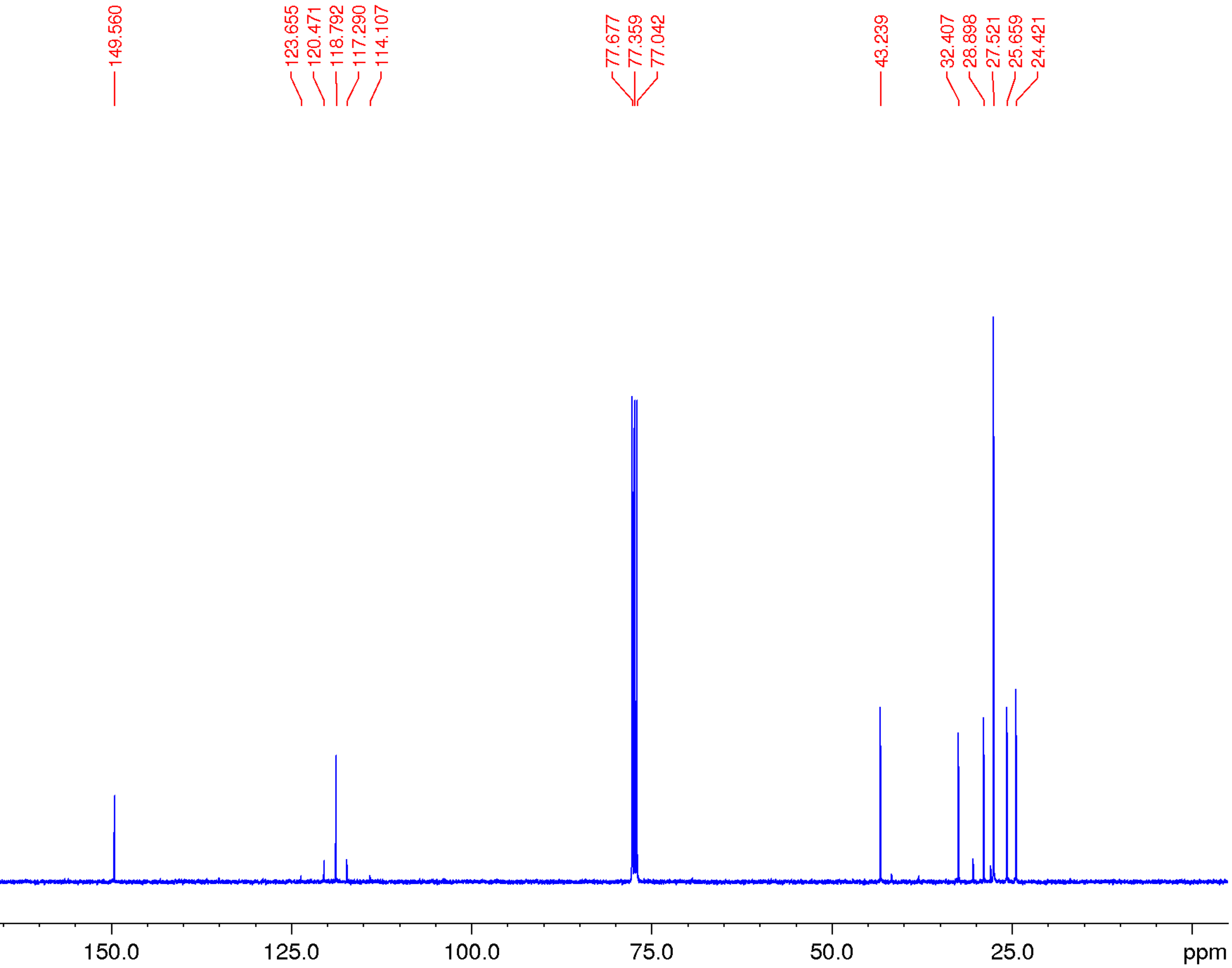

DEPT 135 NMR-spectrum ($CDCl_3$)

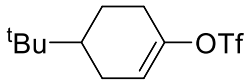

3f

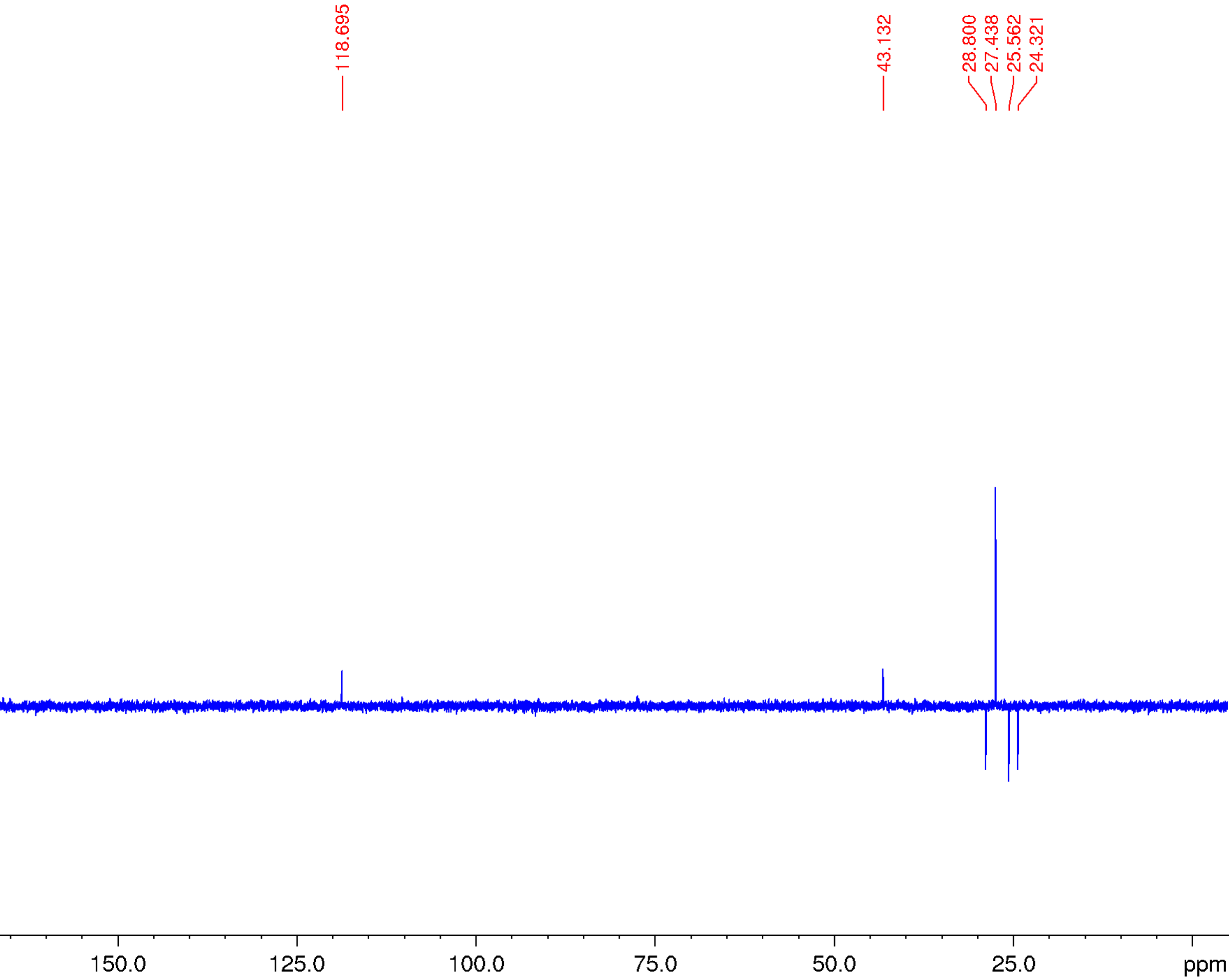

^{19}F NMR-spectrum (376.5 Hz, $CDCl_3$)

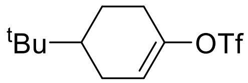

3f

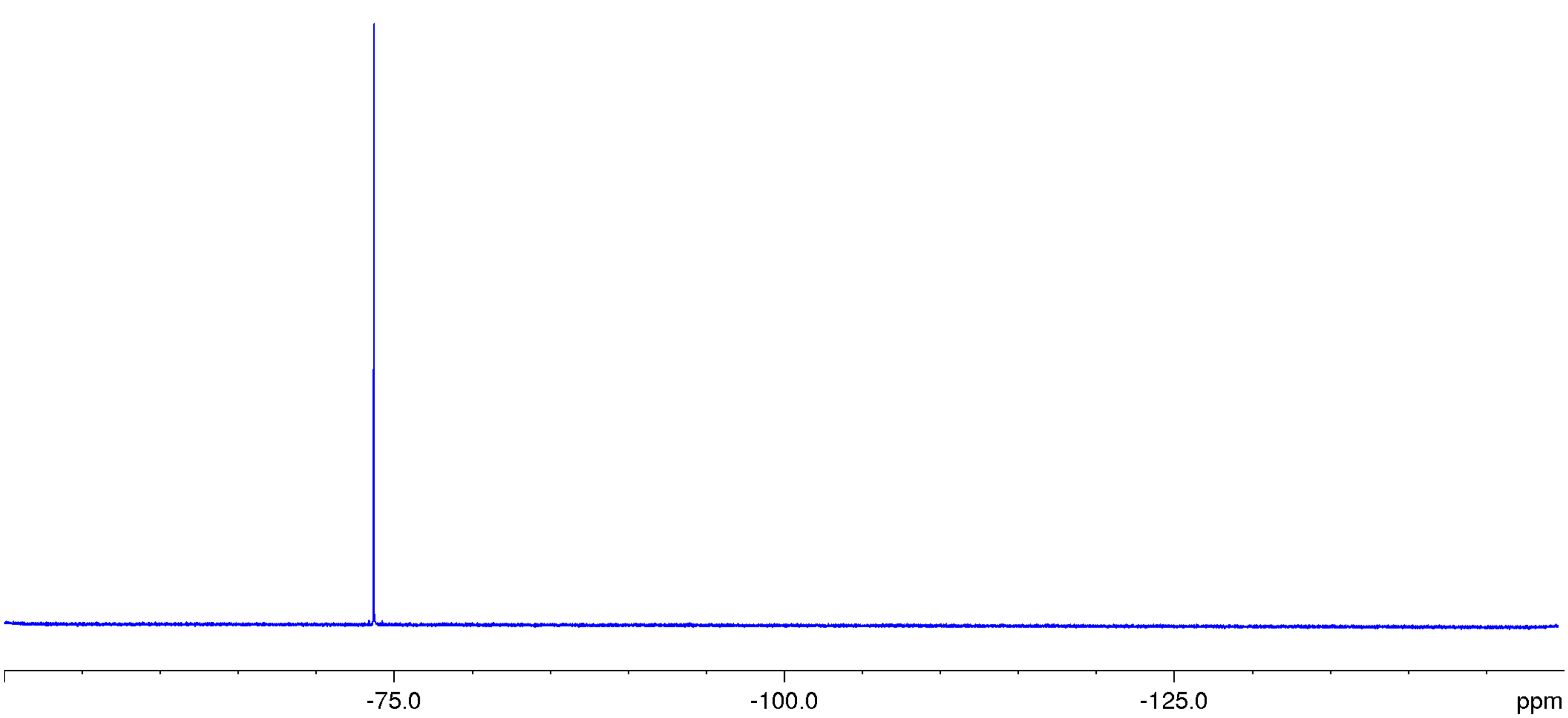

Supplement: Supplementary file 1 [file molecules-30-03298-s001.zip › molecules-3754818-supplementary.pdf]
